# First total synthesis of concavine†
†Electronic supplementary information (ESI) available. CCDC 1523963. For ESI and crystallographic data in CIF or other electronic format see DOI: 10.1039/c6sc05627j



**DOI:** 10.1039/c6sc05627j

**Published:** 2017-02-24

**Authors:** François Saint-Dizier, Nigel S. Simpkins

**Affiliations:** a School of Chemistry , University of Birmingham , Edgbaston , Birmingham , B15 2TT , UK . Email: n.simpkins@bham.ac.uk

## Abstract

The synthesis of the unusual alkaloid concavine, isolated from *Clitocybe concava* (Basidiomycetae), has been accomplished. The synthetic route features regio- and stereoselective manipulation of polycyclic imide intermediates *via* enolate substitution and Grignard addition, along with a key bridge-forming step involving a new method for sulfenylative radical cyclisation. The NMR data for synthetic concavine demonstrate that the original data reported for the natural product refer to the derived acetic acid salt, probably formed as an artefact of isolation or purification.

## Abstract 2

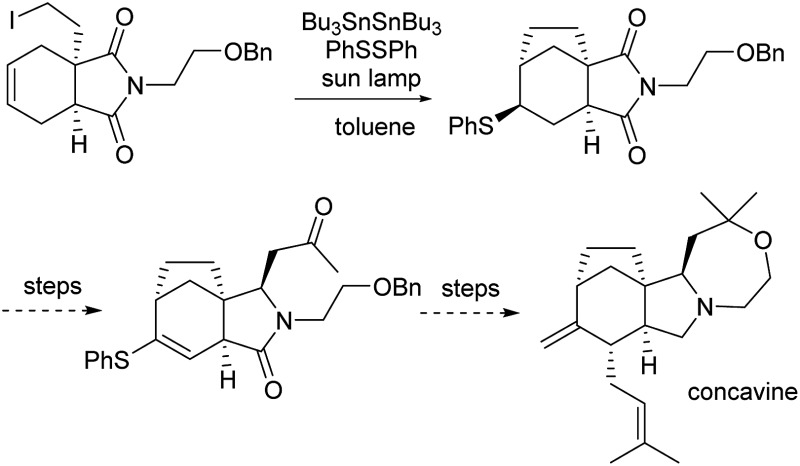
The synthesis of concavine is described, utilising a key sulfenylative radical cyclisation and highly regio- and stereocontrolled imide reactions.

## Introduction

**Fig. 1:**
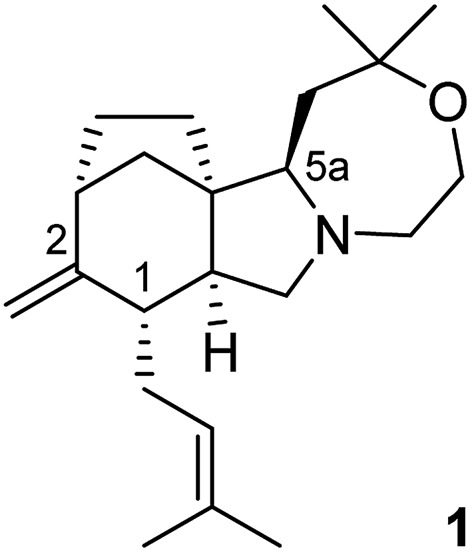
Structure of concavine.

In 2005 the group of Nasini reported the isolation of an alkaloid possessing a novel ring system from cultures of *Clitocybe concava* (Basidiomycetae).^1^ This new compound, called concavine, was assigned the structure **1** (Fig. 1) based on extensive NMR experiments.

The compound is an example of an abnormal diterpene alkaloid that incorporates an additional two carbons in the form of an internalised, serine-derived, ethanolamine fragment. This type of biosynthetic genesis has been explored in more detail for the related atisine-type alkaloids.^2^ Concavine features a tetracyclic core structure that incorporates a bicyclo[3.2.1]octane motif fused to a pyrrolo-oxazepane, and this unique polycyclic array, including the presence of five stereogenic centres, make concavine an interesting synthetic prospect.^3^

**Scheme 1:**
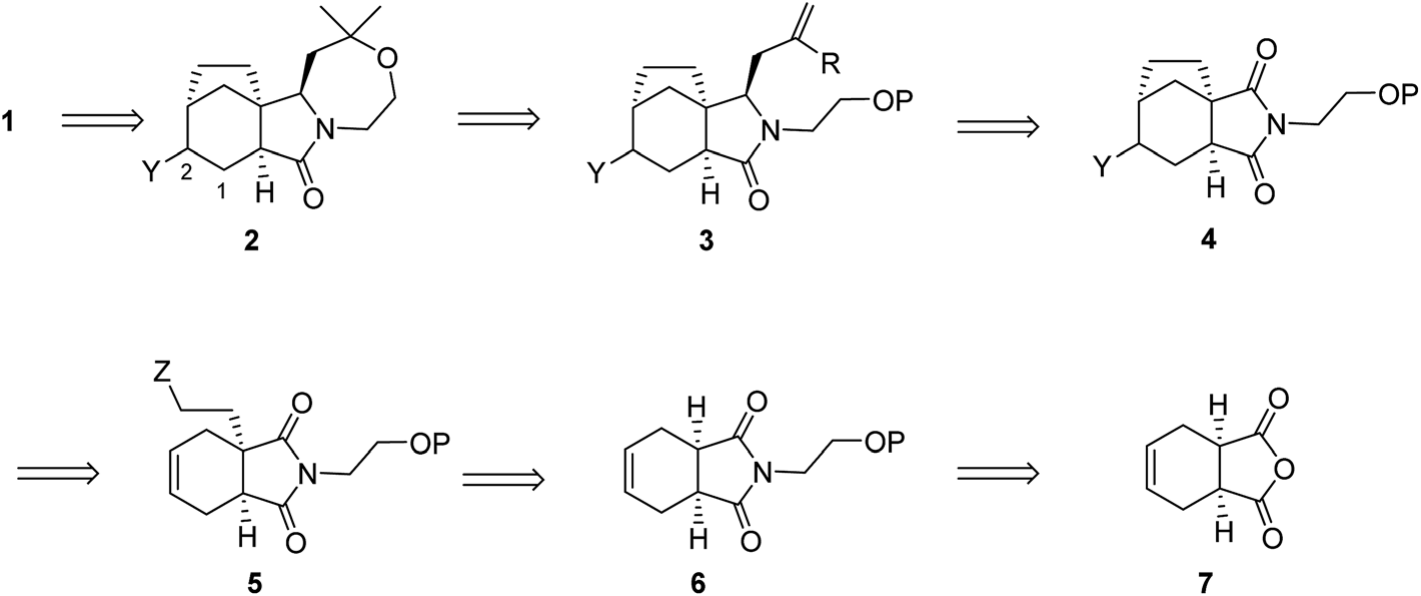
Retrosynthetic analysis of concavine.

Our retrosynthetic analysis of concavine is summarized in Scheme 1.

Concavine was envisaged to evolve from methylenation of the corresponding C-2 ketone. A late intermediate **2** (with undefined functionality Y) would serve as a precursor to this ketone, which would also enable diastereoselective installation of the C-1 prenyl group by enolate alkylation. The oxazepane ring in **2** would be formed from an intermediate **3**, or equivalent, by closure of an ether linkage. Access to intermediate lactam **3** would require regio- and stereoselective manipulation of a precursor imide **4**, so as to enable selective allylation of the more highly substituted imide C=O function. Stereocontrol in the introduction of an appropriate allyl substituent, most likely allyl (R = H) or methallyl (R = Me) would be possible *via* appropriate ordering of allylation and reduction events, the second of which would involve a reactive *N*-acyliminium intermediate.

Formation of the bicyclo[3.2.1]octane subunit by a C–C bond forming event was envisaged from a precursor **5**, equipped with an appropriate functionality Z. At the outset the identity of the functions Y and Z and the precise ordering of various events were not clear, although a number of options presented themselves.

In any case, enolate substitution of an appropriate imide (**6**), derived from commercial anhydride **7**, would provide imide **5**, which would need to incorporate functionality (Z) to enable subsequent two-carbon bridge formation. It is worth noting that any route planned around an initial imide desymmetrization might offer opportunities for an asymmetric variant, for example by our well-proven chiral lithium amide base methodology.^4^ However, at the outset we opted for an initial approach to the alkaloid in racemic form.

## Results and discussion

**Scheme 2:**
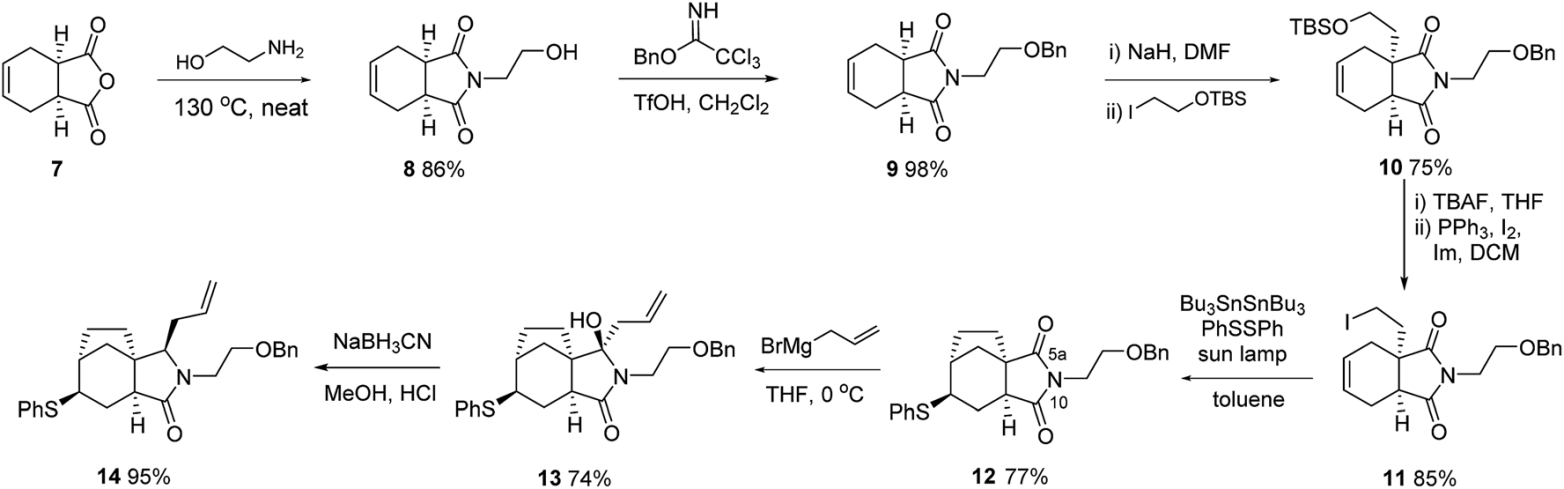
Initial phase of synthesis of concavine.

This plan required us to solve a number of key selectivity issues, including the identities of various groups and functions that would ultimately allow access to concavine. The initial phase of our synthesis is shown in Scheme 2.

Preparation of multigram quantities of a suitable starting imide **9** was trivial, starting with anhydride **7** and installing a suitably protected *N*-hydroxyethyl fragment. Our first explorations of imide enolate substitution using **9** centred on the use of activated electrophiles, particularly various bromoacetates. However, the derived imide esters proved problematic substrates in subsequent allylation chemistry and we instead made use of a suitably protected iodoethanol fragment,^5^ which provided imide **10** in respectable yield.

Deprotection and iodide formation under standard conditions proceeded smoothly to give **11**. The closure of the two carbon bridge onto the cyclohexene ring was required to proceed with concomitant functionalisation (group Y in our initial analysis). Initial attempts focused on an oxidative radical cyclisation that would provide an alcohol intermediate **4** (Y = OH). We applied protocols described by the groups of Nakamura and Prandi,^6,7^ which utilise combinations of Bu_3_SnH and oxygen, to iodide **11** but were not able to achieve productive cyclisation. Instead, we turned to a range of organometallic methods, probably involving cyclisation of radical intermediates, involving the use of either EtMgBr or SmI_2_, with electrophilic trapping.^8,9^ Although in some cases we saw evidence of C–C bond formation we could not trap the cyclised organometallic with electrophiles such as PhSSPh. Instead, we developed a variant of the procedure described by Renaud for radical cyclization with trapping by benzenesulfonyl azide to give azides.^10^ By employing a mixture of hexabutylditin and PhSSPh, and irradiating with a sun lamp, we were able to cleanly convert iodide **11** into the cyclised sulfide **12**, isolated as a single diastereoisomer, in acceptable yield. Attempts to install oxygen in place of the sulfur substituent, by substituting TEMPO for PhSSPh in this type of process were not fruitful.^11^

**Fig. 2:**
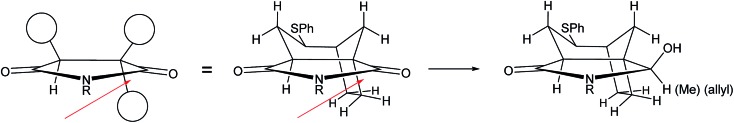
Mode of regio- and stereocontrolled addition to imide **12**.

At this stage we required to establish the regio- and stereocontrolled additions to the imide function in which reaction of the more substituted position (C-5a, concavine numbering) would occur in preference to addition at C-10. This type of control would be expected based on models of imide reduction developed by Speckamp, which included consideration of Bürgi–Dunitz type trajectories for the nucleophilic attack.^12^ Gratifyingly, addition of Grignard reagents, including allylmagnesium bromide and MeMgBr provided this mode of regioselectivity and it became evident that the facial bias of the tricyclic system present in **12** provided for very high levels of diastereocontrol, with nucleophiles attacking from the more exposed convex face with respect to the original bicyclic imide motif, Fig. 2.

**Fig. 3:**
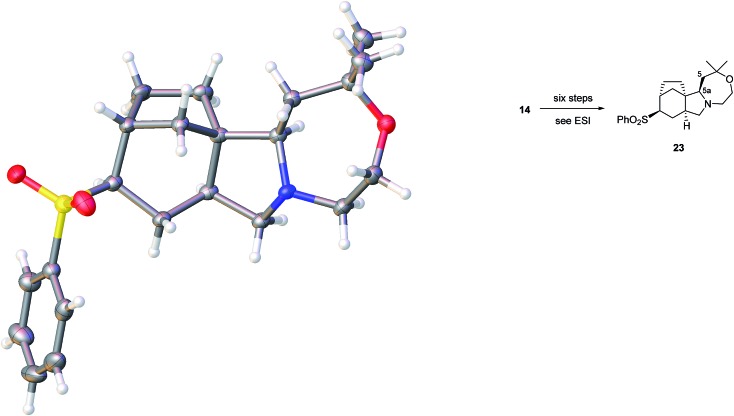
Crystal structure of **23** with ellipsoids drawn at the 50% probability level. The structure contains one molecule of dichloromethane per molecule of **23** which has been omitted for clarity.

We believe that the conformation of the cyclohexane ring is boat-like (see crystal structure of related sulfone in Fig. 3) and that the overall topology and substitution pattern effectively block competing pathways as illustrated in Fig. 2. This mode of reaction was also observed in NaBH_4_ reduction – see later.

In the case of allylation, this overall pattern of selectivity allowed for efficient conversion of **12** into **13**, with only minor amounts (*ca.* 7%) of an alternative regioisomeric adduct being observed. Subsequent controlled reduction was initially attempted using BF_3_–OEt_2_ in combination with silanes such as Et_3_SiH or Ph_3_SiH.^13^ However, these reagents resulted in facile elimination of water and formation of a dienamine product. After some experimentation we established that the use of NaBH_3_CN under Brønsted acidic conditions provided excellent results,^14^ the stereocontrol following the pattern observed previously, so as to convert **13** into **14** with inversion of the orientation of the allyl substituent.

**Scheme 3:**
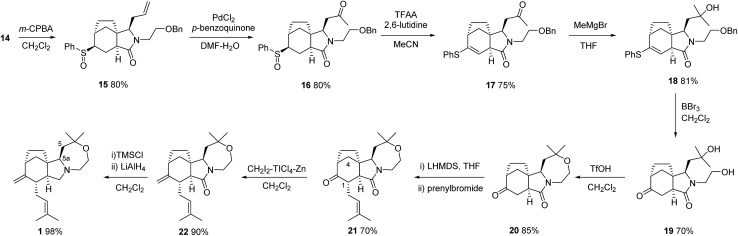
Completion of the concavine synthesis.

With the carbocyclic skeleton of concavine complete the remaining tasks included: (i) formation of the oxazepane ring, (ii) conversion of the sulfide function into a ketone and, (iii) installation of the remaining prenyl substituent. The successful sequence that emerged, leading to the complete structure of concavine, is shown in Scheme 3.

Preliminary experiments probed the viability of either an oxidative desulphonylation of a sulfone corresponding to sulfide **14** (or a later intermediate lacking the amide function), or a Pummerer-type process applied to an appropriate sulfoxide. Although an intermediate sulfone was prepared from **14**, we were unable to effect oxidative desulfonylation by applying a number of published protocols.^15^ We chose instead to progress sulfoxide intermediate **15**, which was easily prepared as an inconsequential 1 : 1 mixture of diastereoisomers at the sulfur centre.

Our initial projection for the next steps involved allyl chain modification and formation of the oxazepane prior to a Pummerer ketone synthesis. This sequence was disrupted by the observation that removal of the benzyl ether protection was problematic in the presence of a sulfoxide. To our surprise, we also observed that this system provided vinyl thioethers, and not ketones, on exposure to Pummerer conditions.^16^ These observations led us to an unplanned order of steps, starting with the application of classical Wacker conditions to **15** to give the desired ketone **16** in good yield.^17^ Treatment of this compound with the aforementioned Pummerer conditions gave vinyl thioether **17** (and not a problematic diketone), and subsequent reaction with MeMgBr then gave tertiary alcohol **18**.

Progression of this intermediate proved highly problematic, since removal of the benzyl ether protection failed under conditions used on simpler models (*e.g.* TMSI or H_2_, Pd(OH)_2_), and the vinyl thioether proved resistant to conventional hydrolysis conditions (TiCl_4_, CH_2_Cl_2aq_; HClO_4_; Fe(NO_3_)_3_-bentonite K-10).^18–20^ After considerable experimentation, and to our delight, we found that exposure of sulfide **18** to BBr_3_ effected both the desired deprotection and the vinyl sulfide hydrolysis in a convenient one-pot process, providing ketone **19** in 70% yield.^21^

With this key hurdle overcome, the key ring closure was found to be possible using very strongly acidic conditions, to provide tetracyclic oxazepane **20**.

In all of the stereoselective steps explored on this framework we had observed high levels of selectivity corresponding to reagent attack on the exposed convex face with respect to the initial bicyclic imide framework. This mode of reactivity was observed again in the regio- and stereoselective prenylation of ketolactam **20** using LHMDS and prenyl bromide. Thus, overall topological control from the azabicyclo[4.3.0] motif dominates and provides the desired product **21**, despite the fact that this corresponds to *endo*-alkylation with respect to the bicyclo[3.2.1] ketone fragment.^22^ Evidence for the stereochemical outcome included an nOe correlation between the remaining methine at C-1 and one of the hydrogens in the one-carbon bridge at C-4.

The remaining steps involving ketone methylenation to provide **22** and lactam reduction were high yielding and provided a final product anticipated to correspond with the natural product concavine (**1**). However, comparison of the ^1^H and ^13^C NMR data of our synthetic sample of **1** with the published data (in two solvents) revealed some significant discrepancies. In the ^1^H NMR spectra the differences were clustered around the pyrrolidine nitrogen, for example the NC*H* methine at C-5a appeared at 0.42 (acetone-d_6_) to 0.56 (CDCl_3_) ppm upfield from the reported shift values. Shift deviations were also observed for the C-5 CH_2_ of up to 0.55 ppm in the ^1^H NMR spectra and up to 2.60 ppm in the ^13^C spectra.

The identity of our synthetic series, including the relative stereochemistry, appeared secure, based on extensive spectroscopic characterisation. In addition, we had supplies of sulfones from the abortive oxidative desulfonylation approach mentioned above, and one such intermediate **23**, prepared in a six-step sequence from sulfide **14** provided crystals suitable for X-ray crystallographic analysis, Fig. 3.^23^

The structure is completely in accord with expectations, including the completed oxazepane ring and the correct relative configuration at C-5a. Interestingly, the sulfone substituent is equatorially orientated on a cyclohexane that adopts a boat conformation.

Although the extensive NMR data reported by Nasini and co-workers appeared consistent with our own, the chemical shift differences shown by our synthetic material, and centred around the pyrrolidine ring, were too substantial to ignore. Cognisant of the possible p*K*_a_ sensitivity of a basic natural product, we were aware of reports that HCl salt formation had resolved downfield shifts in other alkaloids, for example in the Nishida synthesis of Nakadomarin A, the spectra of the synthetic material were reconciled with those described for the natural product only after formation of an alkaloid-2HCl salt.^24^ Unfortunately, in our case, formation of HCl salts of **1**, resulted in excessive downfield shifting of a number of signals with the result that the overall correlation of both ^1^H and ^13^C NMR spectra was worse than for our neutral concavine sample.

**Scheme 4:**
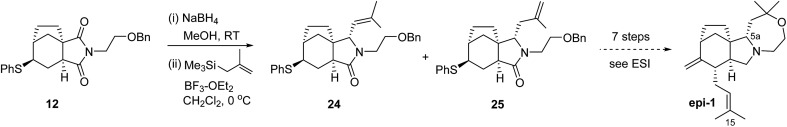
Synthesis of concavine **5a** epimer, epi-**1**.

With the apparent problem seemingly centred on the pyrrolidine region, we speculated that the natural product could be the C-5a epimer. We were able to make this compound through minor modifications to our route, starting with the sulfenyl imide **12**, Scheme 4.

By simply switching the ordering of allylation and reduction steps applied to **12**, relative to Scheme 2, we were able to prepare a mixture of **24** and **25**, each with the desired epimeric (compared to **14**) configuration at C-5a. Compound **25** was transformed uneventfully through an unoptimised 7 step sequence parallel to that employed for concavine.

To our chagrin, this synthetic effort generated an epimeric alkaloid epi-**1** with NMR spectral data that deviated even more significantly from those originally published – in excess of a 15 ppm shift for C-15 in the ^13^C NMR for example.

Fortunately, at this stage, a member of the Italian group responsible for isolating concavine was able to locate an original sample and forward it to us.^25^ Preliminary examination of this sample by NMR showed close correspondence with the published data, although the sample showed signs of contamination or decomposition. Remarkably, passing this sample through a short silica column gave a clean alkaloid the data for which were a complete match to our synthetic material!

Further examination of the spectra from the initially received sample from Milan provided an explanation for this phenomenon, since there was evidence of acetate (AcO^–^) signals in the spectra, including a methyl signal at *δ* = 1.98 ppm in the ^1^H spectrum and at *δ* = 22.8 in the ^13^C spectrum. Titration of our synthetic sample with HOAc then gave ^1^H and ^13^C spectra in CDCl_3_, which clearly demonstrated that the compound is identical to the reported structure. Thus, the reported data appear to be for a concavine-HOAc salt, presumably generated as an artefact of purification and some signals in the spectra were overlooked (perhaps discounted as solvent).

Interestingly, the original isolation paper describes purification of concavine with rather polar eluants containing 5–10% MeOH. In our hands the use of EtOAc–petroleum ether mixtures was adequate to effect alkaloid purification, and it appears that any salts (HCl or HOAc) are decomposed to give the neutral amine when purification is carried out this way.

In conclusion, we have described the first synthesis of the unusual alkaloid concavine (**1**), using a stereocontrolled route that is flexible enough to accommodate the synthesis of an epimer (epi-**1**). The route features a new sulfenative radical cyclisation protocol, an unexpected vinyl sulfide synthesis and a fortuitous hydrolysis of said vinyl sulfide, concomitant with an ether cleavage, using BBr_3_. Importantly, we have clarified that the reported data for concavine actually relate to a derived acetic acid salt.
