# Supplementary material for: First total synthesis of concavine
Source: Chem Sci. 2017 Feb 24;8(5):3384–9. doi: 10.1039/c6sc05627j (PMC5416908; doi:10.1039/c6sc05627j)

Electronic Supplementary Material (ESI) for Chemical Science.
This journal is © The Royal Society of Chemistry 2017

Supporting information

First Total Synthesis of Concavine

François Saint-Dizier,[a] and Nigel S. Simpkins*[a]

[a]School of Chemistry, University of Birmingham, Edgbaston, Birmingham, B15 2TT, UK

General Methods 2
Experimental data 3
Synthesis of concavine from **7** 3
Synthesis of **epi-1** from **12** 13
Synthesis of sulfone **23** from **14** 18
Formation of **1-HCl** and **1-AcOH** from concavine **1** 22
Comparison between synthesised and reported concavine 24
NMR spectra 30

General Methods

Unless stated, all reactions were carried out in oven-dried glassware under a nitrogen atmosphere. Anhydrous THF, MeOH, MeCN, Et_2O and CH_2Cl_2 were collected from a PureSolv™ solvent purification system. All other solvents and reagents were used as received from commercial suppliers unless otherwise stated.

NMR data were recorded on a Bruker AVIII300 (^{1}H = 300 MHz, T = 295.0 K) or AVIII400 (^{1}H = 400 MHz, ^{13}C = 101 MHz, T = 294.3 K) spectrometer. Spectra were recorded in chloroform-*d* or acetone-d_6 and calibrated on the solvent signal. For spectra in chloroform-*d*, δ_H = 7.26 ppm and δ_C = 77.16 ppm were used as references. For spectra in acetone-d_6, δ_H = 2.05 ppm and δ_C = 29.84 ppm were used as references The following abbreviations are used for multiplicity in ^{1}H NMR spectra: s (singlet), d (doublet), t (triplet), q (quartet), m (multiplet), br (broad), app (apparent). Proton-decoupled ^{13}C NMR spectra were recorded using the DEPTq pulse sequence and/or the UDEFT pulse sequence. Chemical shifts (δ) are quoted in ppm and coupling constants (*J*) are shown in Hz to one decimal place.

Reaction progress was monitored by thin-layer chromatography (TLC) performed on Merck silica gel 60 F_{254} plates, which were visualised under UV light (254 nm) and potassium permanganate dip. Flash column chromatography was carried out using silica (Geduran Si 60, 40-63 μm, VWR) and the indicated solvent systems.

Infrared spectra were recorded on a Perkin-Elmer Spectrum 100 FTIR spectrometer. Wavelengths (ν) are reported in cm^{-1}. EI mass spectra were recorded on a VG ZabSpec magnetic sector mass spectrometer and ESI mass spectra were recorded on a Micromass LCT time of flight mass spectrometer by the analytical facilities at the University of Birmingham. Melting points were measured with a Gallenkamp melting point apparatus with an open tube and are uncorrected.

The sun lamp characteristics are "Model:SA122, ta 40C / 220-240V ~50Hz / QT-DE12 R7s MAX 500W".

Experimental data

Synthesis of concavine from 7

Preparation of imide **8**[1]

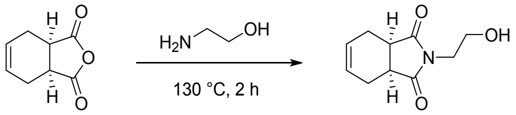

7 **8**

Phthalic anhydride **7** (10.00 g, 65.78 mmol, 1.0 eq.) dissolved in ethanolamine (3.90 mL, 65.78 mmol, 1.0 eq.) was heated at 130 °C for 2 h. After being cooled to RT, the mixture was washed with an aqueous solution of HCl (1 M, 40 mL). The organic layer was dried over $MgSO_4$, filtered and concentrated under reduced pressure. The crude product was purified by recrystallisation (Et_2O) to furnish imide **8** (11.03 g, 86%) as a white powder. **m.p.** 63 – 64 °C (lit.[1] 68 – 70 °C); **IR** ν_{max}/cm^{-1} 3452, 3042, 2950, 1689, 1400, 1166, 697; **^{1}H NMR** (400 MHz, $CDCl_3$) δ 5.99 – 5.82 (m, 2H), 3.80 – 3.62 (m, 4H), 3.22 – 3.04 (m, 2H), 2.67 – 2.52 (m, 2H) 2.33 – 2.15 (m, 2H); **^{13}C NMR** ($CDCl_3$, 101 MHz) δ 180.9, 127.9, 60.9, 42.1, 39.2, 23.7; **HRMS** (ES) found 196.0897 [MH]$^+$, requires 196.0895 for $C_{10}H_{13}NO_3$.

Protection of alcohol **9**

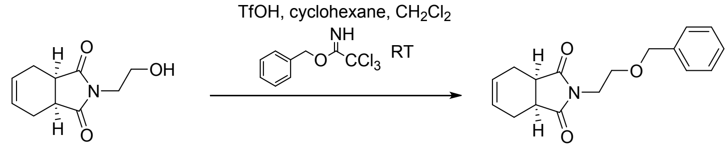

8 **9**

Triflic acid (27 µL, 0.31 mmol, 0.2 eq.) was added to a solution of benzyl trichloroacetimidate (0.57 mL, 3.07 mmol, 2.0 eq.) and **8** (300 mg, 1.54 mmol, 1.0 eq.) in a mixture of CH_2Cl_2 (1 mL) and cyclohexane (2 mL) at 0 °C. After 0.5 h the reaction mixture was warmed to RT and stirred for 2 h before being quenched with water. The aqueous layer was extracted with EtOAc (3 × 5 mL), the combined organic layers were washed with a saturated aqueous solution of $NaHCO_3$ (10 mL), dried over $MgSO_4$, filtered and concentrated under reduced pressure. The crude mixture was purified by flash column chromatography (gradient: hexane/EtOAc = 4/1 to 2/1) to give benzyl ether **9** (430 mg,

[1] Loddo, R. and co-workers, *J. Med.Chem.* **2005**, *48* (11), 3858-3873

98%) as a colourless oil. **IR** ν_{max}/cm^{-1} 3039, 2949, 2855, 1698, 1399; **^{1}H NMR** (400 MHz, $CDCl_3$) δ 7.40 – 7.25 (m, 5H), 5.90 – 5.80 (m, 2H), 4.52 (s, 2H), 3.78 – 3.71 (m, 2H), 3.65 – 3.60 (m, 2H), 3.10 – 3.05 (m, 2H), 2.61 – 2.57 (m, 2H), 2.29 – 2.20 (m, 2H); **^{13}C NMR** (101 MHz, $CDCl_3$) δ 180.0, 138.0, 130.9, 128.4, 128.3, 127.7, 72.6, 66.2, 39.0, 38.3, 23.5; **HRMS** (ES) found 308.1270 [MNa]$^+$, requires 308.1263 for $C_{17}H_{19}NO_3Na$.

Preparation of TBS-protected alcohol **10**

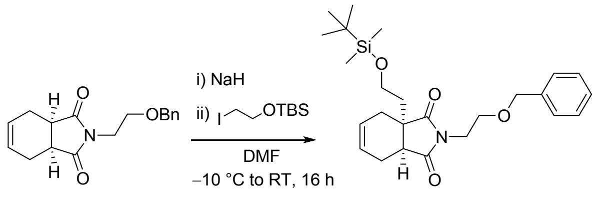

9 **10**

NaH (60% in mineral oil, 326 mg, 8.15 mmol, 1.4 eq.) was added to a solution of **9** (1.66 g, 5.82 mmol, 1.0 eq.) in dry DMF (15 mL) at –10 °C. The mixture was stirred for 20 min at −10 °C then TBS-protected iodoethanol (5.00 g, 17.46 mmol, 3.0 eq.) was added. The solution was allowed to warm to RT and stirred overnight before being quenched with a saturated aqueous solution of NH_4Cl. The aqueous layer was extracted with EtOAc (3 × 20 mL), the combined organic layers were washed with water (4 × 10 mL), dried over $MgSO_4$, filtered and concentrated under reduced pressure. The crude mixture was purified by flash column chromatography (gradient: hexane/EtOAc = 10/1 to 7/1) to furnish TBS-protected alcohol **10** (1.93 g, 75%) as a colourless oil. **IR** ν_{max}/cm^{-1} 3030, 2952, 2929, 2856, 1774, 1699, 1398, 1337, 1255, 1104, 835; **^{1}H NMR** (400 MHz, $CDCl_3$) δ 7.37 – 7.24 (m, 5H), 5.88 – 5.76 (m, 2H), 4.49 (s, 2H), 3.78 – 3.67 (m, 4H), 3.60 – 3.55 (m, 2H), 3.19 (dd, *J* = 7.3, 2.4 Hz, 1H), 2.66 (ddd, *J* = 15.6, 6.0, 2.4 Hz, 1H), 2.50 (dd, *J* = 15.1, 6.0 Hz, 1H), 2.21 (ddd, *J* = 15.6, 7.3, 2.6 Hz, 1H), 2.05 – 1.96 (m, 2H), 1.87 (ddd, *J* = 14.2, 6.5, 5.4 Hz, 1H), 0.86 (s, 9H), 0.01 (s, 3H), 0.00 (s, 3H); **^{13}C NMR** (101 MHz, $CDCl_3$) δ 182.3, 179.7, 138.0, 128.3, 127.9, 127.5, 127.3, 72.6, 66.3, 59.6, 47.2, 44.4, 39.1, 38.2, 31.4, 25.9, 24.0, 18.2, -5.6; 1 signal in the C-H aromatic region was not observed due to overlap; **HRMS** (ES) found 444.2576 [MH]$^+$, requires 444.2570 for $C_{25}H_{38}NO_4Si$.

Preparation of iodide **11**

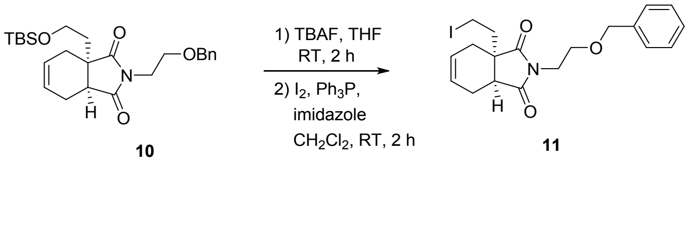

10 **11**

A solution of TBAF (1.0 M in THF, 13.50 mL, 13.50 mmol, 1.5 eq.) was added to a solution of **10** (3.98 g, 8.98 mmol, 1.0 eq.) in dry THF (20 mL) at RT. The reaction mixture was stirred for 2 h at RT, then quenched with a saturated aqueous solution of NH_4Cl. The aqueous layer was extracted with EtOAc (3 × 15 mL), the combined organic layers were washed with brine (20 mL), dried over $MgSO_4$, filtered and concentrated under reduced pressure. The crude mixture was dissolved in dry CH_2Cl_2 (20 mL) and added to solution of iodine (3.07 g, 12.15 mmol, 1.35 eq.), PPh_3 (2.83 g, 10.81 mmol, 1.25 eq.) and imidazole (918 mg, 13.5 mmol, 1.50 eq.) in dry CH_2Cl_2 (20 mL) at RT. The mixture was stirred for 2 h at RT then concentrated under reduced pressure. The crude mixture was purified by flash column chromatography (gradient: petrol/EtOAc = 4/1 to 2/1) to furnish iodide **11** (3.33 g, 85% after 2 steps) as a pale orange oil. **IR** v_{max}/cm^{-1} 3037, 2954, 2861, 1774, 1695, 1433, 1397, 1337, 1186, 1103, 1041; **^{1}H NMR** (400 MHz, $CDCl_3$) δ 7.39 – 7.23 (m, 5H), 5.90 – 5.76 (m, 2H), 4.47 (s, 2H), 3.79 – 3.67 (m, 2H), 3.65 – 3.55 (m, 2H), 3.07 (m, 2H), 2.80 (dd, *J* = 7.2, 2.4 Hz, 1H), 2.73 – 2.63 (m, 1H), 2.56 (dd, *J* = 15.5, 6.3 Hz, 1H), 2.38 (ddd, *J* = 13.9, 12.1, 5.2 Hz, 1H), 2.29 – 2.21 (m, 1H), 2.21 – 2.14 (m, 1H), 1.94 (dd, *J* = 15.5, 2.6 Hz, 1H); **^{13}C NMR** (101 MHz, $CDCl_3$) δ 180.7, 178.6, 137.8, 128.4, 128.0, 127.7, 127.6, 127.3, 72.7, 66.0, 49.9, 44.2, 42.3, 38.4, 30.5, 23.9, -3.4; **HRMS** (ES) found 440.0714 [MH]$^+$, requires 440.0723 for $C_{19}H_{23}NO_3I$.

Preparation of phenyl sulfide **12**

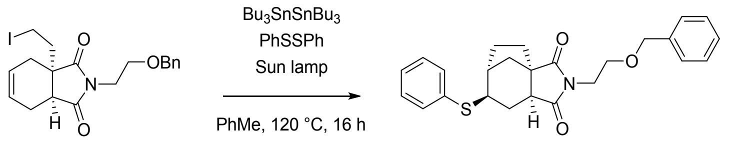

11 **12**

Hexabutylditin (2.43 mL, 4.83 mmol, 1.5 eq.) was added to a solution of **11** (1.41 g, 3.22 mmol, 1.0 eq.) and diphenyl disulfide (1.35 g, 4.83 mmol, 1.5 eq.) in dry PhMe (16 mL) at RT. The reaction mixture was irradiated with a sun lamp for 2 h. The sun lamp was then switched off and the mixture was heated to 120 °C and stirred for 16 h. The reaction mixture was cooled to RT and the solvent was removed under reduced pressure. The crude mixture was purified by flash column chromatography (gradient: hexane/EtOAc = 8/1 to 4/1) to give phenyl sulfide **12** (1.04 g, 77%) as a colourless oil. **IR** v_{max}/cm^{-1} 2954, 2872, 1771, 1700, 1392, 1101, 738; **^{1}H NMR** (400 MHz, $CDCl_3$) δ 7.32 – 7.10 (m, 10H), 4.41 (s, 2H), 3.73 – 3.63 (m, 2H), 3.59 – 3.51 (m, 2H), 3.06 (dd, *J* = 11.5, 6.6 Hz, 1H), 2.39 – 2.30 (m, 2H, H-2), 2.27 – 2.18 (m, 1H), 2.16 – 2.06 (m, 2H), 1.65 (dd, *J* = 11.9, 3.5 Hz, 1H), 1.55 – 1.47 (m, 2H), 1.47 – 1.36 (m, 1H), 1.31 – 1.20 (m, 1H); **^{13}C NMR** (101 MHz, $CDCl_3$) δ 180.1, 177.9, 137.9, 134.6, 131.5, 129.1, 128.5, 127.8, 127.7, 127.1, 72.6, 66.0, 50.0, 49.7, 47.3, 40.2, 38.1, 33.9, 33.4, 32.0, 25.1; **HRMS** (ES) found 444.1601 [MNa]$^+$, requires 444.1609 for $C_{25}H_{27}NO_3NaS$.

Preparation of hemiaminal **13**

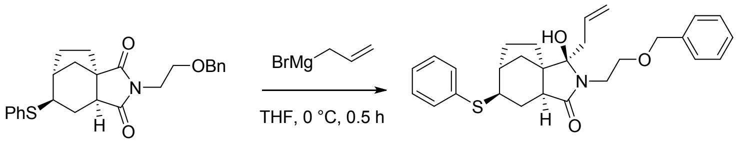

A solution of allylmagnesium bromide (1.0 M in Et_2O, 0.61 mL, 0.61 mmol, 1.3 eq.) was added to a solution of **12** (200 mg, 0.47 mmol, 1.0 eq.) in dry THF (4 mL) at 0 °C. The mixture was stirred for 0.5 h at 0 °C and then quenched with water. The aqueous layer was extracted with EtOAc (3 × 10 mL), the combined organic layers were dried over $MgSO_4$, filtered and concentrated under reduced pressure. The crude mixture was purified by flash column chromatography (gradient: hexane/EtOAc = 4/1 to 3/1) to furnish allyl **13** (163 mg, 74%) as a colourless oil. **IR** v_{max}/cm^{-1} 3387, 2938, 1687, 1438, 1091, 743; **^{1}H NMR** (400 MHz, $CDCl_3$) δ 7.40 – 7.09 (m, 10H), 5.83 – 5.69 (m, 1H), 5.04 – 4.96 (m, 2H), 4.49 (s, 2H), 4.16 (s, 1H, OH), 3.78 – 3.71 (m, 1H), 3.71 – 3.64 (m, 1H), 3.48 (ddd, J = 9.2, 3.4, 1.7 Hz, 1H), 3.28 – 3.18 (m, 2H), 2.38 – 2.33 (m, 2H), 2.33 – 2.26 (m, 1H), 2.25 – 2.18 (m, 1H), 2.07 – 1.97 (m, 2H), 1.96 – 1.91 (m, 1H), 1.75 – 1.68 (m, 1H), 1.60 (d, J = 11.8 Hz, 1H), 1.54 – 1.41 (m, 2H), 1.20 (dd, J = 11.8, 5.6 Hz, 1H); **^{13}C NMR** (101 MHz, $CDCl_3$) δ 174.1, 136.8, 135.6, 132.7, 132.0, 129.0, 128.8, 128.6, 128.2, 126.8, 119.2, 90.3, 74.0, 67.6, 55.4, 48.8, 46.7, 40.8, 39.9, 37.3, 30.0, 29.4, 28.7, 22.6; **HRMS** (ES) found 486.2078 $[MNa]^+$, requires 486.2079 for $C_{28}H_{33}NO_3NaS$.

Reduction of hemiaminal to give allyl lactam **14**

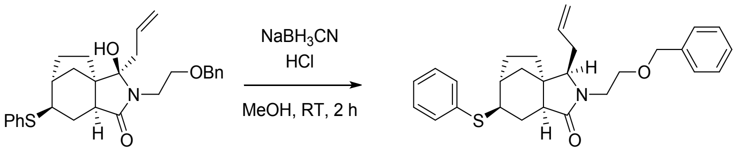

$NaBH_3CN$ (252 mg, 4.00 mmol, 2.0 eq.) was added to a solution of **13** (0.93 g, 2.00 mmol, 1.0 eq.) in methanol (10 mL) at 0 °C. Concentrated HCl (36%) was added until pH ≈ 3 and the reaction mixture was stirred for 1 h at 0 °C and 2 h at RT. The mixture was quenched by dropwise addition of a saturated aqueous solution of $NaHCO_3$. The aqueous layer was extracted with EtOAc (3 × 15 mL), the combined organic layers were dried over $MgSO_4$, filtered and concentrated under reduced pressure to give allyl **14** (853 mg, 95%) as a white oil. **IR** v_{max}/cm^{-1} 3057, 2935, 2865, 1680, 1438, 1275, 1099, 916, 739; **^{1}H NMR** (400 MHz, $CDCl_3$) δ 7.36 – 7.09 (m, 10H), 5.73 – 5.60 (m, 1H), 5.08 – 4.96 (m, 2H), 4.46 (d, J = 12.0 Hz, 1H), 4.40 (d, J = 12.0 Hz, 1H), 3.81 (dt, J = 14.5, 5.5 Hz, 1H), 3.60 (dd, J = 8.1, 4.7 Hz, 1H), 3.54 – 3.44 (m, 2H), 3.20 – 3.10 (m, 2H), 2.52 – 2.43 (m, 1H), 2.28 – 2.22 (m, 1H),

2.21 – 2.06 (m, 3H), 1.90 – 1.80 (m, 1H), 1.71 – 1.60 (m, 2H), 1.52 – 1.44 (m, 1H), 1.43 – 1.33 (m, 2H), 1.21 – 1.12 (m, 1H); **^{13}C NMR** (101 MHz, $CDCl_3$) δ 176.3, 138.1, 135.4, 134.9, 131.4, 128.9, 128.4, 127.7, 126.6, 117.7, 72.9, 67.8, 60.8, 49.8, 49.2, 48.2, 40.1, 38.7, 35.2, 33.7, 30.2, 27.9, 24.8; 1 signal in the CH aromatic region were not observed due to overlap; **HRMS** (ES) found 470.2134 $[MNa]^+$, requires 470.2130 for $C_{28}H_{33}NO_2NaS$.

Preparation of sulfoxide **15**

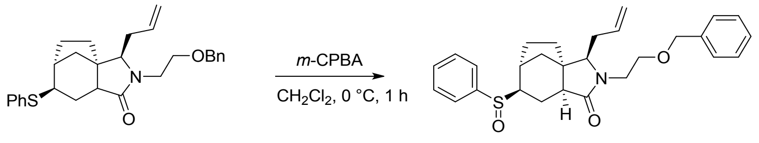

14 **15**

m-CPBA (77%, 795 mg, 3.56 mmol, 1.2 eq.) was added to a solution of **14** (1.22 g, 2.74 mmol, 1.0 eq.) in CH_2Cl_2 (15 mL) at 0 °C. The reaction mixture was stirred for 1.5 h then quenched with a saturated aqueous solution of $NaHCO_3$. The aqueous layer was extracted with CH_2Cl_2 (3 × 10 mL), the combined organic layers were dried over $MgSO_4$, filtered and concentrated under reduced pressure. The crude mixture was purified by flash column chromatography (gradient: hexane/EtOAc = 1/1 to 0/1) to yield sulfoxide **15** (1.01 g, 80%, 1.0:1.3 mixture of diastereoisomers) as a colourless oil. **IR** ν_{max}/cm^{-1} 3059, 2952, 2874, 1771, 1697, 1442, 1086, 1040, 748; **^{1}H NMR** (for both diastereoisomers, 400 MHz, $CDCl_3$) δ 7.75 – 7.14 (m, 20H), 5.77 – 5.55 (m, 2H), 5.13 – 4.94 (m, 4H), 4.47 – 4.36 (m, 4H), 3.86 – 3.70 (m, 2H), 3.66 – 3.61 (m, 2H), 3.53 – 3.46 (m, 4H), 3.20 – 3.10 (m, 2H), 2.92 – 2.87 (m, 1H), 2.79 – 2.74 (m, 1H), 2.53 – 2.40 (m, 4H), 2.26 – 2.11 (m, 2H), 2.11 – 2.02 (m, 2H), 2.02 –1.93 (m, 2H), 1.76 – 1.67 (m, 1H), 1.67 – 1.57 (m, 4H), 1.53 – 1.45 (m, 2H), 1.45 – 1.38 (m, 3H), 1.38 – 1.30 (m, 3H), 1.28 – 1.20 (m, 1H); **^{13}C NMR** (101 MHz, $CDCl_3$) δ 175.8, 175.7, 142.7, 141,4, 138.1, 137.4, 134.7, 131.4, 130.8, 129.2, 129.0, 128.4, 127.6, 125.6, 124.4, 117.8 (2C), 72.9 (2C), 67.8, 67.7, 65.1 (2C), 60.8, 60.6, 49.2, 48.7, 48.4, 47.1, 40.1, 36.2, 35.4, 34.4, 33.9, 33.5, 31.7, 31.6, 29.6, 28.7, 28.4, 18.0, 14.4; **HRMS** (ES) found 486.2082 $[MNa]^+$, requires 486.2079 for $C_{28}H_{33}NO_3NaS$.

Preparation of ketone **16**

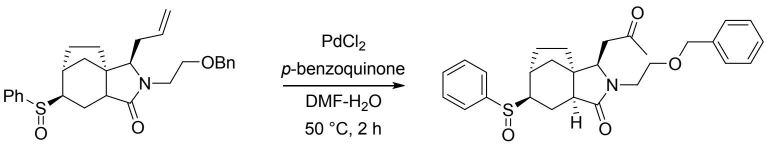

15 **16**

$PdCl_2$ (42 mg, 0.24 mmol, 0.15 eq.), *p*-benzoquinone (173 mg, 1.60 mmol, 1.0 eq.) were added to a solution of sulfoxide **15** (740 mg, 1.60 mmol, 1.0 eq.) in a mixture of DMF (7 mL) and H_2O (1 mL) at RT. The reaction mixture was stirred for 2 h at 50 °C before being quenched with an aqueous solution of 1 M HCl. The aqueous layer was extracted with EtOAc (3 × 15 mL), the combined organic layers were washed with water (5 × 10 mL), dried over $MgSO_4$, filtered and concentrated under reduced pressure. The crude mixture was purified by flash column chromatography (gradient: hexane/ EtOAc =1/1 to 0/1) to furnish ketone **16** (613 mg, 80%, 1.0:1.5 mixture of diastereoisomers) as a colourless oil. **IR** v_{max}/cm^{-1} 3062, 2945, 2871, 1714, 1676, 1442, 1093, 1037, 730; **^{1}H NMR** (for both diastereoisomers, 400 MHz, $CDCl_3$) 7.83 – 7.78 (m, 2H), 7.61 – 7.50 (m, 8H), 7.44 – 7.31 (m, 10H), 4.53 – 4.49 (m, 4H), 4.30 – 4.23 (m, 2H), 3.68 – 3.59 (m, 2H), 3.58 – 3.50 (m, 4H), 3.38 – 3.27 (m, 2H), 3.05 – 2.97 (m, 3H), 2.92 (dd, *J* = 7.8, 4.8 Hz, 1H), 2.74 – 2.68 (m, 1H), 2.55 – 2.50 (m, 1H), 2.49 – 2.44 (m, 2H), 2.28 – 2.21 (m, 1H), 2.20 – 2.11 (m, 2H), 2.09 (s, 3H), 2.05 (s, 3H), 1.86 – 1.67 (m, 6H), 1.64 – 1.40 (m, 7H), 1.20 (dd, *J* = 11.9, 5.3 Hz, 1H), 1.06 (dd, *J* = 11.6, 4.9 Hz, 1H); **^{13}C NMR** (101, $CDCl_3$) 206.9 (2C), 175.6 (2C), 142.7, 141.3, 137.9 (2C), 131.5, 130.8, 129.2, 129.1, 128.5, 128.0, 127.9, 127.8, 127.7, 125.6, 124.2, 73.4, 73.3, 68.4 (2C), 67.7, 65.1, 57.3, 57.0, 48.7, 48.1, 47.8, 46.3, 44.3, 43.2, 41.6, 41.5, 36.1, 35.8, 33.6, 31.7, 31.6, 30.2 (2C), 29.7, 29.5, 29.3, 18.1, 14.0; **HRMS** (ES) found 502.2023 $[MNa]^+$, requires 502.2028 for $C_{28}H_{33}NO_4SNa$.

Preparation of vinyl sulfide **17**

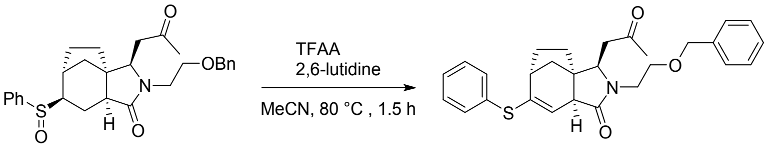

16 **17**

2,6-lutidine (0.25 mL, 2.12 mmol, 3.0 eq.) and TFAA (0.30 mL, 2.12 mmol, 3.0 eq.) were successively added to a solution of **16** (340 mg, 0.71 mmol, 1.0 eq.), in dry MeCN (2 mL) at RT. The mixture was heated to 80 °C and stirred for 1.5 h. After being cooled to RT, the solvent was removed

under reduced pressure and the crude mixture was purified by flash column chromatography (gradient: hexane/EtOAc = 3/1 to 1/1) to give vinyl sulfide **17** (245 mg, 75%) as an orange oil. **IR** v_{max}/cm^{-1} 3064, 3051, 2945, 2867, 1718, 1688, 1440, 1211, 1166, 745; **^{1}H NMR** (400 MHz, $CDCl_3$) δ 7.48 – 7.22 (m, 10H), 5.54 (d, J = 3.8 Hz, 1H), 4.49 (s, 2H), 4.28 (dd, J = 8.2, 3.0 Hz, 1H), 3.71 – 3.50 (m, 2H), 3.46 – 3.36 (m, 2H), 3.04 (dd, J = 18.0, 8.2 Hz, 1H), 2.88 (d, J = 3.8 Hz, 1H), 2.55 – 2.51 (m, 1H), 2.47 (dd, J = 18.0, 3.0 Hz, 1H), 2.04 (s, 3H), 1.98 – 1.86 (m, 2H), 1.74 – 1.59 (m, 2H), 1.53 (d, J = 10.9 Hz, 1H), 1.38 (dd, J = 10.9, 4.8 Hz, 1H); **^{13}C NMR** (101 MHz, $CDCl_3$) δ 207.4, 176.0, 146.1, 137.6, 132.6, 132.3, 129.2, 128.5, 128.2, 128.0, 127.9, 115.6, 73.5, 68.0, 56.9, 54.8, 48.6, 42.3, 42.1, 39.5, 34.4, 32.1, 31.6, 30.0; **HRMS** (ES) found 484.2034 [MNa]$^+$, requires 484.2031 for $C_{28}H_{31}NO_3NaS$.

Preparation of alcohol **18**

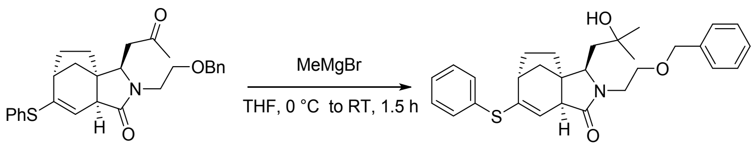

17 **18**

A solution of MeMgBr (3.0 M in Et_2O, 0.36 mL, 1.07 mmol, 1.5 eq.) was added to a solution of **17** (330 mg, 0.71 mmol, 1.0 eq.) in dry THF (5 mL) at 0 °C. The mixture was stirred for 1 h at 0 °C and 0.5 h at RT before being quenched with water. The aqueous layer was extracted with EtOAc (3 × 10 mL), the combined organic layers were washed with a saturated aqueous solution of NH_4Cl (2 × 10 mL), dried over $MgSO_4$, filtered and concentrated under reduced pressure. The crude mixture was purified by flash column chromatography (gradient: hexane/EtOAc = 2/1 to 1/1) to furnish alcohol **18** (274 mg, 81%) as a colourless oil. **IR** v_{max}/cm^{-1} 3430, 3068, 3055, 2951, 2868, 1670, 1090, 907, 726; **^{1}H NMR** (400 MHz, $CDCl_3$) δ 7.30 – 7.12 (m, 10H), 5.73 (d, J = 4.0 Hz, 1H), 4.41 (d, J = 11.3 Hz, 1H), 4.35 (d, J = 11.3 Hz, 1H), 3.74 (dd, J = 6.5, 1.7 Hz, 1H), 3.71 – 3.64 (m, 3H), 3.52 – 3.49 (m, 1H), 2.56 (d, J = 4.0 Hz, 1H), 2.41 – 2.38 (m, 1H), 1.87 – 1.71 (m, 2H), 1.65 (dd, J = 15.8, 6.5 Hz, 1H), 1.60 – 1.54 (m, 1H), 1.54 – 1.47 (m, 1H), 1.47 – 1.38 (m, 1H), 1.36 – 1.29 (m, 2H), 1.12 (s, 3H), 0.88 (s, 3H); **^{13}C NMR** (101 MHz, $CDCl_3$) δ 174.1, 144.1, 137.0, 133.5, 131.8, 129.1, 128.6, 128.5, 128.3, 127.2, 120.2, 73.9, 69.4, 69.2, 58.7, 55.5, 49.6, 41.1, 40.6, 39.7, 33.7, 32.2, 31.3, 31.1, 28.1; **HRMS** (ES) found 500.2231 [MNa]$^+$, requires 500.2235 for $C_{29}H_{35}NO_3NaS$.

Preparation of keto-diol **19**

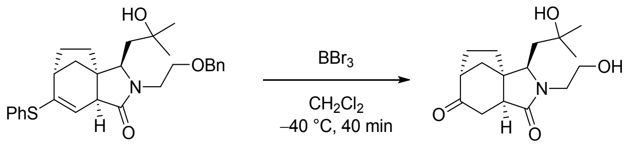

18 **19**

A solution of BBr_3 (1.0 M in CH_2Cl_2, 0.60 mL, 0.60 mmol, 2.5 eq.) was added to a solution of **18** (115 mg, 0.24 mmol, 1.0 eq.) in dry CH_2Cl_2 (3 mL) at −40 °C. The reaction mixture was stirred at −40 °C for 40 min, then quenched with water. The pH was adjusted to pH = 4-5 with an aqeuous solution of 2 N NaOH. The aqueous layer was extracted with EtOAc (5 × 10 mL), the combined organic layers were dried over $MgSO_4$, filtered and concentrated under reduced pressure. The crude mixture was purified by flash column chromatography (gradient: EtOAc/MeOH = 1/0 to 1/0.1) to yield ketone **19** (50 mg, 70%) as a colourless oil. **IR** ν_{max}/cm^{-1} 3391, 2963, 2875, 1713, 1666, 1417, 1067, 916, 730; **^{1}H NMR** (400 MHz, $CDCl_3$) δ 4.08 (dd, J = 5.9, 2.4 Hz, 1H), 3.83 – 3.61 (m, 3H), 3.59 (ddd, J = 15.8, 9.3, 3.9, 1H), 2.72 – 2.64 (m, 2H), 2.60 – 2.53 (m, 1H), 2.51 – 2.44 (m, 1H), 2.07 – 1.98 (m, 1H), 1.80 – 1.66 (m, 3H), 1.63 – 1.49 (m, 4H), 1.30 (s, 3H), 1.21 (s, 3H); **^{13}C NMR** (101 MHz, $CDCl_3$) δ 212.5, 176.4, 70.0, 62.0, 58.6, 50.4, 48.3, 46.6, 43.0, 40.6, 33.4, 31.9, 31.2, 30.2, 29.1, 26.4; **HRMS** (ES) found 318.1682 [MNa]$^+$, requires 318.1681 for $C_{16}H_{25}NO_4Na$.

Preparation of tetracycle **20**

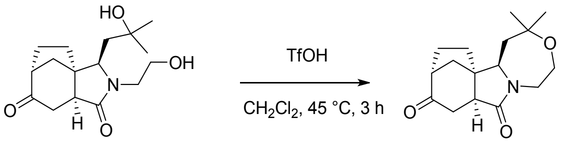

19 **20**

Triflic acid (60 µL, 0.67 mmol, 5.0 eq.) was added to a solution of **19** (40 mg, 0.13 mmol, 1.0 eq.) in dry CH_2Cl_2 (2 mL) at 0 °C. The reaction mixture was stirred at 0 °C for 15 min and 3 h at 45 °C. An aqueous solution of $NaHCO_3$ was added to quench the reaction. The aqueous layer was extracted with CH_2Cl_2 (3 × 8 mL), the combined organic layers were dried over $MgSO_4$, filtered and concentrated under reduced pressure to give tetracyclic compound **20** (32 mg, 85%) as a white powder. **m.p.** 182 – 184 °C; **IR** ν_{max}/cm^{-1} 2968, 2940, 2876, 1713, 1684, 1445, 1369, 1238, 1081; **^{1}H NMR** (400 MHz, $CDCl_3$) δ 3.85 (d, J = 10.8 Hz, 1H), 3.75 – 3.60 (m, 3H), 3.53 – 3.26 (m, 1H), 2.70 (dd, J = 8.4, 4.8

Hz, 1H), 2.58 (d, J = 8.5, 2H), 2.40 (t, J = 8.5 Hz, 1H), 2.08 – 1.98 (m, 1H), 1.78 – 1.63 (m, 4H), 1.62 – 1.52 (m, 2H), 1.45 (dd, J = 12.3, 4.8 Hz, 1H), 1.21 (s, 6H); **^{13}C NMR** (101 MHz, $CDCl_3$) δ 212.6, 175.2, 74.4, 60.2, 57,4, 48.5, 48.3, 46.1, 45.9, 41.1, 34.1, 32.3, 30.4, 28.8, 26.5, 25.9; **HRMS** (ES) found 300.1575 $[MNa]^+$, requires 300.1576 for $C_{16}H_{23}NO_3Na$.

Preparation of prenylated keto-lactam **21**

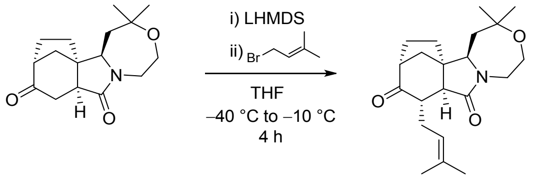

20 **21**

A solution of LHMDS (1.0 M in THF, 0.20 mmol, 0.20 mL, 1.1 eq.) was added to a solution of **20** (50 mg, 0.18 mmol, 1.0 eq.) in dry THF (2 mL) at −40 °C. After 15 min at −40 °C, prenyl bromide (62 µL, 0.54 mmol, 3.0 eq.) was added. The reaction mixture was stirred for 2 h at −40 °C, then allowed to warm to −10 °C and stirred for 2 h before being quenched with water. The aqueous layer was extracted with EtOAc (3 × 5 mL), the combined organic layers were dried over $MgSO_4$, filtered and concentrated under reduced pressure. The crude mixture was purified by flash column chromatography (gradient: hexane/EtOAc = 2/1 to 1/1) to yield prenylated compound **21** (43 mg, 70%) as a white powder. **m.p.** 125 − 127 °C; **IR** v_{max}/cm^{-1} 2966, 2944, 2874, 1710, 1687, 1416, 1226, 1100, 1080; **^{1}H NMR** (400 MHz, $CDCl_3$) δ 4.98 – 4.91 (m, 1H), 3.85 (d, J = 10.7 Hz, 1H), 3.79 – 3.67 (m, 3H), 3.39 – 3.32 (m, 1H), 2.78 – 2.72 (m, 2H), 2.69 – 2.62 (m, 1H), 2.54 – 2.45 (m, 1H), 2.12 (d, J = 7.6 Hz, 1H), 2.06 – 1.94 (m, 1H), 1.81 – 1.70 (m, 2H), 1.70 – 1.65 (m, 4H), 1.64 (s, 3H), 1.62 – 1.56 (m, 2H), 1.52 – 1.46 (m, 1H), 1.36 (dd, J = 12.1, 4.5 Hz, 1H), 1.27 (s, 6H); **^{13}C NMR** (101 MHz, $CDCl_3$) δ 214.2, 175.7, 135.2, 120.1, 74.4, 60.2, 57.1, 49.6, 48.5, 48.4, 45.9, 44.5, 41.1, 32.2, 30.0, 28.9, 27.7, 26.5, 25.9, 18.2; **HRMS** (ES) found 368.2191 $[MNa]^+$, requires 368.2202 for $C_{21}H_{31}NO_3Na$.

Preparation of diene (C-10 'oxa-concavine') **22**

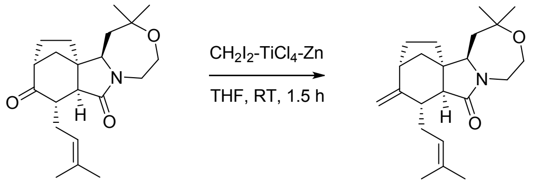

21 **22**

CH_2I_2 (0.40 mL, 5.00 mmol, 5.0 eq.) was added to a suspension of zinc (0.58 g, 9.00 mmol, 9.0 eq.) and $PbCl_2$ (15 mg, 0.05 mmol, 0.05 eq.) in dry THF (10 mL) at RT. After 0.5 h, $TiCl_4$ (1.0 M in CH_2Cl_2, 1 ml, 1 mmol, 1.0 eq.) was added at 0 °C and the mixture was stirred for 0.5 h at RT. A solution of [CH_2I_2-$TiCl_4$-Zn] (0.1 M in THF, 0.75 mL, 0.075 mmol, 1.3 eq.) was added to a solution of **21** (20 mg, 0.058 mmol, 1.0 eq.) at RT. After being stirred for 1.5 h, the mixture was diluted with EtOAc, the organic layer was washed with an aqueous solution of 1 M HCl, dried over $MgSO_4$, filtered and concentrated under reduced pressure. The crude mixture was purified by flash column chromatography (gradient: hexane/EtOAc = 2/1 to 1/1) to yield olefin **22** (18 mg, 90%) as a colourless oil. **IR** ν_{max}/cm^{-1} 3085, 2970, 2936, 2870, 1689, 1425, 1391, 1111, 1082; ^{1}**H NMR** (400 MHz, $CDCl_3$) δ 5.17 – 5.10 (m, 1H), 4.85 – 4.81 (m, 1H), 4.77 – 4.75 (m, 1H), 3.76 – 3.66 (m, 4H), 3.38 – 3.30 (m, 1H), 2.85 – 2.81 (m, 1H), 2.74 – 2.66 (m, 1H), 2.54 – 2.42 (m, 2H), 2.02 (d, *J* = 6.6 Hz, 1H), 1.97 – 1.88 (m, 1H), 1.76 – 1.71 (m, 1H), 1.70 (s, 3H), 1.64 (s, 3H), 1.62 – 1.54 (m, 3H), 1.53 – 1.46 (m, 1H), 1.40 (d, *J* = 11.4 Hz, 1H), 1.25 (s, 6H), 1.21 (dd, *J* = 11.4, 6.3 Hz, 1H); ^{13}**C NMR** (101 MHz, $CDCl_3$) δ 176.8, 154.7, 133.4, 122.0, 108.4, 74.5, 60.4, 57.5, 53.5, 48.7, 45.8, 42.5, 40.8, 35.4, 33.2, 31.8 (2C), 31.2, 29.0, 25.8 (2C), 18.3; **HRMS** (ES) found 366.2406 [MNa]$^+$, requires 366.2409 for $C_{22}H_{33}NO_2Na$.

Preparation of concavine **1**

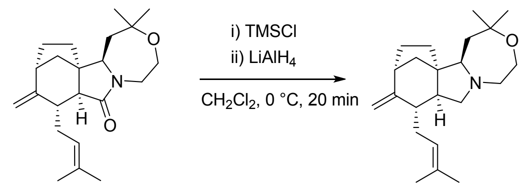

TMSCl (5 µL, 0.038 mmol, 1.2 eq.) was added to a solution of **22** (11 mg, 0.032 mmol, 1.0 eq.) in dry CH_2Cl_2 (1 mL) at 0 °C. The mixture was stirred for 15 min at 0 °C, then a solution of $LiAlH_4$ (2.4 M in THF, 20 µL, 0.048 mmol, 1.5 eq.) was added. After 20 min at 0 °C, the reaction mixture was quenched with an aqueous solution of 2 N NaOH. The aqueous layer was extracted with CH_2Cl_2 (3 × 3 mL), the combined organic layers were dried over $MgSO_4$, filtered and concentrated under reduced pressure. The crude mixture was purified by flash column chromatography (gradient: hexane/EtOAc = 1/1 to 0/1) to yield concavine **1** (10 mg, 98%) as a colourless oil. **IR** ν_{max}/cm^{-1} 3081, 2968, 2930, 2866, 2799, 1635, 1454, 1364, 1270, 1071, 884; ^{1}**H NMR** (400 MHz, $CDCl_3$) δ 5.14 – 5.07 (m, 1H), 4.82 – 4.79 (m, 1H), 4.74 – 4.72 (m, 1H), 3.81 (dd, *J* = 13.3, 11.1 Hz, 1H), 3.54 (ddd, *J* = 13.3, 3.2, 2.0 Hz, 1H), 3.02 – 2.92 (m, 2H), 2.80 (dd, *J* = 7.0, 4.4 Hz, 1H), 2.57 (d, *J* = 9.3 Hz, 1H), 2.50 app t, *J* = 10.0 Hz, 1H), 2.34 (ddd, *J* = 12.7, 10.8, 2.0 Hz, 1H), 2.22 – 2.15 (m, 2H), 2.15 – 2.10 (m, 1H), 1.98 – 1.87 (m, 1H), 1.79 – 1.70 (m, 2H), 1.68 (s, 3H), 1.61 (s, 3H), 1.57 (dd, *J* = 10.2, 3.7 Hz, 1H), 1.54 –

1.46 (m, 1H), 1.46 – 1.36 (m, 2H), 1.32 – 1.24 (m, 1H), 1.20 (s, 3H), 1.18 (s, 3H), 0.97 (dd, *J* = 11.3, 4.4 Hz, 1H); **^{13}C NMR** (101 MHz, $CDCl_3$) δ 157.1, 131.8, 123.0, 107.2, 74.8, 64.8, 62.0, 61.5, 58.6, 53.4, 49.6, 41.8, 41.7, 41.5, 33.5, 33.4, 30.4, 30.0, 28.8, 27.6, 25.8, 18.0; **HRMS** (ES) found 330.2799 $[MH]^+$, requires 330.2797 for $C_{22}H_{36}NO$.

Synthesis of ***epi-1*** *from* ***12***

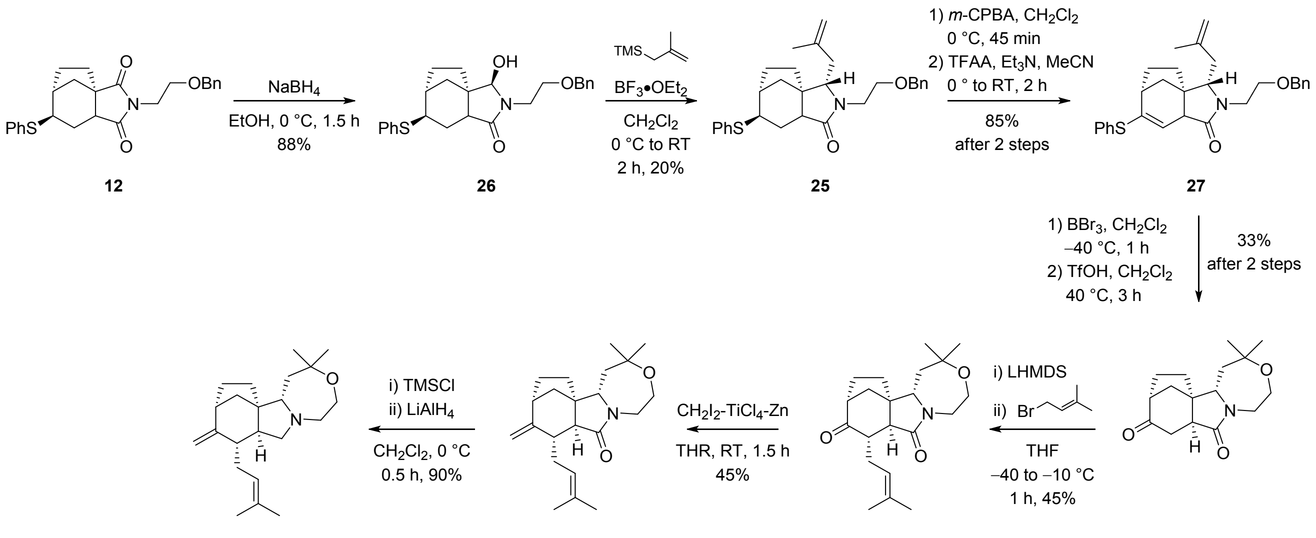

Preparation of hemiaminal **26**

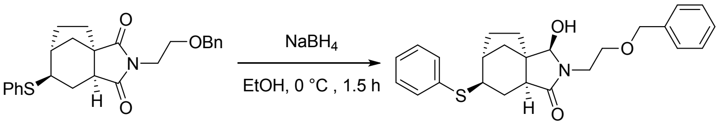

Sodium borohydride (277 mg, 7.36 mmol, 5.0 eq.) was added portionwise to a solution of **12** (620 mg, 1.47 mmol, 1.0 eq.) in ethanol (12 mL) at 0 °C. The mixture was stirred for 1.5 h at 0 °C then quenched with water. The aqueous layer was extracted with CH_2Cl_2 (3 × 10 mL), the combined organic layers were dried over $MgSO_4$, filtered and concentrated under reduced pressure to yield hemiaminal **26** (548 mg, 88%) as a colourless oil. **IR** v_{max}/cm^{-1} 3358, 2940, 2868, 1667, 1583, 1453, 1102, 1026, 739; **^{1}H NMR** (400 MHz, $CDCl_3$) δ 7.42 – 7.17 (m, 10H), 4.74 (s, 1H, H-8), 4.61 (d, *J* = 11.6 Hz, 1H, H-11a), 4.52 (d, *J* = 11.6 Hz, 1H, H-11b), 4.11 – 4.03 (m, 1H, H-9a), 3.67 – 3.55 (m, 2H, H-10), 3.28 – 3.23 (m, 1H, H-4), 3.13 (ddd, *J* = 14.9, 10.1, 2.8 Hz, 1H, H-9b), 2.56 – 2.49 (m, 1H, H-2), 2.37 – 2.31 (m, 1H, H-1), 2.23 – 2.14 (m, 1H, H-3a), 2.03 – 1.94 (m, 1H, H-14a), 1.89 (dd, *J* = 12.8, 5.7 Hz, 1H, H-13a), 1.79 (d, *J* = 11.6 Hz, 1H, H-6a), 1.75 – 1.59 (m, 2H, H-13b, H-3b), 1.58 – 1.46 (m, 1H, H-14b), 1.20 (dd, *J* = 11.6, 5.1 Hz, 1H, H-6b); **^{13}C NMR** (101 MHz, $CDCl_3$) δ 176.4,

136.5, 135.4, 131.6, 128.9, 128.3, 128.7, 128.0, 126.7, 88.7, 73.7, 69.0, 49.5, 49.3, 46.2), 43.0), 39.0, 33.4, 30.6, 30.4, 23.8; **HRMS** (ES) found 424.1948 $[MH]^+$, requires 424.1946 for $C_{25}H_{30}NO_3S$.

Preparation of methylallyl lactam **25**

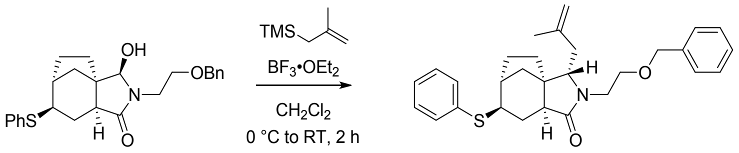

26 **25**

Methylallyltrimethylsilane (0.70 mL, 3.96 mmol, 2.0 eq.) and BF_3•OEt_2 (1.00 mL, 7.92 mmol, 4.0 eq.) were successively added to a solution of **26** (838 mg, 1.98 mmol, 1.0 eq.) in dry CH_2Cl_2 (10 mL) at 0 °C. The reaction mixture was stirred for 1 h at 0 °C and 2 h at RT before being quenched with an aqueous solution of $NaHCO_3$. The aqueous layer was extracted with CH_2Cl_2 (3 × 10 mL), the combined organic layers were dried over $MgSO_4$, filtered and concentrated under reduced pressure. The crude mixture was purified by flash column chromatography (gradient: hexane/EtOAc = 4/1 to 1/1) to yield methylallyl **25** (181 mg, 20%) as a colourless oil. **IR** ν_{max}/cm^{-1} 3068, 2937, 2863, 1687, 1438, 1272, 1107, 899, 737; **^{1}H NMR** (400 MHz, $CDCl_3$) δ 7.44 – 7.15 (m, 10H), 4.78 (dt, J = 17.1, 2.0 Hz, 2H), 4.57 (d, J = 11.8 Hz, 1H), 4.51 (d, J = 11.8 Hz, 1H), 3.96 (dt, J = 14.6, 4.5 Hz, 1H), 3.72 – 3.63 (m, 3H), 3.35 – 3.29 (m, 1H), 3.15 (ddd, J = 14.6, 7.7, 4.5 Hz, 1H), 2.39 (dd, J = 8.8, 4.3 Hz, 1H), 2.32 – 2.06 (m, 5H), 2.06 – 1.92 (m, 2H), 1.76 (s, 3H), 1.74 – 1.65 (m, 1H), 1.65 – 1.51 (m, 2H), 1.13 (dd, J = 12.0, 5.5 Hz, 1H); **^{13}C NMR** (101 MHz, $CDCl_3$) δ 175.0, 142.6, 138.2, 135.8, 131.9, 128.8, 128.4, 127.7, 127.6, 126.7, 113.5, 73.2, 69.0, 62.4, 49.5, 49.2, 45.6, 41.6, 38.9, 37.4, 35.8, 30.3, 29.3, 22.6, 22.5; **HRMS** (ES) found 484.2284 $[MNa]^+$, requires 484.2286 for $C_{29}H_{35}NO_2NaS$.

Preparation of vinyl sulfide **27**

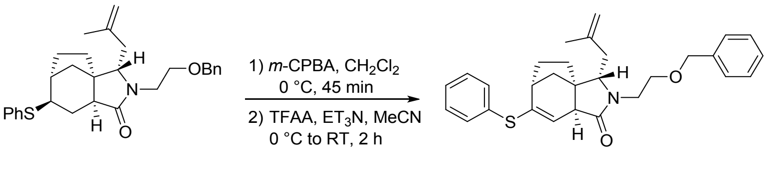

25 **27**

m-CPBA (77%, 70 mg, 0.29 mmol, 1.1 eq.) was slowly added to a solution of **25** (125 mg, 0.27 mmol, 1.0 eq.) in $CHCl_3$ (3 mL) at 0 °C. After 45 min at 0 °C the mixture was quenched with an

aqueous solution of $NaHCO_3$. The aqueous layer was extracted with CH_2Cl_2 (3 × 5 mL), the combined organic layers were dried over $MgSO_4$, filtered and concentrated under reduced pressure. The residue was dissolved in dry MeCN (2 mL) then Et_3N (94 µL, 0.67 mmol, 2.5 eq.) was added at 0 °C. After 5 min, TFAA (94 µL, 0.67 mmol, 2.5 eq.) in dry MeCN (0.5 mL) was added dropwise. The mixture was stirred for 1 h at 0 °C and 1 h at RT before being quenched with an aqueous solution of $NaHCO_3$. The aqueous layer was extracted with CH_2Cl_2 (3 × 5 mL), the combined organic layers were dried over $MgSO_4$, filtered and concentrated under reduced pressure. The crude mixture was purified by flash column chromatography (gradient: hexane/EtOAc = 3/1 to 2/1) to give vinyl sulfide **27** (105 mg, 85% after 2 steps) as a colourless oil. **IR** ν_{max}/cm^{-1} 3070, 3035, 2940, 2861, 1684, 1439, 1182, 1099, 1024, 740; **^{1}H NMR** (400 MHz, $CDCl_3$) δ 7.29 – 7.12 (m, 10H), 5.72 (d, *J* = 4.1 Hz, 1H), 4.72 (dt, *J* = 9.9, 1.4 Hz, 2H), 4.43 (d, *J* = 11.8 Hz, 1H), 4.39 (d, *J* = 11.8 Hz, 1H), 3.86 (dt, *J* = 14.5, 4.4 Hz, 1H), 3.67 (app t, *J* = 6.5 Hz, 1H), 3.55 – 3.50 (m, 2H), 3.05 (dt, *J* = 14.5, 5.8 Hz, 1H), 2.83 (d, *J* = 4.1 Hz, 1H), 2.35 – 2.31 (m, 1H), 2.20 (d, *J* = 6.5 Hz, 2H), 1.85 – 1.78 (m, 2H), 1.77 – 1.73 (m, 1H), 1.70 (s, 3H), 1.68 – 1.64 (m, 1H), 1.60 – 1.54 (m, 1H), 1.32 (dd, *J* = 10.8, 4.4 Hz, 1H); **^{13}C NMR** (101 MHz, $CDCl_3$) δ 173.1, 143.9, 138.1, 133.6, 131.9, 129.0, 128.4, 127.7, 127.2, 120.1, 114.0, 73.1, 68.9, 62.2, 52.1, 48.9, 41.4, 40.9, 40.8, 39.7, 32.8, 30.6, 22.4; 1 signal in the CH aromatic region were not observed due to overlap; **HRMS** (ES) found 482.2123 [MNa]$^+$, requires 482.2130 for $C_{29}H_{33}NO_2SNa$.

Preparation of tetracycle **28**

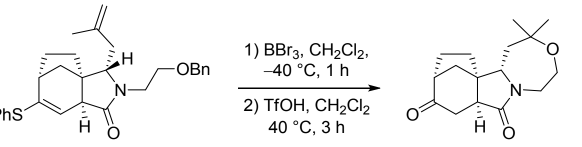

27 **28**

A solution of BBr_3 (1.0 M in CH_2Cl_2, 0.59 mL, 0.59 mmol, 3.0 eq.) was added to a solution of **27** (90 mg, 0.19 mmol, 1.0 eq.) in dry CH_2Cl_2 (2 mL) at –40 °C. The mixture was stirred for 1 h at −40 °C before being quenched with water. The pH was adjusted to pH = 4-5 with an aqueous solution of 2 N NaOH. The aqueous layer was extracted with EtOAc (5 × 5 mL), the combined organic layers were dried over $MgSO_4$, filtered and concentrated under reduced pressure. The residue was dissolved in dry CH_2Cl_2 (3 mL) and triflic acid (138 µL, 1.57 mmol, 5.0 eq.) was added at RT. The mixture was stirred 1 h at RT and 3 h at 40 °C before being quenched with a saturated aqueous solution of $NaHCO_3$. The aqueous layer was extracted with CH_2Cl_2 (3 × 5 mL), the combined organic layers were dried over $MgSO_4$, filtered and concentrated under reduced pressure. The crude mixture was purified by flash column chromatography (gradient: hexane/EtOAc = 1/1 to 0/1) to give tetracyclic compound **28** (17 mg, 33% after 2 steps) as a white powder. **m.p.** 149 – 151 °C; **IR** ν_{max}/cm^{-1} 2968, 2940, 2876,

1713, 1684, 1445, 1369, 1238, 1081; **^{1}H NMR** (400 MHz, $CDCl_3$) δ 4.20 – 4.12 (m, 1H), 3.69 – 3.63 (m, 1H), 3.61 – 3.53 (m, 2H), 2.97 (ddd, *J* = 13.6, 10.5, 2.8 Hz, 1H), 2.79 – 2.73 (m, 1H), 2.71 – 2.64 (m, 2H), 2.61 – 2.55 (m, 1H), 2.22 – 2.10 (m, 1H), 1.96 (d, *J* = 12.3 Hz, 1H), 1.86 (app t, *J* = 14.0 Hz, 2H), 1.82 – 1.70 (m, 3H), 1.65 (dd, *J* = 12.3, 4.9 Hz, 1H), 1.25 (s, 3H), 1.21 (s, 3H); **^{13}C NMR** (101 MHz, $CDCl_3$) δ 211.8, 173.4, 74.7, 62.5, 61.1, 48.9, 48.2, 44.8, 44.4, 43.9, 37.7, 33.6, 32.2, 28.4, 27.3, 26.4; **HRMS** (ES) found 300.1569 $[MNa]^+$, requires 300.1576 for $C_{16}H_{23}NO_3Na$.

Preparation of prenylated keto-lactam **29**

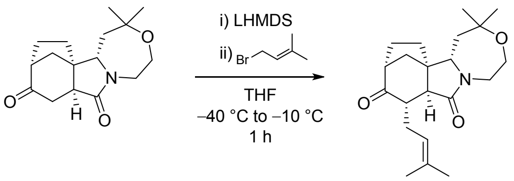

28 **29**

A solution of LHMDS (1.0 M in THF, 0.21 mmol, 0.21 mL, 1.2 eq.) was added to a solution of **28** (50 mg, 0.18 mmol, 1.0 eq.) in dry THF (2 mL) at −40 °C. After 15 min at −40 °C, prenyl bromide (63 µL, 0.54 mmol, 3.0 eq.) was added. The reaction mixture was stirred for 1 h at −40 °C, then allowed to warm to −10 °C and stirred for 5 h before being quenched with water. The aqueous layer was extracted with EtOAc (3 × 5 mL), the combined organic layers were dried over $MgSO_4$, filtered and concentrated under reduced pressure. The crude mixture was purified by flash column chromatography (gradient: hexane/EtOAc = 2/1 to 1/1) to yield prenylated compound **29** (28 mg, 45%) as a colourless oil. **IR** v_{max}/cm^{-1} 3071, 3023, 2998, 2929, 1876, 1480, 1435, 1089, 741; **^{1}H NMR** (400 MHz, $CDCl_3$) δ 4.98 – 4.90 (m, 1H), 4.17 (dt, *J* = 14.0, 2.1 Hz, 1H), 3.68 – 3.57 (m, 2H), 3.54 (d, *J* = 10.5 Hz, 1H), 2.95 (ddd, *J* = 14.0, 10.2, 3.2 Hz, 1H), 2.74 (dd, *J* = 8.3, 4.3 Hz, 1H), 2.71 – 2.63 (m, 2H), 2.50 – 2.40 (m, 1H), 2.19 (d, *J* = 6.3, 1.5 Hz, 1H), 2.14 – 2.02 (m, 1H), 1.97 (dt, *J* = 11.9, 1.7 Hz, 1H), 1.90 – 1.83 (m, 1H), 1.80 – 1.69 (m, 2H), 1.67 (s, 3H), 1.63 (s, 3H), 1.62 – 1.58 (m, 1H), 1.57 – 1.46 (m, 2H), 1.24 (s, 3H, H-16), 1.20 (s, 3H); **^{13}C NMR** (101 MHz, $CDCl_3$) δ 213.8, 174.2, 135.1, 120.1, 74.7, 62.5, 61.2, 48.8, 48.3, 47.6, 45.0, 44.7, 44.3, 37.2, 32.2, 28.7, 28.4, 27.2, 26.4, 25.9, 18.2; **HRMS** (ES) found 386.2204 $[MNa]^+$, requires 386.2202 for $C_{21}H_{31}NO_3Na$.

Preparation of dienyl lactam **30**

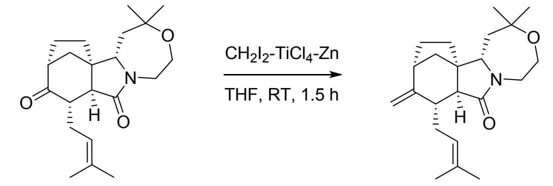

CH_2I_2 (0.4 mL, 5.00 mmol, 5.0 eq.) was added to a suspension of zinc (0.58 g, 9.00 mmol, 9.0 eq.) and $PbCl_2$ (15 mg, 0.05 mmol, 0.05 eq.) in dry THF (10 mL) at RT. After 0.5 h, $TiCl_4$ (1.0 M in CH_2Cl_2, 1.00 ml, 1.00 mmol, 1.0 eq.) was added at 0 °C and the mixture was stirred for 0.5 h at RT. A solution of [CH_2I_2-$TiCl_4$-Zn] (0.1 M in THF, 1.00 mL, 0.10 mmol, 1.3 eq.) was added to a solution of **29** (27 mg, 0.08 mmol, 1.0 eq.) in dry THF (1 mL) at RT. After being stirred for 1.5 h, the mixture was diluted with EtOAc (5 mL), the organic layer was washed with an aqueous solution of 1 M HCl (2 × 4 mL), dried over $MgSO_4$, filtered and concentrated under reduced pressure. The crude mixture was purified by flash column chromatography (gradient: hexane/EtOAc = 2/1 to 1/1) to yield olefin **30** (12 mg, 45%) as a colourless oil. **^{1}H NMR** (400 MHz, $CDCl_3$) δ 5.19 – 5.13 (m, 1H), 4.77 – 4.74 (m, 1H), 4.69 – 4.65 (m, 1H), 4.15 (dt, *J* = 13.9, 2.1 Hz, 1H), 3.67 – 3.54 (m, 2H), 3.43 (dd, *J* = 10.1, 1.5 Hz, 1H), 2.97 – 2.89 (m, 1H), 2.82 – 2.75 (m, 2H), 2.44 – 2.30 (m, 2H), 2.16 (d, *J* = 4.2 Hz, 1H), 2.06 – 1.95 (m, 1H), 1.87 – 1.73 (m, 2H), 1.73 (s, 3H), 1.67 – 1.52 (m, 7H), 1.37 (dd, *J* = 11.3, 4.9 Hz, 1H), 1.24 (s, 3H), 1.20 (s, 3H); **^{13}C NMR** (101 MHz, $CDCl_3$) δ 174.7, 155.0, 133.3, 122.4, 108.3, 74.8, 62.7, 61.5, 51.2, 48.4, 44.1 × 2, 43.2, 40.5, 36.2, 35.6, 31.7, 30.2, 28.5, 27.3, 25.9, 18.2.

Preparation of **epi-1**

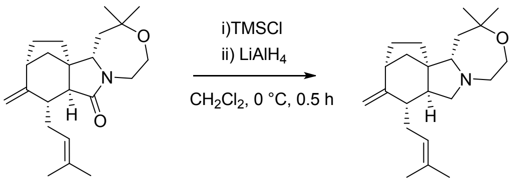

TMSCl (6 µL, 0.042 mmol, 1.2 eq.) was added to a solution of **30** (12 mg, 0.035 mmol, 1.0 eq.) in dry CH_2Cl_2 (1 mL) at 0 °C. The reaction mixture was stirred for 15 min at 0 °C, then a solution of $LiAlH_4$ (2.4 M in THF, 22 µL, 0.052 mmol, 1.5 eq.) was added. After 0.5 h at 0 °C, the reaction mixture was quenched with an aqueous solution of 2 N NaOH. The aqueous layer was extracted with CH_2Cl_2 (3 × 3 mL), the combined organic layers were dried over $MgSO_4$, filtered and concentrated under reduced pressure. The crude mixture was purified by flash column chromatography (gradient: hexane/EtOAc = 1/1 to 0/1) to yield **epi-1** (10 mg, 90%) as a colourless oil. **IR** ν_{max}/cm^{-1} 3080, 2928, 2868, 2800, 1364, 1169, 1077, 882; **^{1}H NMR** (400 MHz, $CDCl_3$) δ 5.14 – 5.07 (m, 1H), 4.81 – 4.77 (m, 1H), 4.70 – 4.67

(m, 1H), 3.85 – 3.75 (m, 1H), 3.56 (ddd, J = 13.6, 3.3, 1.9 Hz, 1H), 3.27 (app t, J = 8.0 Hz, 1H), 3.11 – 3.01 (m, 1H), 2.72 (dd, J = 8.0, 4.9 Hz, 1H), 2.48 (d, J = 9.8 Hz, 1H), 2.36 – 2.14 (m, 3H), 2.14 – 2.04 (m, 2H), 1.94 – 1.71 (m, 2H), 1.66 (s, 3H), 1.64 –1.54 (m, 5H), 1.49 (d, J = 11.3 Hz, 1H), 1.39 – 1.30 (m, 3H), 1.21 (s, 3H), 1.19 – 1.15 (m, 4H); **^{13}C NMR** (101 MHz, $CDCl_3$) δ 171.0, 148.7, 122.1, 105.8, 74.9, 65.2, 62.8, 62.1, 58.4, 52.3, 48.3, 43.2, 40.7, 39.8, 35.0, 33.6, 32.4, 29.1, 28.3, 27.6, 25.8, 18.0; **HRMS** (ES) found 330.2798 [MH]+, requires 330.2797 for $C_{22}H_{36}NO$.

*Synthesis of sulfone **23** from **14***

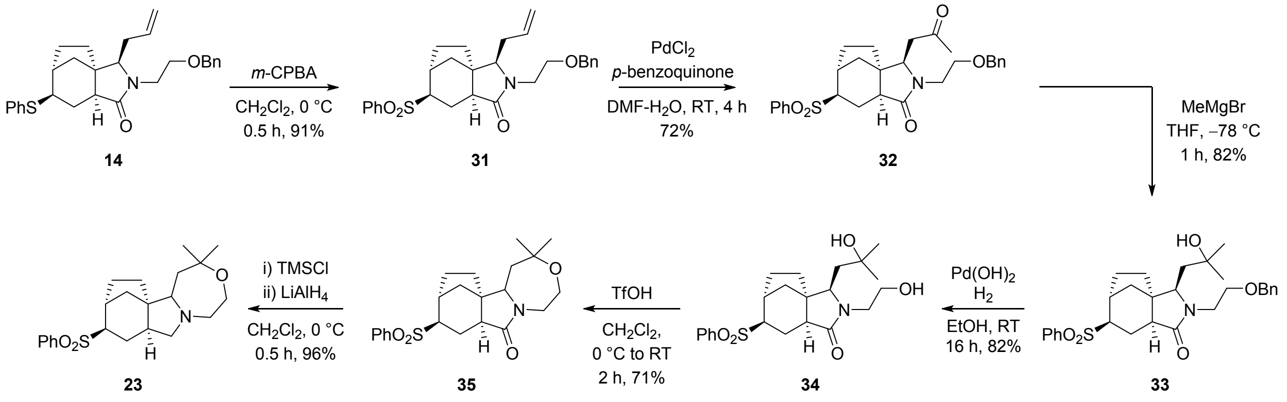

Preparation of sulfone **31**

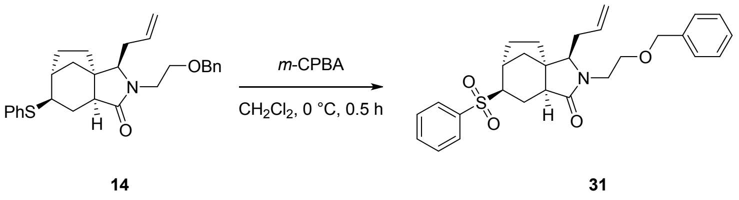

m-CPBA (77%, 437 mg, 2.54 mmol, 2.0 eq.) was added to a solution of **14** (568 mg, 1.27 mmol, 1.0 eq.) in CH_2Cl_2 (15 mL) at 0 °C. The mixture was stirred for 0.5 h at 0 °C then quenched with a saturated aqueous solution of $NaHCO_3$. The aqueous layer was extracted with CH_2Cl_2 (3 × 8 mL), the combined organic layers were dried over $MgSO_4$, filtered and concentrated under reduced pressure. The crude mixture was purified by flash column chromatography (gradient: hexane/EtOAc = 2/1 to 1/1) to yield sulfone **31** (553 mg, 91%) as a colourless gum. **IR** v_{max}/cm^{-1} 3068, 2956, 2873, 1720, 1682, 1639, 1446, 1304, 1145, 1085, 727; **^{1}H NMR** (400 MHz, $CDCl_3$) δ 7.94 – 7.53 (m, 5H), 7.41 – 7.27 (m, 5H), 5.79 – 5.67 (m, 1H), 5.16 – 5.04 (m, 2H), 4.51 (d, J = 11.9 Hz, 1H), 4.45 (d, J = 11.9 Hz, 1H), 3.87 (dt, J = 14.4, 5.4 Hz, 1H), 3.75 (dd, J = 8.5, 4.3 Hz, 1H), 3.62 – 3.55 (m, 2H), 3.23 (dt, J = 14.4, 5.4 Hz, 1H), 3.08 – 3.01 (m, 1H), 2.93 (dd, J = 12.3, 6.5 Hz, 1H), 2.64 – 2.54 (m, 1H), 2.29

– 2.20 (m, 1H), 2.20 – 2.11 (m, 1H), 2.11 – 2.02 (m, 1H), 1.92 – 1.83 (m, 1H), 1.80 – 1.70 (m, 1H), 1.69 – 1.60 (m, 2H), 1.54 – 1.46 (m, 1H), 1.46 – 1.34 (m, 2H); **^{13}C NMR** (101 MHz, $CDCl_3$) δ 175.4, 138.0, 137.6, 134.4, 133.7, 129.3, 128.8, 128.4, 127.7, 127.6, 118.0, 72.9, 67.7, 66.5, 60.8, 49.0, 47.3, 40.1, 36.0, 33.8, 31.9, 31.5, 28.5, 19.9; **HRMS** (ES) found 502.2025 $[MNa]^+$, requires 502.2028 for $C_{28}H_{33}NO_4NaS$.

Preparation of ketone **32**

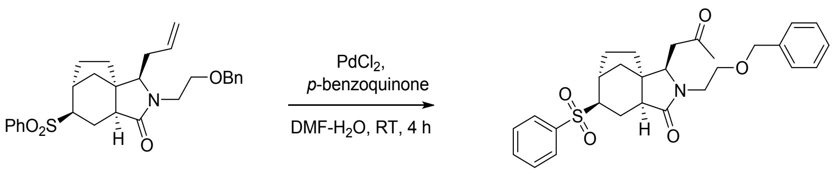

31 **32**

$PdCl_2$ (77 mg, 0.44 mmol, 0.15 eq.) and *p*-benzoquinone (315 mg, 2.92 mmol, 1.0 eq.) were added to a solution of sulfone **31** (1.40 g, 2.92 mmol, 1.0 eq.) in a mixture of DMF (12 mL) and H_2O (2 mL) at RT. The reaction mixture was stirred for 4 h at RT before being quenched with an aqueous solution of 1 M HCl. The aqueous layer was extracted with EtOAc (3 × 20 mL), the combined organic layers were washed with water (5 × 10 mL), dried over $MgSO_4$, filtered and concentrated under reduced pressure. The crude mixture was purified by flash column chromatography (gradient: hexane/ EtOAc = 1/2 to 1/4) to furnish ketone **32** (1.04 g, 72%) as a white powder. **m.p.** 48 – 50 °C; **IR** ν_{max}/cm^{-1} 3062, 3038, 2962, 2874, 1715, 1680, 1446, 1303, 1145, 1086, 724; **^{1}H NMR** (400 MHz, $CDCl_3$) δ 7.93 – 7.53 (m, 5H), 7.43 – 7.25 (m, 5H), 4.47 (s, 2H), 4.26 (app t, *J* = 6.3 Hz, 1H), 3.63 – 3.57 (m, 1H), 3.57 – 3.48 (m, 2H), 3.32 – 3.24 (m, 1H), 3.08 – 3.01 (m, 1H), 2.99 – 2.91 (m, 2H), 2.44 (dd, *J* = 18.0, 6.3 Hz, 1H), 2.21 – 2.13 (m, 1H), 2.13 – 2.05 (m, 1H), 2.03 (s, 3H), 1.90 – 1.75 (m, 2H), 1.75 – 1.70 (m, 1H), 1.69 – 1.62 (m, 1H), 1.51 (dd, *J* = 11.2, 9.3 Hz, 1H), 1.45 – 1.35 (m, 1H), 1.07 (dd, *J* = 11.8, 4.8 Hz, 1H); **^{13}C NMR** (101 MHz, $CDCl_3$) δ 206.8, 175.3, 137.8, 137.6, 133.8), 129.3, 128.7, 128.5, 128.0, 127.9, 73.3, 68.3, 66.5, 57.4, 48.4, 46.6, 44.1, 41.6, 35.6, 31.6, 31.5, 30.2, 29.5, 20.1; **HRMS** (ES) found 518.1973 $[MNa]^+$, requires 518.1977 for $C_{28}H_{33}NO_5NaS$.

Preparation of alcohol **33**

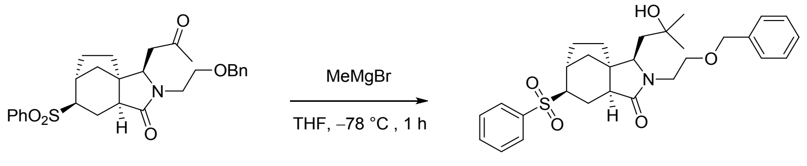

32 **33**

A solution of MeMgBr (3.0 M in Et_2O, 0.56 mL, 1.76 mmol, 2.0 eq.) was added to a solution of **32** (436 mg, 0.88 mmol, 1.0 eq.) in dry THF (6 mL) at −78 °C. The mixture was stirred for 1 h at −78 °C before being quenched with water. The aqueous layer was extracted with EtOAc (3 × 8 mL), the combined organic layers were washed with a saturated aqueous solution of NH_4Cl, dried over $MgSO_4$, filtered and concentrated under reduced pressure. The crude mixture was purified by flash column chromatography (gradient: hexane/EtOAc = 1/1 to 0/1) to furnish alcohol **33** (367 mg, 82%) as a white powder. **m.p.** = 53 – 56 °C; **IR** ν_{max}/cm^{-1} 3438, 3065, 2963, 2927, 2871, 1667, 1446, 1303, 1144; **^{1}H NMR** (400 MHz, $CDCl_3$) δ 7.94 – 7.55 (m, 5H), 7.39 – 7.27 (m, 5H), 4.50 (d, *J* = 11.3 Hz, 1H), 4.45 (d, *J* = 11.3 Hz, 1H), 3.87 (dd, *J* = 5.5, 3.1 Hz, 1H), 3.80 – 3.72 (m, 2H), 3.70 – 3.59 (m, 2H), 3.28 (s, 1H, OH), 3.08 – 3.01 (m, 1H), 2.98 (dd, *J* = 11.7, 6.9 Hz, 1H), 2.15 (dd, *J* = 11.1, 7.8 Hz, 1H), 2.12 – 2.03 (m, 1H), 1.93 – 1.84 (m, 1H), 1.78 – 1.64 (m, 3H), 1.64 – 1.55 (m, 2H), 1.51 – 1.40 (m, 2H), 1.24 (dd, *J* = 12.2, 4.8 Hz, 1H), 1.19 (s, 3H), 1.01 (s, 3H); **^{13}C NMR** (101 MHz, $CDCl_3$) δ 175.6, 137.6, 137.1, 133.7, 129.3, 128.8, 128.6, 128.4, 128.2, 73.8, 69.4, 69.2, 66.4, 59.4, 48.2, 48.2, 42.2, 40.6, 33.9, 32.1, 31.7, 31.0, 28.9, 28.7, 19.9; **HRMS** (ES) found 534.2293 [MNa]$^+$, requires 534.2290 for $C_{29}H_{37}NO_5SNa$.

Preparation of diol **34**

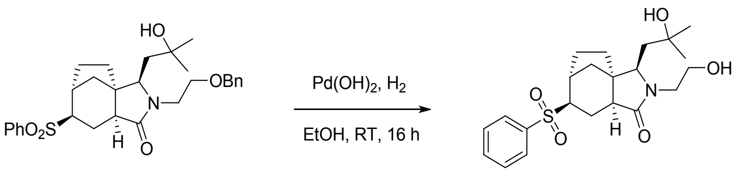

33 **34**

Compound **33** (60 mg, 0.12 mmol, 1.0 eq.) and $Pd(OH)_2$ (3.3 mg, 0.02 mmol, 0.20 eq.) were dissolved in ethanol (2 mL) and put under hydrogen pressure (balloon). The reaction mixture was stirred for 16 h at RT then filtered through Celite® and concentrated under reduced pressure. The crude mixture was purified by flash column chromatography (gradient: hexane/EtOAc = 0/1 to 0/1 + 5% MeOH) to furnish diol **34** (40 mg, 82%) as a white powder. **m.p.** 77 – 80 °C; **IR** ν_{max}/cm^{-1} 3393, 3095, 3065, 2961, 2884, 1662, 1447, 1381, 1303, 1144, 1084, 723. **^{1}H NMR** (400 MHz, $CDCl_3$) δ 7.90 – 7.58 (m, 5H), 4.01 (app t, *J* = 4.3 Hz, 1H), 3.82 – 3.75 (m, 2H), 3.72 – 3.66 (m, 1H), 3.54 (ddd, *J* = 14.6, 8.1, 3.5 Hz, 1H), 3.11 – 3.04 (m, 1H), 2.99 (dd, *J* = 11.4, 7.0 Hz 1H), 2.26 (dd, *J* = 11.0, 7.9

Hz, 1H), 2.16 – 2.08 (m, 1H), 1.93 – 1.82 (m, 1H), 1.81 – 1.75 (m, 1H), 1.75 – 1.68 (m, 3H), 1.68 – 1.63 (m, 1H), 1.56 – 1.43 (m, 2H), 1.35 (s, 3H), 1.31 (dd, J = 13.2, 5.9 Hz, 1H), 1.27 (s, 3H); **^{13}C NMR** (101 MHz, $CDCl_3$) δ 176.6, 137.6, 133.8, 129.3, 128.8, 70.0, 66.4, 62.4, 59.5, 48.6, 48.4, 43.5, 41.5, 34.2, 31.8, 31.6, 31.6, 29.8, 29.3, 19.9; **HRMS** (ES) found 444.1828 $[MNa]^+$, requires 444.1821 for $C_{22}H_{31}NO_5NaS$.

Preparation of tetracycle **35**

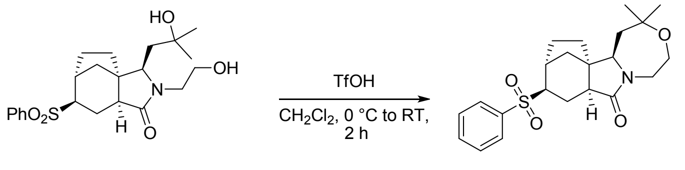

34 35

Triflic acid (0.30 mL, 3.36 mmol, 2.0 eq.) was added to a solution of **34** (470 mg, 1.12 mmol, 1.0 eq.) in dry CH_2Cl_2 (5 mL) at 0 °C. The mixture was stirred for 0.5 h at 0 °C and 2 h at RT before being quenched with a saturated aqueous solution of $NaHCO_3$. The aqueous layer was extracted with CH_2Cl_2 (3 x 8 mL), the combined organic layers were dried over $MgSO_4$, filtered and concentrated under reduced pressure. The crude mixture was purified by flash column chromatography (gradient: hexane/EtOAc = 1/1 to 0/1) to give tetracyclic compound **35** (320 mg, 71%) as a white powder. **m.p.** 65 – 69 °C; **IR** ν_{max}/cm^{-1} 3059, 2968, 2875, 1680, 1446, 1426, 1303, 1145, 1084, 918, 725; **^{1}H NMR** (400 MHz, $CDCl_3$) δ 7.90 – 7.85 (m, 2H), 7.68 – 7.62 (m, 1H), 7.58 – 7.53 (m, 2H), 3.82 – 3.73 (m, 2H), 3.71 – 3.64 (m, 2H), 3.22 – 3.15 (m, 1H), 3.07 (dd, J = 7.4, 4.7 Hz, 1H), 2.95 (dd, J = 12.3, 6.6 Hz, 1H), 2.17 (dd, J = 11.8, 7.6 Hz, 1H), 2.11 – 2.05 (m, 1H), 1.86 (ddd, J = 14.2, 7.6, 6.6 Hz, 1H), 1.77 – 1.69 (m, 1H), 1.66 – 1.55 (m, 4H), 1.57 – 1.50 (m, 1H), 1.49 – 1.43 (m, 1H), 1.23 (s, 6H), 1.08 (dd, J = 12.1, 4.7 Hz, 1H); **^{13}C NMR** (101 MHz, $CDCl_3$) δ 175.3, 137.5, 133.8, 129.4, 128.8, 74.5, 66.4, 60.2, 58.3, 48.3, 46.6, 45.8, 41.9, 34.6, 32.1, 31.6, 29.0, 28.4, 26.7, 20.1; **HRMS** (ES) found 426.1717 $[MNa]^+$, requires 426.1715 for $C_{22}H_{29}NO_4NaS$.

Preparation of sulfone **23**

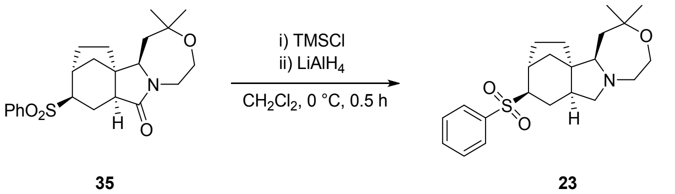

TMSCl (120 µL, 0.95 mmol, 1.2 eq.) was added to a solution of **35** (318 mg, 0.79 mmol, 1.0 eq.), in dry CH_2Cl_2 (5 mL) at 0 °C. The reaction mixture was stirred for 15 min before addition of a solution of $LiAlH_4$ (2.4 M in THF, 0.60 mL, 1.42 mmol, 1.8 eq.). After 0.5 h at 0 °C, the reaction mixture was quenched with an aqueous solution of 2 N NaOH (aq). The aqueous layer was extracted with EtOAc (3 × 8 mL), the combined organic layers were dried over $MgSO_4$, filtered and concentrated under reduced pressure. The crude mixture was purified by flash column chromatography (gradient: hexane/EtOAc = 1/1 to 0/1) to give sulfone **23** (294 mg, 96%) as a white powder. **m.p** 60 – 62 °C; **IR** v_{max}/cm^{-1} 3066, 2937, 2868, 2800, 1446, 1304, 1143, 1085, 725; **^{1}H NMR** (400 MHz, $CDCl_3$) δ 7.91 – 7.51 (m, 5H), 3.78 (dd, *J* = 13.5, 10.6 Hz, 1H), 3.51 (ddd, *J* = 13.5, 3.2, 2.0 Hz, 1H), 2.94 (dd, *J* = 12.5, 3.2 Hz, 1H), 2.86 (dd, *J* = 11.6, 4.8 Hz, 1H), 2.84 – 2.79 (m, 1H), 2.70 (d, *J* = 9.3 Hz, 1H), 2.47 (d, *J* = 9.6 Hz, 1H), 2.41 (d, *J* = 9.3 Hz, 1H), 2.25 (ddd, *J* = 12.5, 10.6, 2.0 Hz, 1H), 2.07 – 1.96 (m, 1H), 1.76 – 1.63 (m, 3H), 1.62 – 1.53 (m, 2H), 1.47 – 1.36 (m, 2H), 1.31 – 1.23 (m, 2H), 1.18 (s, 3H), 1.15 (s, 3H), 0.91 (dd, *J* = 12.1, 4.8 Hz, 1H); **^{13}C NMR** (101 MHz, $CDCl_3$) δ 138.0, 133.5, 129.1, 128.8, 74.7, 67.9, 64.3, 61.8, 61.4, 58.4, 52.8, 43.0, 41.4, 33.9, 31.9, 31.6, 28.8, 28.6, 27.6, 25.0; **HRMS** (ES) found 390.2094 $[MH]^+$, requires 390.2103 for $C_{22}H_{32}NO_3S$.

*Formation of **1-HCl** and **1-AcOH** from concavine **1***

Preparation of **1-HCl**

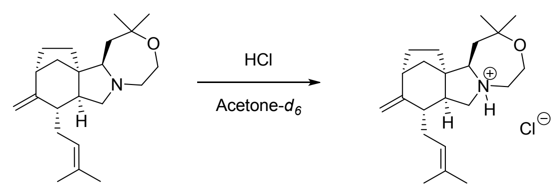

A solution of HCl in acetone-*d_6* (0.24 M, 0.1 mL, 0.024 mmol, 1.0 eq.) was added to a solution of **1** in acetone-*d_6* (8 mg, 0.024 mmol, 1.0 eq.) in a NMR tube. **^{1}H NMR** (400 MHz, acetone-*d_6*) δ 5.18 – 5.11 (m, 1H), 4.86 – 4.83 (m, 1H), 4.80 – 4.77 (m, 1H), 4.17 (ddd, *J* = 14.6, 10.7, 2.7 Hz, 1H), 3.71 (app t, *J* = 9.8 Hz, 1H), 3.61 (dt, *J* = 14.6, 2.7 Hz, 1H), 3.48 – 3.35 (m, 2H), 3.30 – 3.20 (m, 1H), 3.01 – 2.92 (m, 1H), 2.88 – 2.80 (m, 1H), 2.57 (dd, *J* = 16.3, 9.8 Hz, 1H), 2.43 (d, *J* = 11.9 Hz, 1H) 2.40 – 2.36 (m, 1H), 2.28 – 2.20 (m 2H), 2.03 – 1.93 (m, 2H), 1.80 (d, *J* = 16.3 Hz, 1H), 1.68 (s, 3H), 1.67 – 1.64

(m, 1H), 1.63 (s, 3H), 1.56 – 1.43 (m, 2H), 1.22 (s, 3H), 1.17 (s, 3H), 1.09 (dd, J = 11.9, 4.5 Hz, 1H); **^{13}C NMR** (101 MHz, acetone-d_6) δ 155.1, 132.3, 122.1, 107.7, 74.2, 66.3, 58.5, 58.1, 56.1, 48.3, 42.0, 40.4, 35.8, 32.8, 32.5, 30.3, 30.2, 27.2, 25.9, 25.0, 17.2.

Preparation of **1-AcOH**

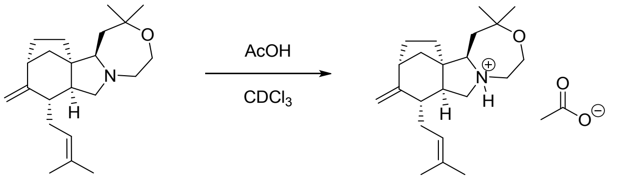

1 **1 AcOH**

A solution of AcOH in $CDCl_3$ (0.18 M, 0.1 mL, 0.018 mmol, 1.0 eq.) was added to a solution of **1** (6 mg, 0.018 mmol, 1.0 eq.) in $CDCl_3$ (0.5 mL) in a NMR tube. **^{1}H NMR** (400 MHz, $CDCl_3$) δ 5.10 – 5.05 (m, 1H), 4.86 – 4.82 (m, 1H), 4.78 – 4.74 (m, 1H), 4.03 (dd, J = 14.4, 10.8 Hz, 1H), 3.58 (dt, J = 14.4, 2.5 Hz, 1H) 3.55 – 3.47 (m, 2H), 3.11 (d, J = 9.6 Hz, 1H), 2.89 – 2.84 (m, 1H), 2.72 (app t, J = 10.9 Hz, 1H), 2.60 – 2.51 (m, 1H), 2.31 (dd, J = 16.0, 9.6 Hz, 1H), 2.26 – 2.17 (m, 3H), 2.02 (s, 3H, AcOH), 1.98 – 1.89 (m, 2H), 1.78 – 1.71 (m, 1H), 1.69 (s, 3H), 1.60 (s, 3H), 1.57 – 1.51 (m, 1H), 1.50 – 1.32 (m, 3H), 1.25 (s, 3H), 1.17 (s, 3H), 1.04 (dd, J = 11.7, 4.4 Hz, 1H); **^{13}C NMR** (101 MHz, $CDCl_3$) δ 176.1 (C=O, AcOH), 155.1, 132.7, 122.1, 108.3, 74.7, 66.2, 59.8, 59.7, 57.4, 52.7, 49.0, 42.0, 40.5, 37.5, 33.5, 33.0, 30.3, 30.1, 28.2, 26.8, 25.8, 22.1 (CH_3, AcOH), 18.0.

Comparison between synthesised and reported concavine

Table 1: ^{1}H NMR data for reported and synthesised concavine **1** in both $CDCl_3$ and acetone-d_6

$CDCl_3$

	Reported	1	Δ ppm
1	2.27	2.13	0.14
2			
3	2.88	2.8	0.08
4_α	1.06	0.97	0.09
4_β	2.07	1.72	0.35
4a			
5_α	1.5	1.5	0
5_β	2.27	1.72	0.55
5a	3.13	2.57	0.56
6			
8_α	3.63	3.54	0.09
8_β	4.08	3.8	0.28
9_α	2.63	2.34	0.29
9_β	3.39	2.96	0.43
10_α	2.78	2.52	0.26
10_β	3.39	2.96	0.43
10a	1.77	1.56	0.21
11_α	1.41	1.26	0.15
11_β	1.5	1.41	0.09
12_α	1.5	1.41	0.09
12_β	1.95	1.91	0.04
13_α	2.17	2.19	-0.02
13_β	2.23	2.19	0.04
14	5.08	5.1	-0.02
15			
16	1.69	1.68	0.01
17	1.61	1.61	0
18_α	4.72	4.73	-0.01
18_β	4.85	4.8	0.05
19	1.26	1.2	0.06
20	1.18	1.17	0.01

Acetone-d_6

	Reported	1	Δ ppm
1	2.19	2.08	0.11
2			
3	2.84	2.75	0.09
4_α	0.99	1	-0.01
4_β	1.86	1.71	0.15
4a			
5_α	1.51	1.52	-0.01
5_β	2.08	1.71	0.37
5a	2.91	2.49	0.42
6			
8_α	3.55	3.4	0.15
8_β	3.95	3.73	0.22
9_α	2.51	2.2	0.31
9_β	3.28	2.87	0.41
10_α	2.63	2.42	0.21
10_β	3.28	2.85	0.43
10a	1.66	1.56	0.1
11_α	1.32	1.24	0.08
11_β	1.48	1.45	0.03
12_α	1.43	1.36	0.07
12_β	1.92	1.94	-0.02
13_α	2.19	2.2	-0.01
13_β	2.19	2.2	-0.01
14	5.08	5.14	-0.06
15			
16	1.67	1.67	0
17	1.59	1.61	-0.02
18_α	4.73	4.72	0.01
18_β	4.86	4.77	0.09
19	1.15	1.11	0.04
20	1.21	1.14	0.07

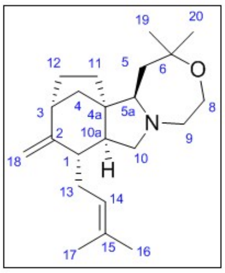

0.1 - 0.3	
0.3 - 0.5	
> 0.5	

Table 2: ^{13}C NMR data for reported and synthesised concavine **1** in both $CDCl_3$ and acetone-d_6

$CDCl_3$

	Reported	1	Δ ppm
1	40.93	41.52	-0.59
2	155.52	157.1	-1.58
3	41.91	41.79	0.12
4	30.28	30.36	-0.08
4a	52.99	53.45	-0.46
5	39.07	41.67	-2.6
5a	65.9	64.82	1.08
6	74.75	74.81	-0.06
8	60.46	61.97	-1.51
9	57.82	58.62	-0.8
10	60.46	61.52	-1.06
10a	49.3	49.63	-0.33
11	33.41	33.5	-0.09
12	33.12	33.39	-0.27
13	30.28	30.01	0.27
14	122.39	123	-0.61
15	132.42	131.8	0.62
16	25.8	25.28	0.52
17	17.99	17.97	0.02
18	108.08	107.21	0.87
19	27.05	27.57	-0.52
20	28.37	28.76	-0.39

Acetone-d_6

	Reported	1	Δ ppm
1	40.84	41.24	-0.4
2	155.63	157.02	-1.39
3	41.88	41.74	0.14
4	29.98	30.23	-0.25
4a	53.04	53.49	-0.45
5	38.92	41.5	-2.58
5a	65.47	64.52	0.95
6	74.78	74.09	0.69
8	60.35	61.5	-1.15
9	57.49	58.47	-0.98
10	60.06	61.38	-1.32
10a	49.08	49.5	-0.42
11	33.37	33	0.37
12	32.98	32.95	0.03
13	30.14	29.91	0.23
14	122.28	123.13	-0.85
15	132.15	131.09	1.06
16	25.67	25.04	0.63
17	17.86	17.12	0.74
18	107.98	106.52	1.46
19	27.96	27.06	0.9
20	28.03	28.17	-0.14

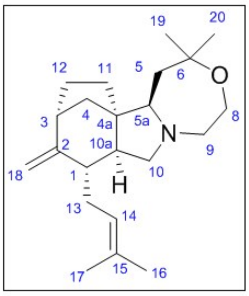

0.5 - 1.0	
1.0 - 1.5	
> 1.5	

Table 3: ^{1}H NMR data for reported concavine and **1-HCl** in acetone-d$_6$

Acetone-d6

	Reported	1-HCl	Δ ppm
1	2.19	2.38	-0.19
2			
3	2.84	2.83	0.01
4_α	0.99	1.09	-0.1
4_β	1.86	2.43	-0.57
4a			
5_α	1.51	1.8	-0.29
5_β	2.08	2.57	-0.49
5a	2.91	3.71	-0.8
6			
8_α	3.55	3.61	-0.06
8_β	3.95	4.17	-0.22
9_α	2.51	2.95	-0.44
9_β	3.28	3.36	-0.08
10_α	2.63	3.24	-0.61
10_β	3.28	3.42	-0.14
10a	1.66	2.01	-0.35
11_α	1.32	1.49	-0.17
11_β	1.48	1.66	-0.18
12_α	1.43	1.49	-0.06
12_β	1.92	1.99	-0.07
13_α	2.19	2.25	-0.06
13_β	2.19	2.25	-0.06
14	5.08	5.14	-0.06
15			
16	1.59	1.63	-0.04
17	1.67	1.68	-0.01
18_α	4.73	4.78	-0.05
18_β	4.86	4.84	0.02
19	1.15	1.17	-0.02
20	1.21	1.22	-0.01

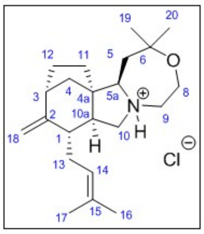

0.1 - 0.3
0.3 - 0.5
> 0.5

Table 4: ^{13}C NMR data for reported concavine and **1-HCl** in acetone-d$_6$

Acetone-d6

	Reported	1-HCl	Δ ppm
1	40.84	40.46	0.38
2	155.63	155.09	0.54
3	41.88	42.04	-0.16
4	29.98	30.27	-0.29
4a	53.04	52.48	0.56
5	38.92	35.75	3.17
5a	65.47	66.29	-0.82
6	74.78	74.27	0.51
8	60.35	58.14	2.21
9	57.49	56.12	1.37
10	60.06	58.47	1.59
10a	49.08	48.31	0.77
11	33.37	32.75	0.62
12	32.98	32.51	0.47
13	30.14	30.18	-0.04
14	122.28	122.14	0.14
15	132.15	132.28	-0.13
16	17.86	17.24	0.62
17	25.67	24.98	0.69
18	107.98	107.65	0.33
19	27.96	25.86	2.1
20	28.03	27.25	0.78

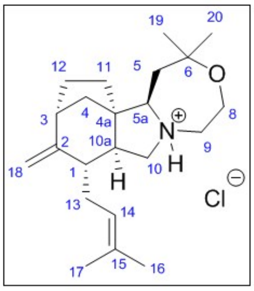

0.5 - 1.0
1.0 - 1.5
> 1.5

Table 5: ^{1}H NMR comparison between reported concavine and **1-AcOH** in $CDCl_3$

	CDCl3		
	Reported	1-AcOH	Δ ppm
1	2.27	2.21	0.06
2			
3	2.88	2.86	0.02
4_α	1.06	1.04	0.02
4_β	2.07	1.94	0.13
4a			
5_α	1.5	1.54	-0.04
5_β	2.27	2.31	-0.04
5a	3.13	3.11	0.02
6			
8_α	3.63	3.58	0.05
8_β	4.08	4.03	0.05
9_α	2.63	2.54	0.09
9_β	3.39	3.51	-0.12
10_α	2.78	2.72	0.06
10_β	3.39	3.52	-0.13
10a	1.77	1.75	0.02
11_α	1.41	1.41	0
11_β	1.5	1.47	0.03
12_α	1.5	1.41	0.09
12_β	1.95	1.92	0.03
13_α	2.17	2.21	-0.04
13_β	2.23	2.21	0.02
14	5.08	5.08	0
15			
16	1.61	1.6	0.01
17	1.69	1.69	0
18_α	4.72	4.75	-0.03
18_β	4.85	4.84	0.01
19	1.26	1.25	0.01
20	1.18	1.17	0.01

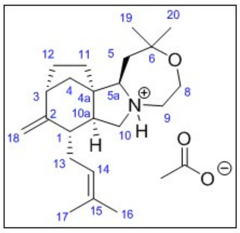

0.1 - 0.3
0.3 - 0.5
> 0.5

Table 6:^{13}C NMR comparison between reported concavine and **1-AcOH** in $CDCl_3$

	CDCl3		
	Reported	1-AcOH	Δ ppm
1	40.93	40.51	0.42
2	155.52	155.1	0.42
3	41.91	42.01	-0.1
4	30.28	30.14	0.14
4a	52.99	52.72	0.27
5	39.07	38.25	0.82
5a	65.9	66.21	-0.31
6	74.75	74.71	0.04
8	60.46	59.7	0.76
9	57.82	57.36	0.46
10	60.46	59.81	0.65
10a	49.3	49.02	0.28
11	33.41	33.46	-0.05
12	33.12	33	0.12
13	30.28	30.29	-0.01
14	122.39	122.1	0.29
15	132.42	132.7	-0.28
16	17.99	18	-0.01
17	25.8	25.81	-0.01
18	108.08	108.4	-0.32
19	27.05	26.82	0.23
20	28.37	28.19	0.18

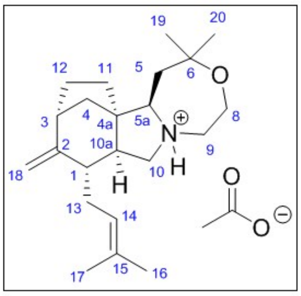

0.5 - 1.0
1.0 - 1.5
> 1.5

Table 7:^{1}H NMR comparison between reported concavine and **epi-1** in $CDCl_3$

CDCl3

	Reported	**epi-1**	Δ ppm
1	2.27	2.1	0.17
2			
3	2.88	2.72	0.16
4_α	1.06	1.16	-0.1
4_β	2.07	1.49	0.58
4a			
5_α	1.5	1.58	-0.08
5_β	2.27	1.78	0.49
5a	3.13	2.48	0.65
6			
8_α	3.63	3.56	0.07
8_β	4.08	3.79	0.29
9_α	2.63	2.3	0.33
9_β	3.39	3.06	0.33
10_α	2.78	1.87	0.91
10_b	3.39	3.27	0.12
10a	1.77	1.58	0.19
11_α	1.41	1.34	0.07
11_β	1.5	1.34	0.16
12_α	1.5	1.34	0.16
12_β	1.95	2.1	-0.15
13_α	2.17	2.22	-0.05
13_β	2.23	2.22	0.01
14	5.08	5.1	-0.02
15			
16	1.61	1.61	0
17	1.69	1.66	0.03
18_α	4.72	4.68	0.04
18_β	4.85	4.79	0.06
19	1.26	1.21	0.05
20	1.18	1.18	0

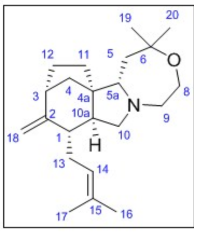

0.1 - 0.3
0.3 - 0.5
> 0.5

Table 8:^{13}C NMR comparison between reported concavine and **epi-1** in $CDCl_3$

CDCl3

	Reported	**epi-1**	Δ ppm
1	40.93	39.77	1.16
2	155.52	171.1	-15.58
3	41.91	40.66	1.25
4	30.28	35	-4.72
4a	52.99	52.28	0.71
5	39.07	43.19	-4.12
5a	65.9	65.17	0.73
6	74.75	74.93	-0.18
8	60.46	62.05	-1.59
9	57.82	58.35	-0.53
10	60.46	62.75	-2.29
10a	49.3	48.3	1
11	33.41	33.57	-0.16
12	33.12	32.39	0.73
13	30.28	28.3	1.98
14	122.39	122.07	0.32
15	132.42	148.7	-16.28
16	17.99	18	-0.01
17	25.8	25.84	-0.04
18	108.08	105.83	2.25
19	27.05	27.64	-0.59
20	28.37	29.1	-0.73

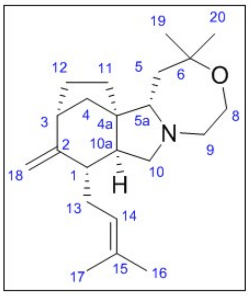

0.5 - 1.0
1.0 - 1.5
> 1.5

Crystal Structure Determination of 23:

Crystal Data for $C_{22}H_{31}NO_3S$, CH_2Cl_2 (M =474.46 g/mol): monoclinic, space group I2/a (no. 15), a = 26.9274(3) Å, b = 5.87596(8) Å, c = 29.0682(4) Å, β = 90.0555(12)°, V = 4599.29(10) Å^3, Z = 8, T = 100(2) K, μ(Cu Kα) = 3.587 mm^{-1}, $Dcalc$ = 1.370 g/cm^3, 19675 reflections measured (6.082° ≤ 2Θ ≤ 136.468°), 4194 unique (R_{int} = 0.0341, R_{sigma} = 0.0230) which were used in all calculations. The final R_1 was 0.0754 (I > 2σ(I)) and wR_2 was 0.2247 (all data).

The dataset was measured on an Agilent SuperNova diffractometer using an Atlas detector. The data collection was driven and processed and an absorption correction was applied using CrysAlisPro.[S1] The structure was solved using ShelXS[S2] and refined by a full-matrix least-squares procedure on F^2 in ShelXL.[S2] All non-hydrogen atoms were refined with anisotropic displacement parameters. All hydrogen atoms were added at calculated positions and refined by use of a riding model with isotropic displacement parameters based on the equivalent isotropic displacement parameter (U_{eq}) of the parent atom. Figures and reports were produced using OLEX2.[S3]

The structure contains one molecule of dichloromethane per molecule of **23**.The structure is in a centrosymmetric space group such that, in four of the molecules in the unit cell C(2), C(3), C(4a) and C(5a) are *R* while C(10a) is *S* and in the other four molecules in the unit cell C(2), C(3), C(4a) and C(5a) are *S* while C(10a) is *R*.

The CIF for the crystal structure of **23** has been deposited with the CCDC and have been given the deposition number CCDC 1523963.

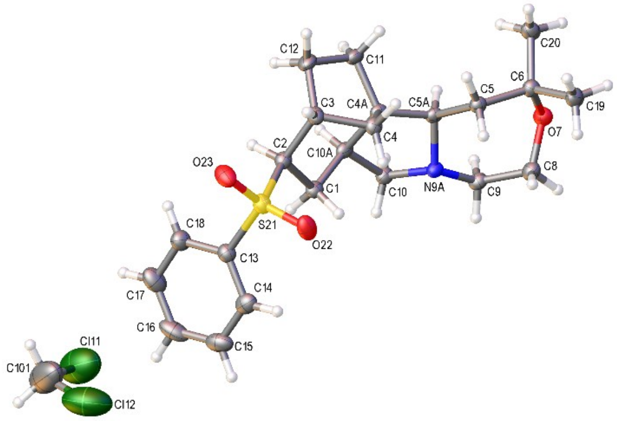

Fig. S1: Crystal structure of **23** with ellipsoids drawn at the 50 % probability level. The structure contains one molecule of dichloromethane per molecule of **23**.

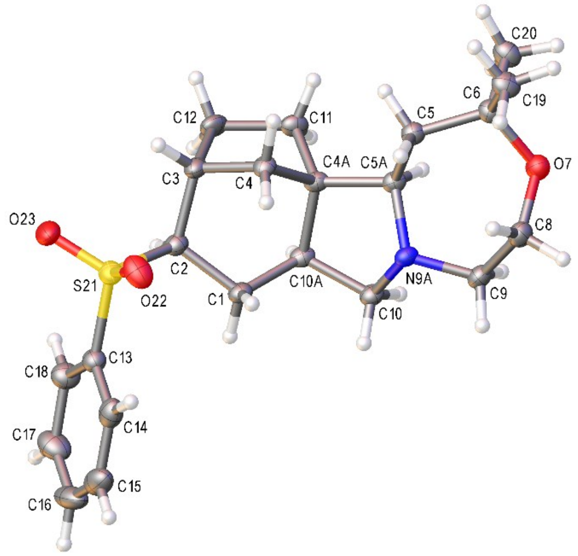

Fig. S2: Crystal structure of **23** with ellipsoids drawn at the 50 % probability level. The structure contains one molecule of dichloromethane per molecule of **23** which has been omitted for clarity.

[S1] CrysAlisPro, Agilent Technologies, Version 1.171.36.28, **2013**.

[S2] G. M. Sheldrick, *Acta Cryst.* **2008**, *A64*, 112-122.

[S3] Dolomanov, O. V.; Bourhis, L. J.; Gildea, R. J.; Howard J. A. K.; Puschmann, H. *J. Appl. Crystallogr.* **2009**, *42*, 339-341.

NMR spectra

^{1}H NMR (400 MHz, $CDCl_3$) and DEPTq (101 MHz, $CDCl_3$) spectra for **8**

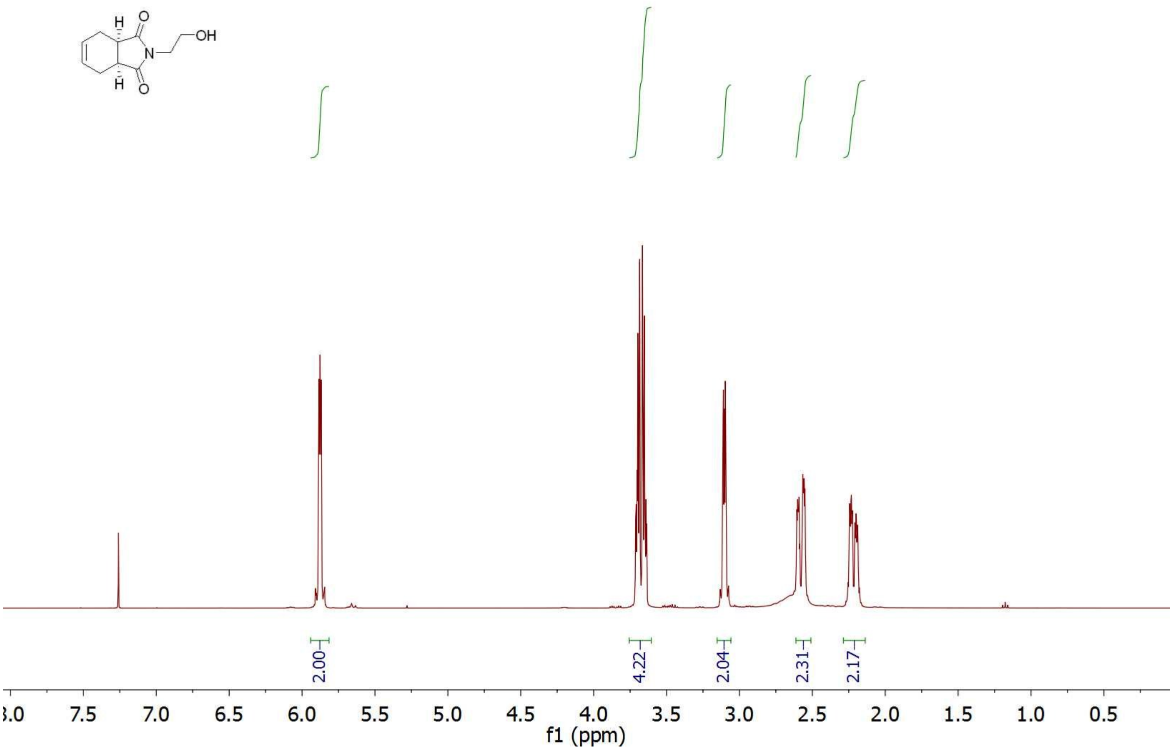

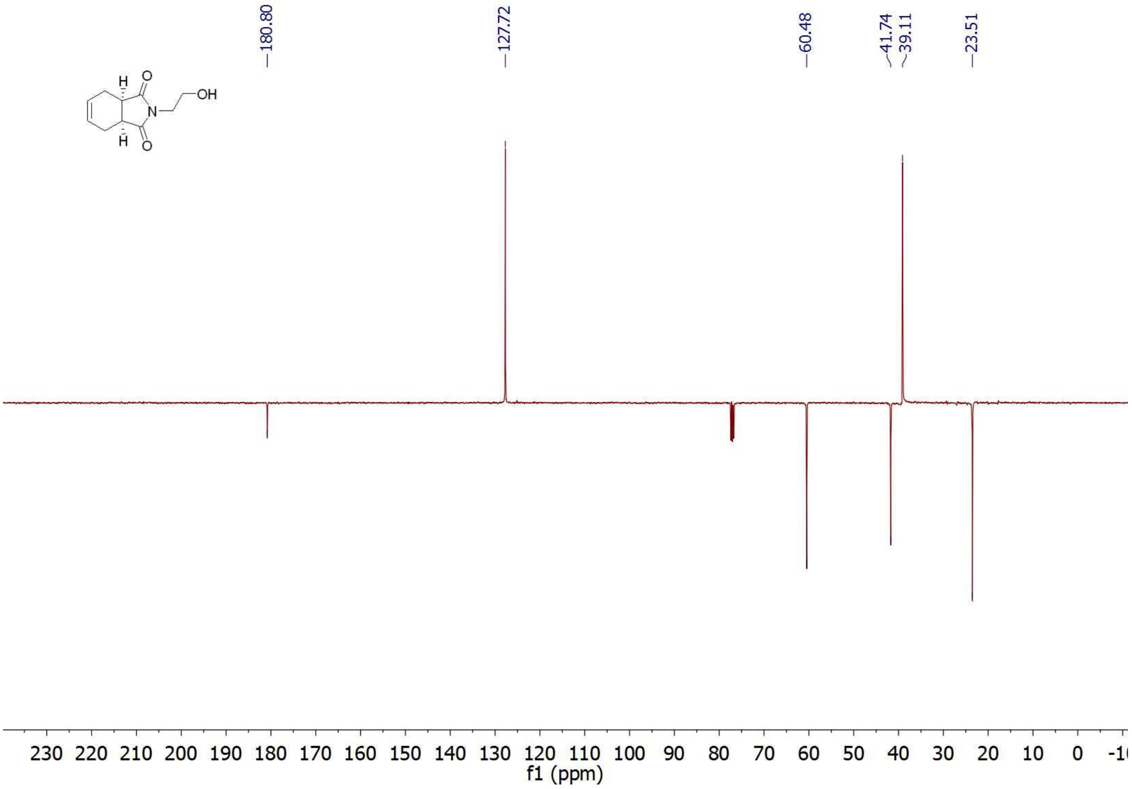

^{1}H NMR (400 MHz, $CDCl_3$) and DEPTq (101 MHz, $CDCl_3$) spectra for **9**

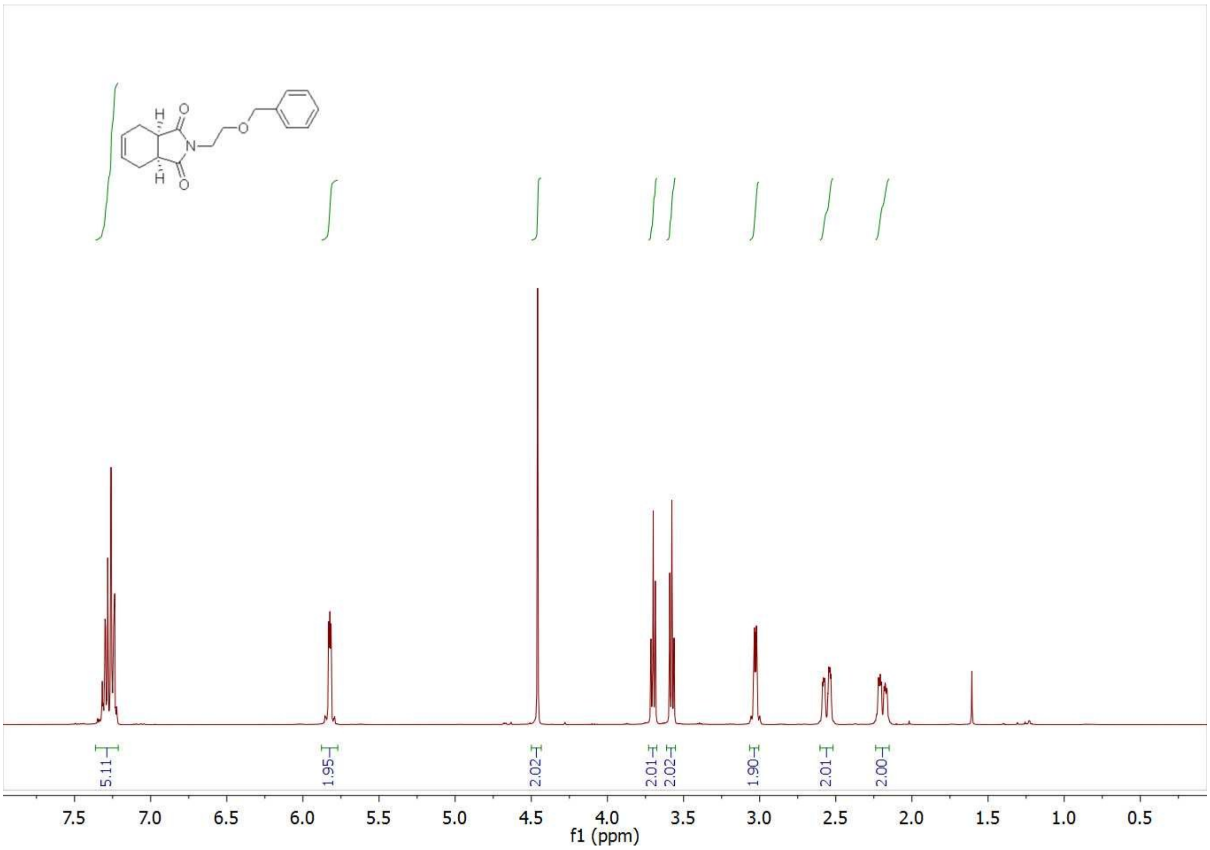

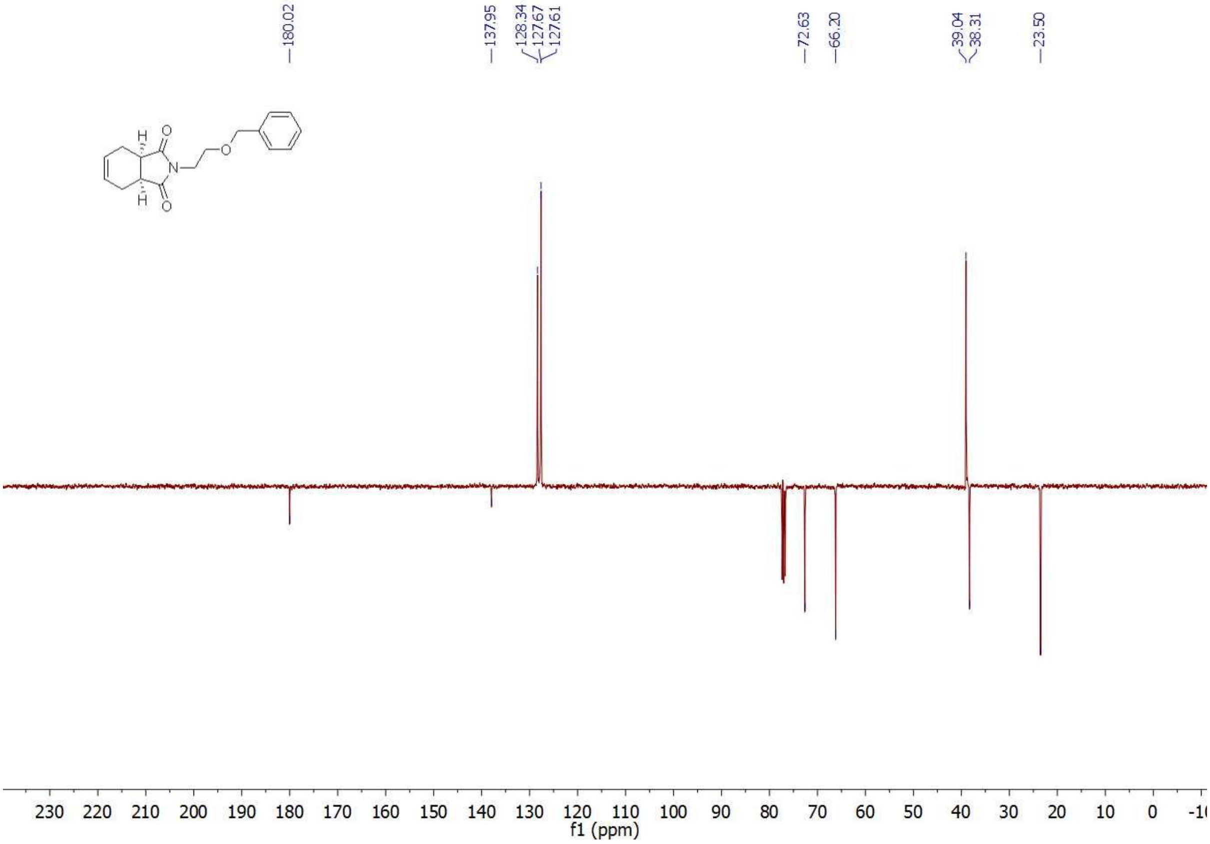

^{1}H NMR (400 MHz, $CDCl_3$) and DEPTq (101 MHz, $CDCl_3$) spectra for **10**

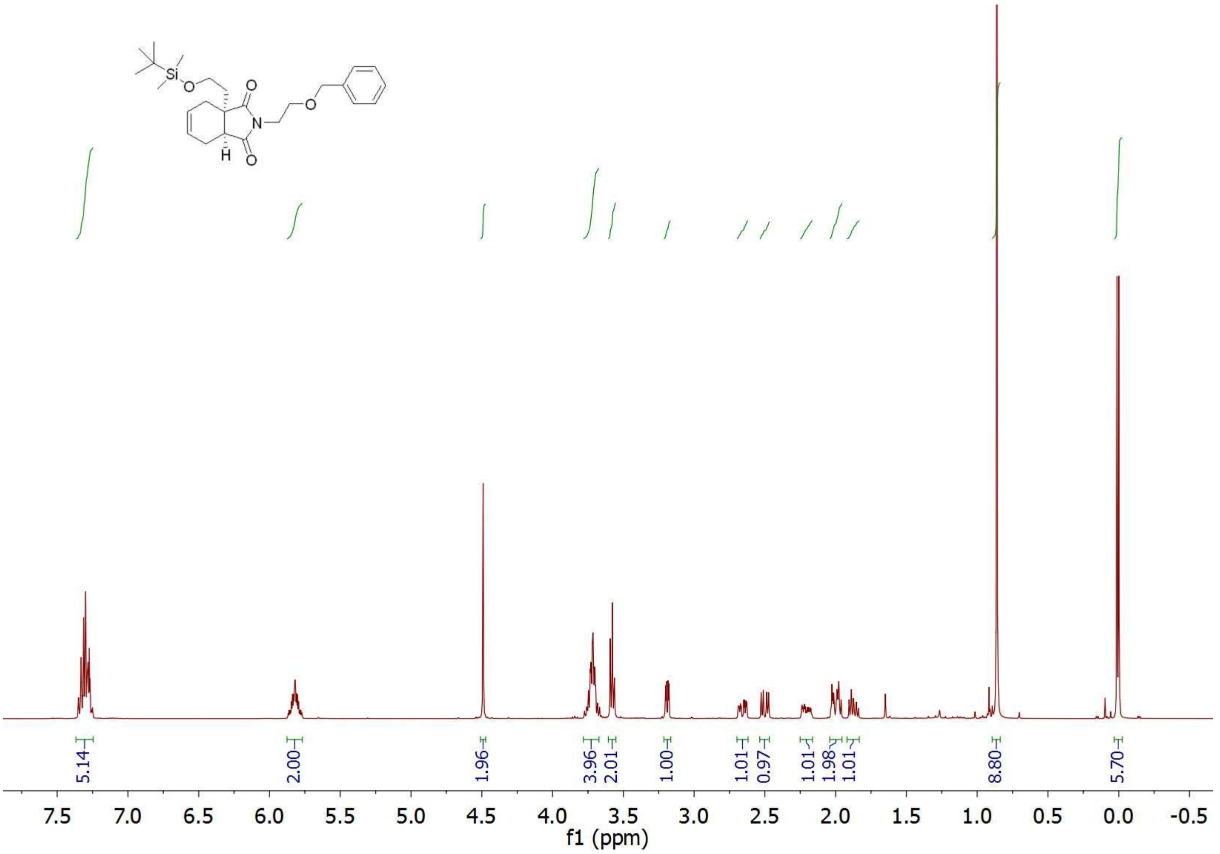

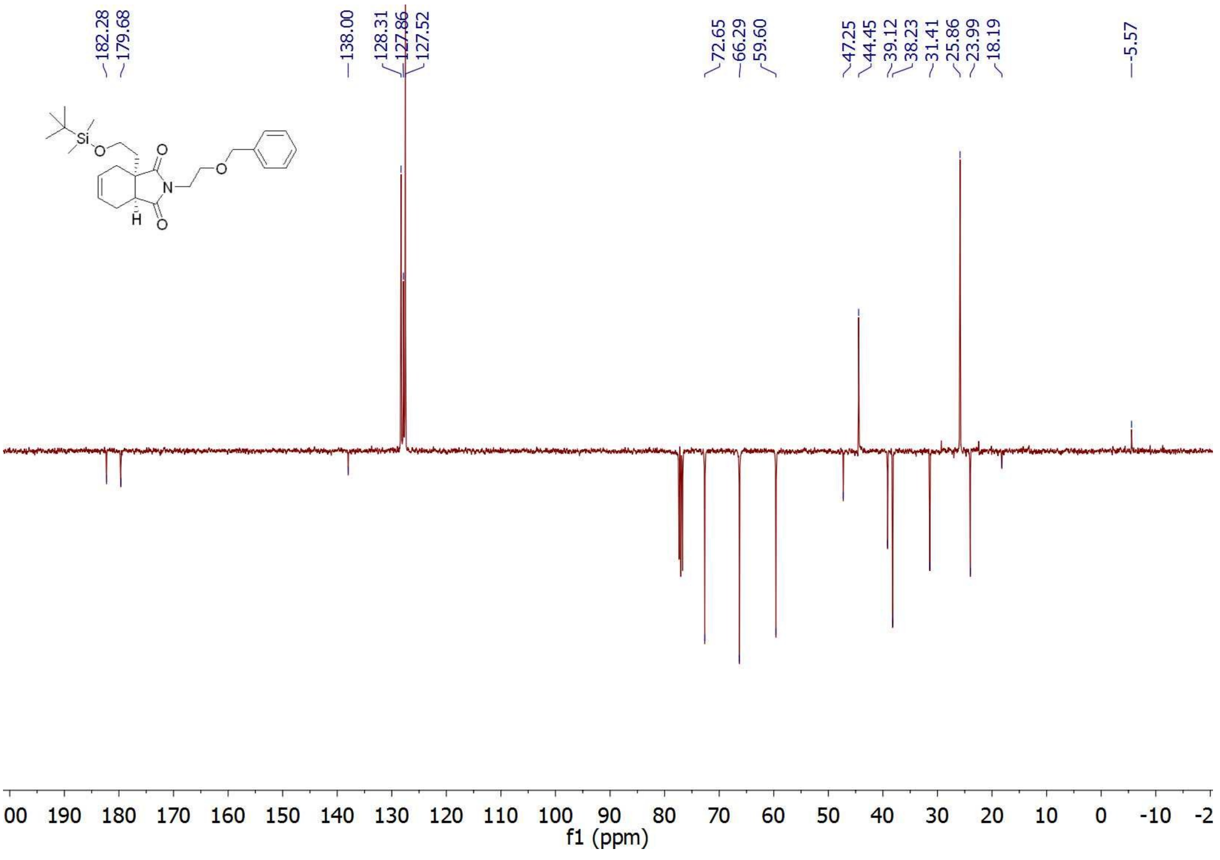

^{1}H NMR (400 MHz, $CDCl_3$) and DEPTq (101 MHz, $CDCl_3$) spectra for **11**

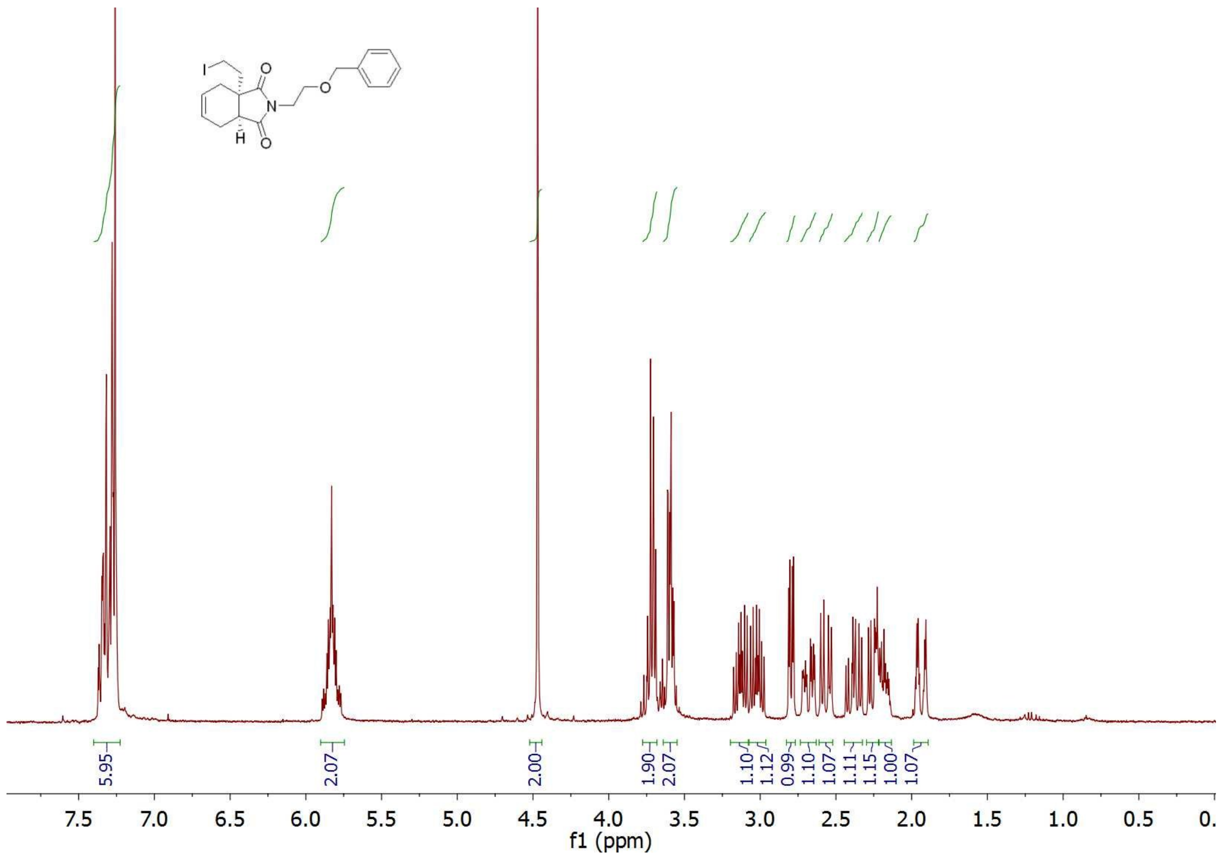

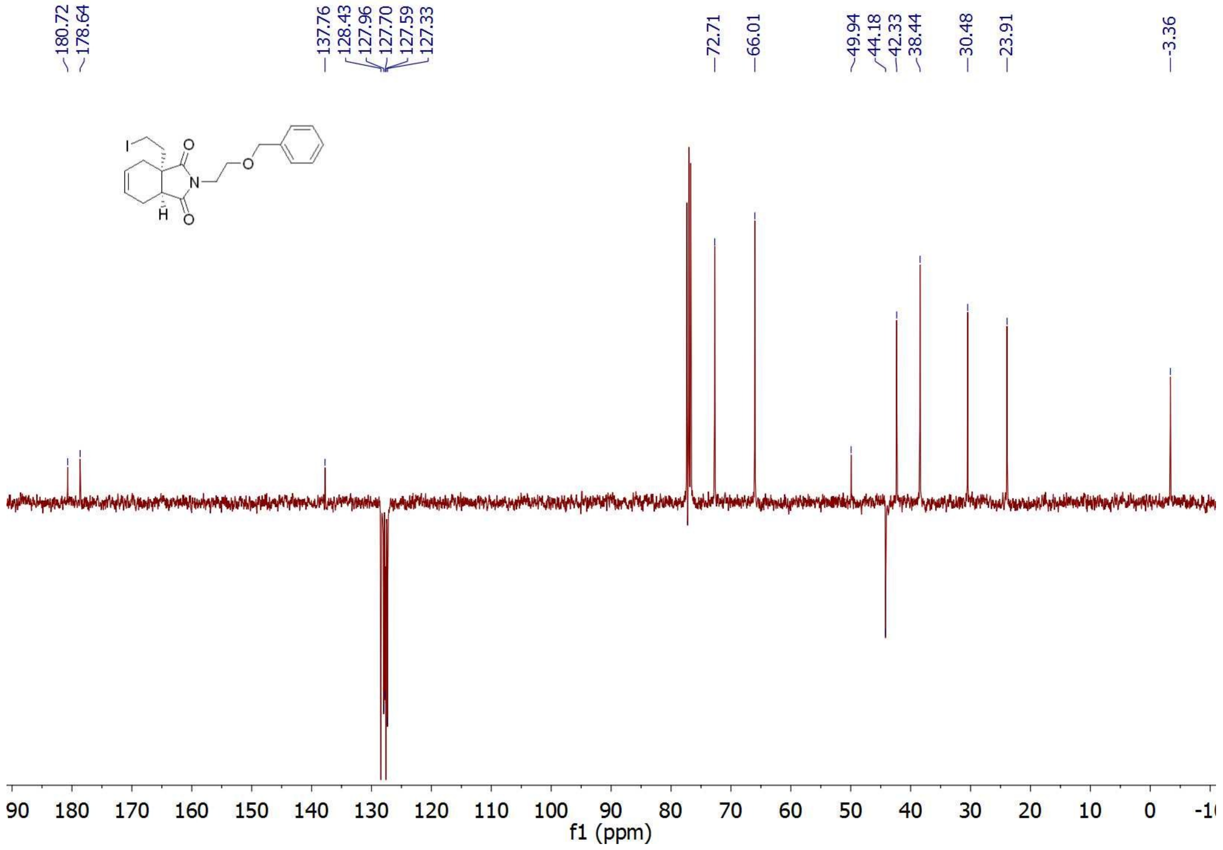

^{1}H NMR (400 MHz, $CDCl_3$) and DEPTq (101 MHz, $CDCl_3$) spectra for **12**

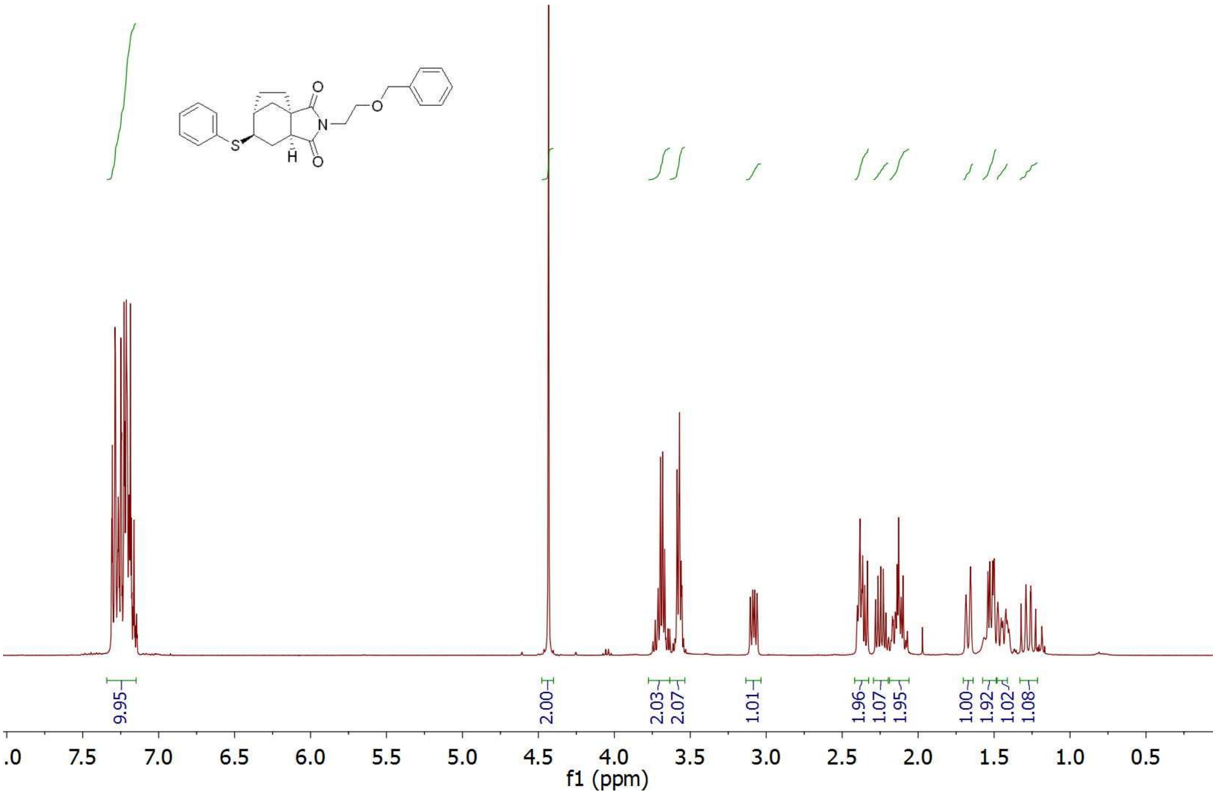

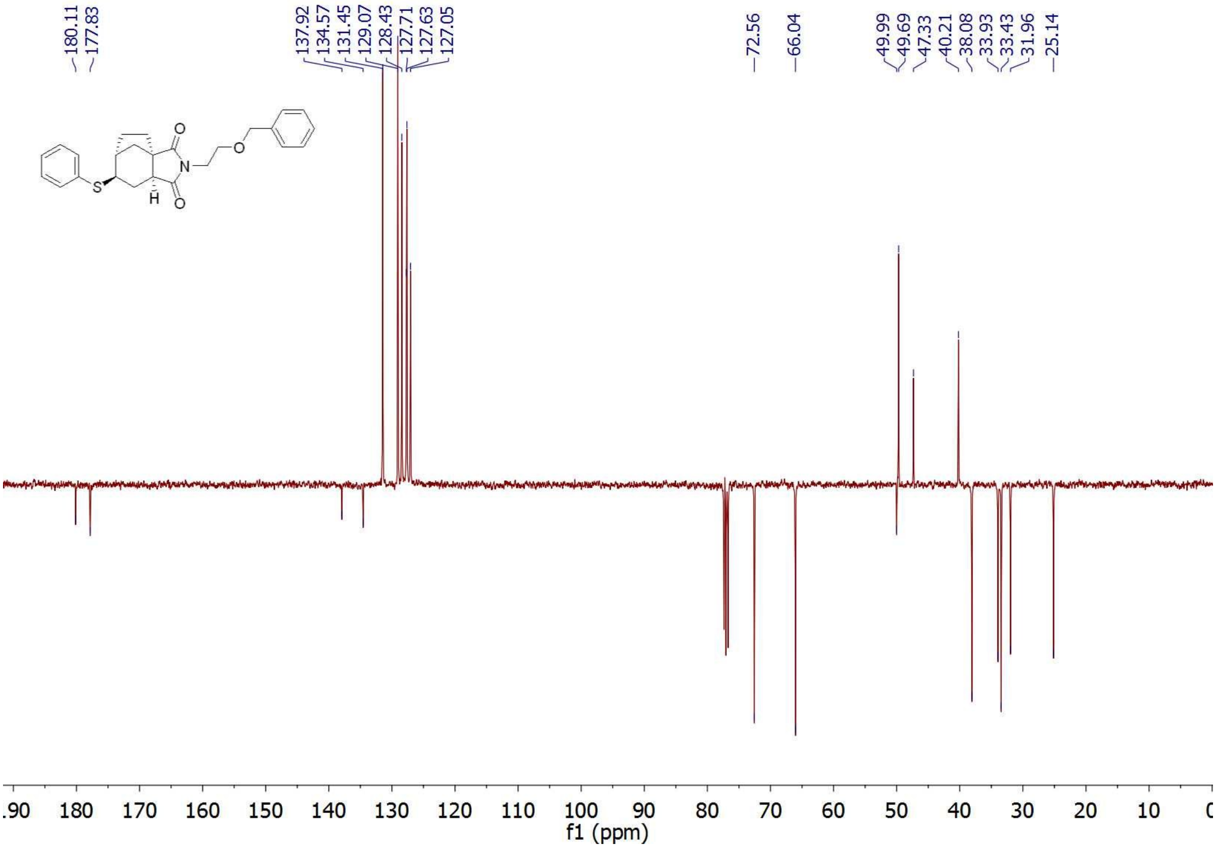

^{1}H NMR (400 MHz, $CDCl_3$) and DEPTq (101 MHz, $CDCl_3$) spectra for **13**

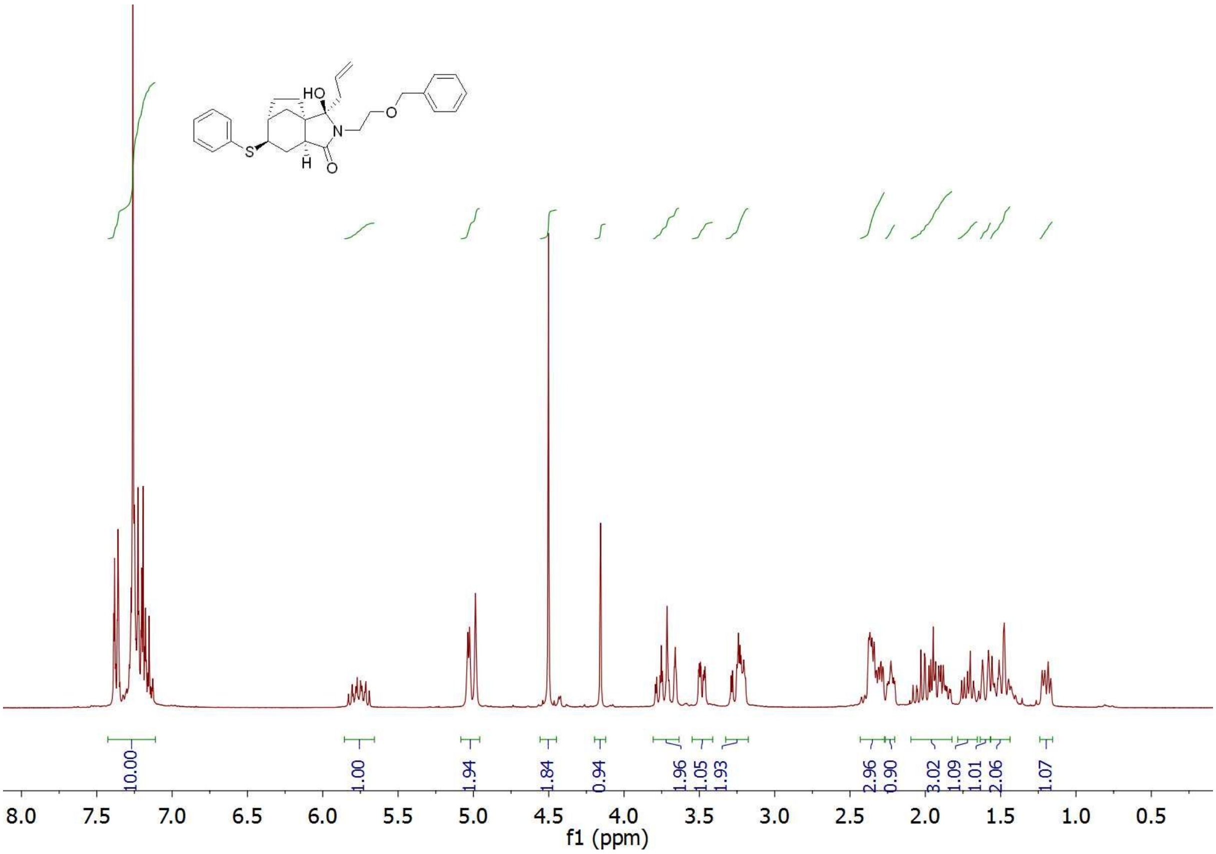

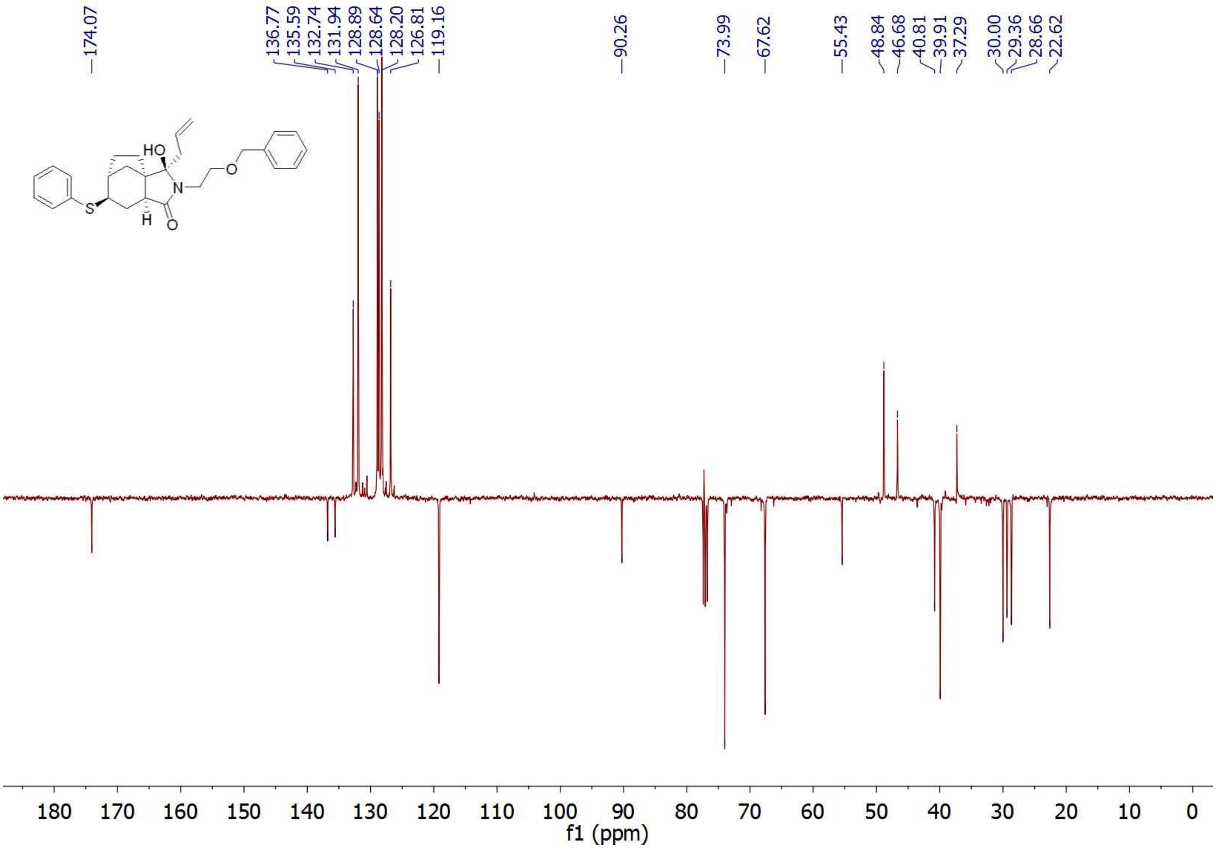

^1H NMR (400 MHz, $CDCl_3$) and DEPTq (101 MHz, $CDCl_3$) spectra for **14**

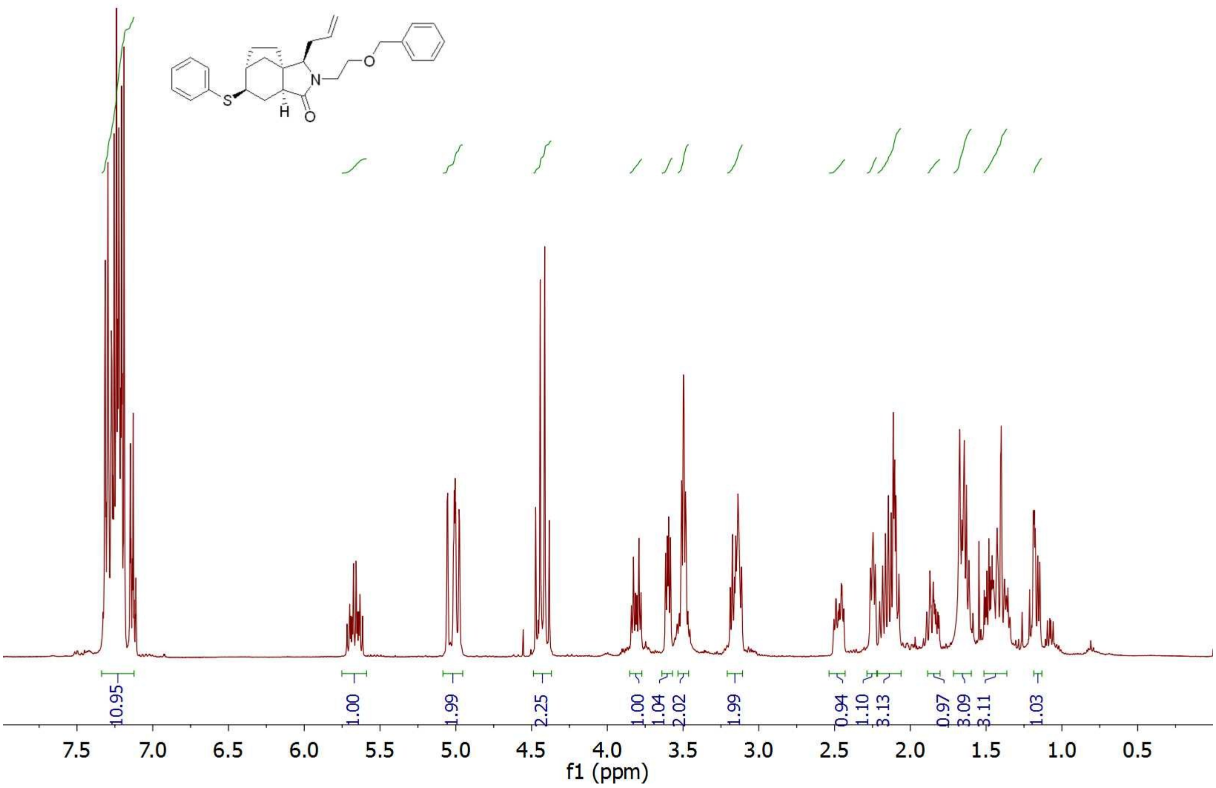

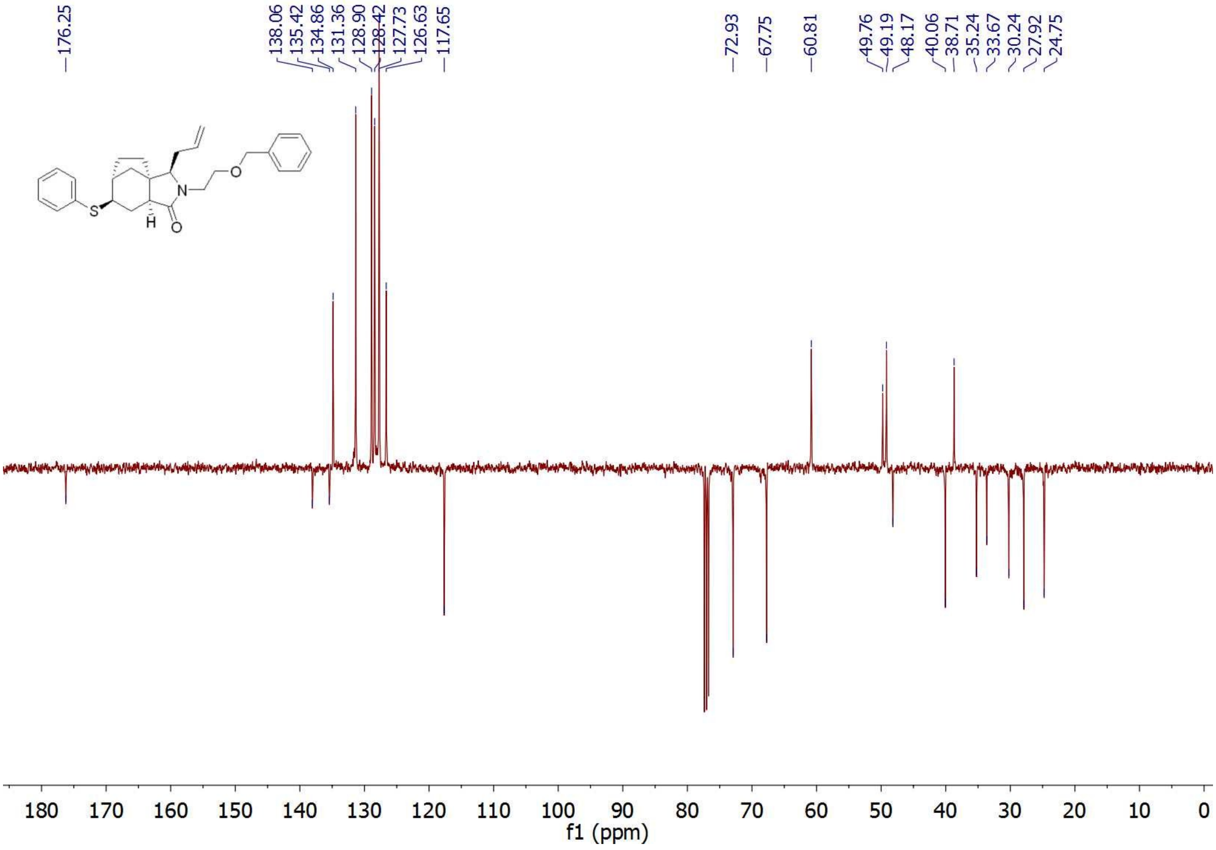

^1H NMR (400 MHz, $CDCl_3$) and DEPTq (101 MHz, $CDCl_3$) spectra for **15**

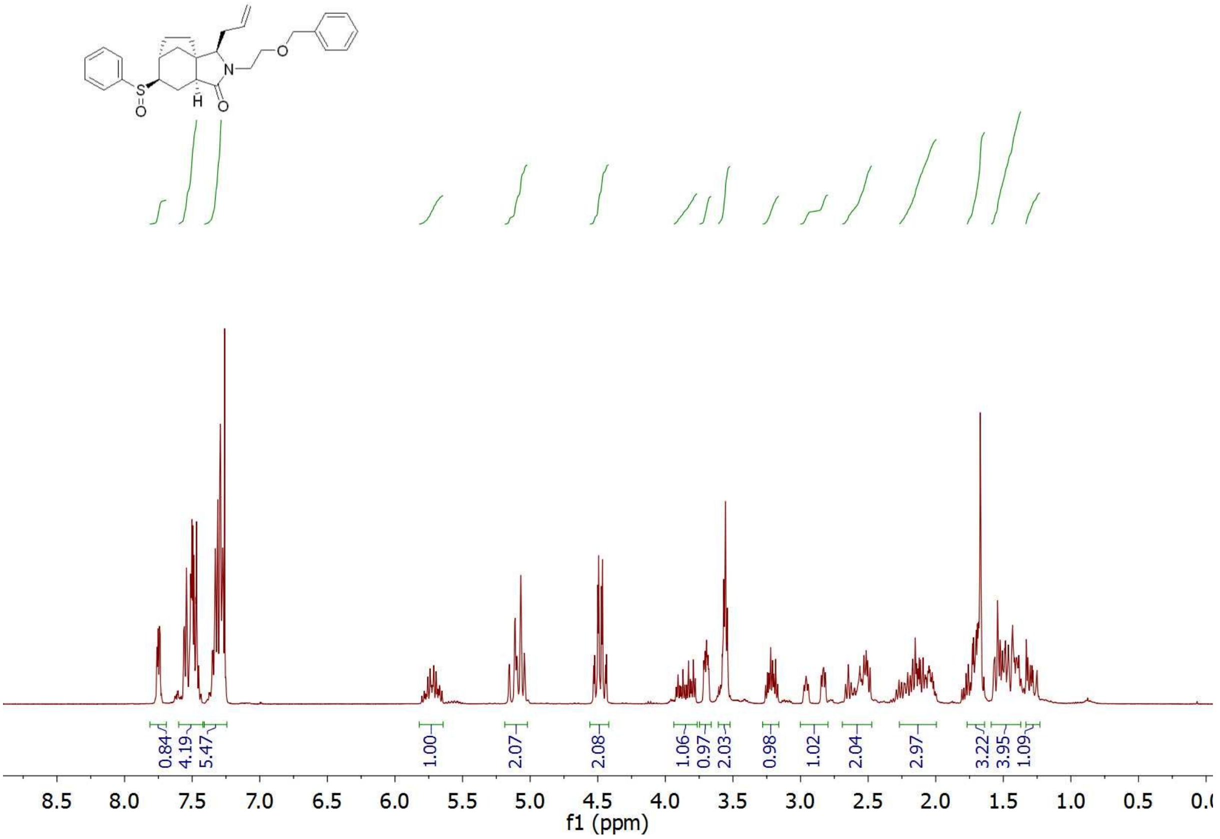

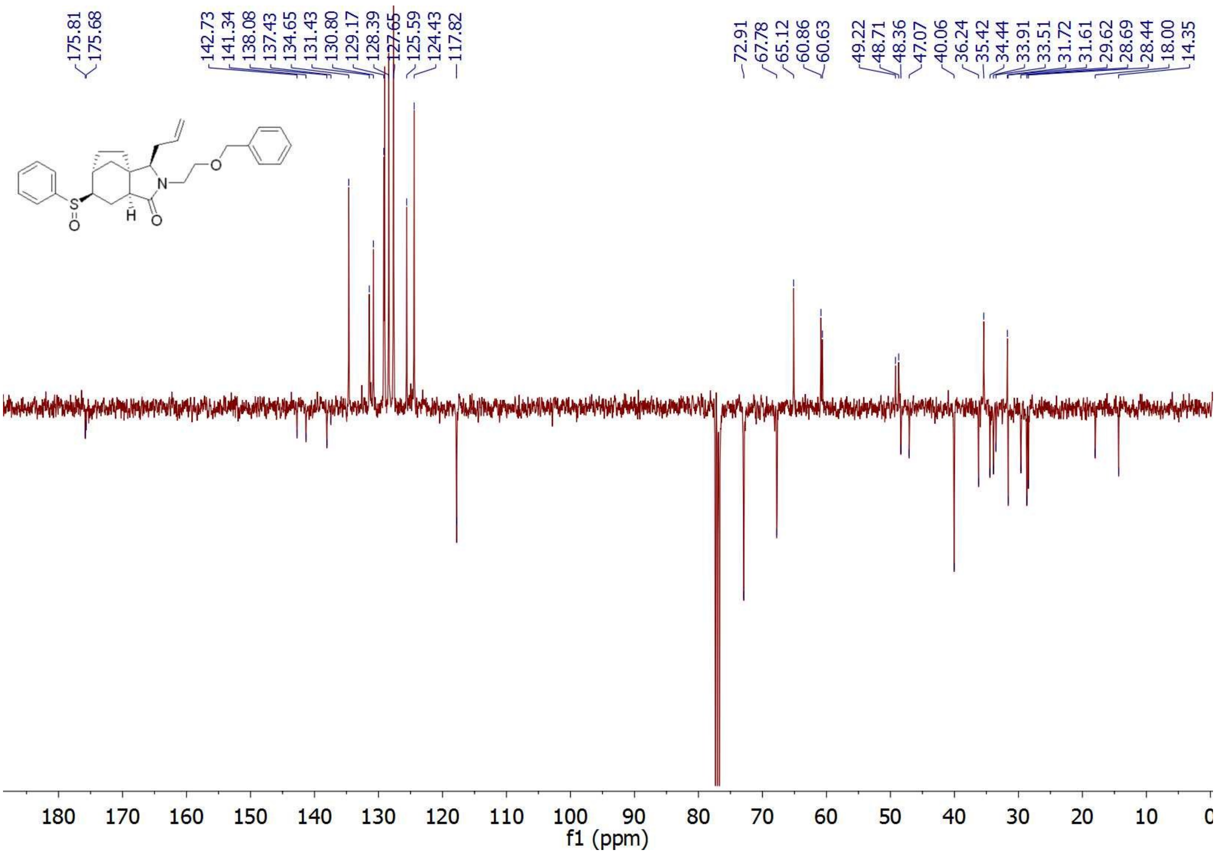

^1H NMR (400 MHz, $CDCl_3$) and DEPTq (101 MHz, $CDCl_3$) spectra for **16**

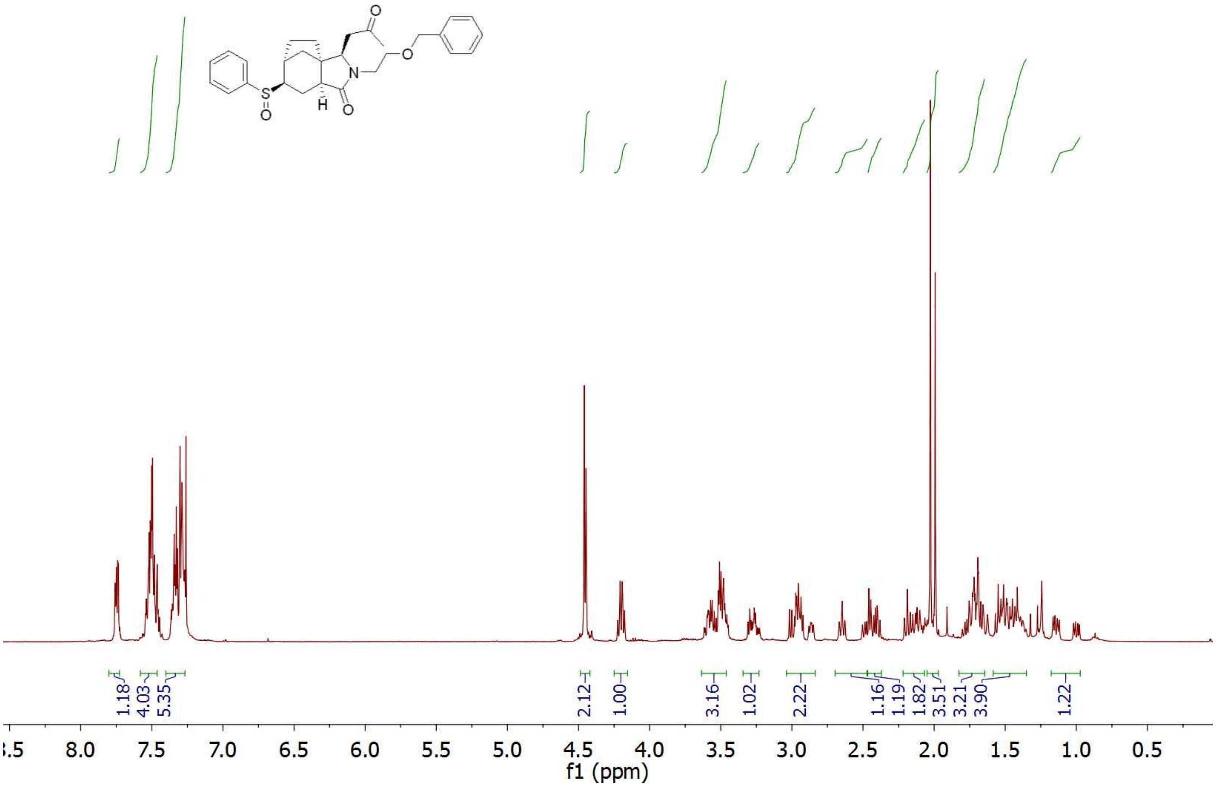

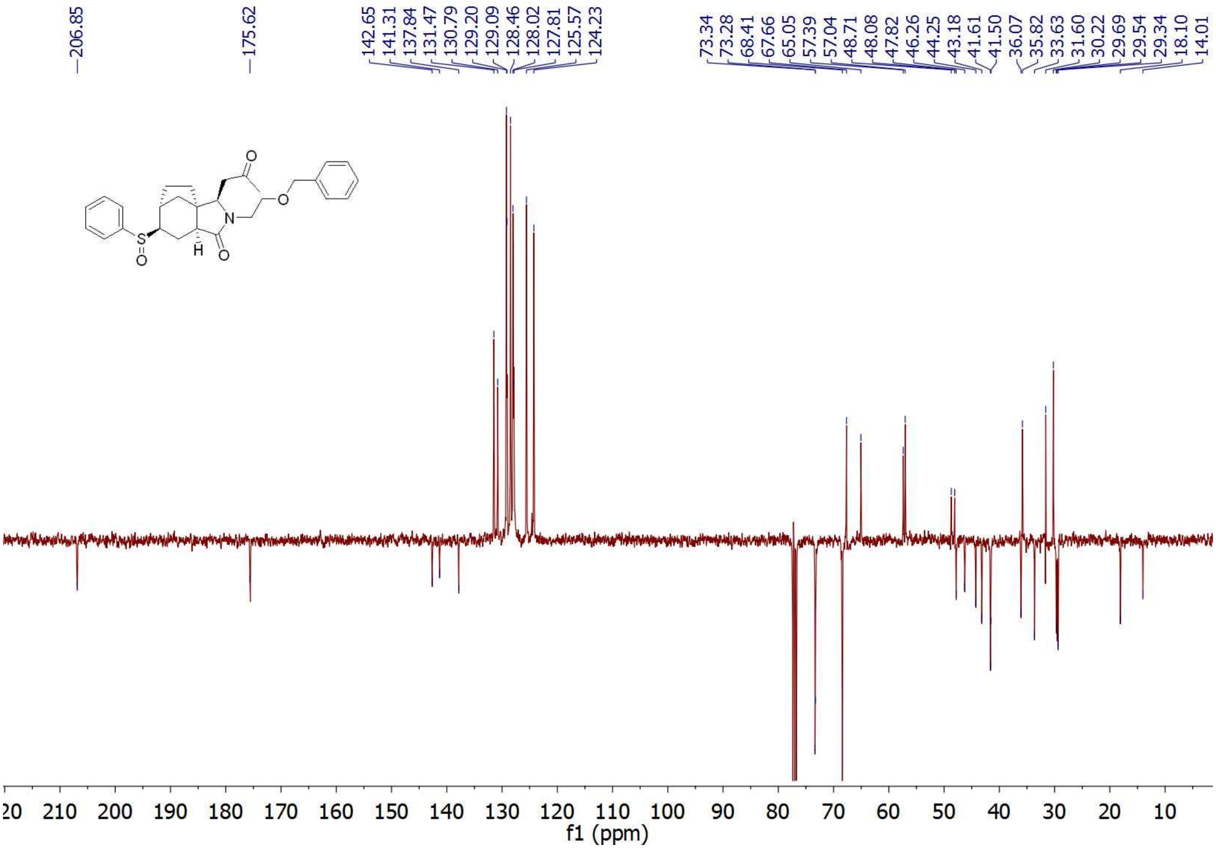

^1H NMR (400 MHz, $CDCl_3$) and DEPTq (101 MHz, $CDCl_3$) spectra for **17**

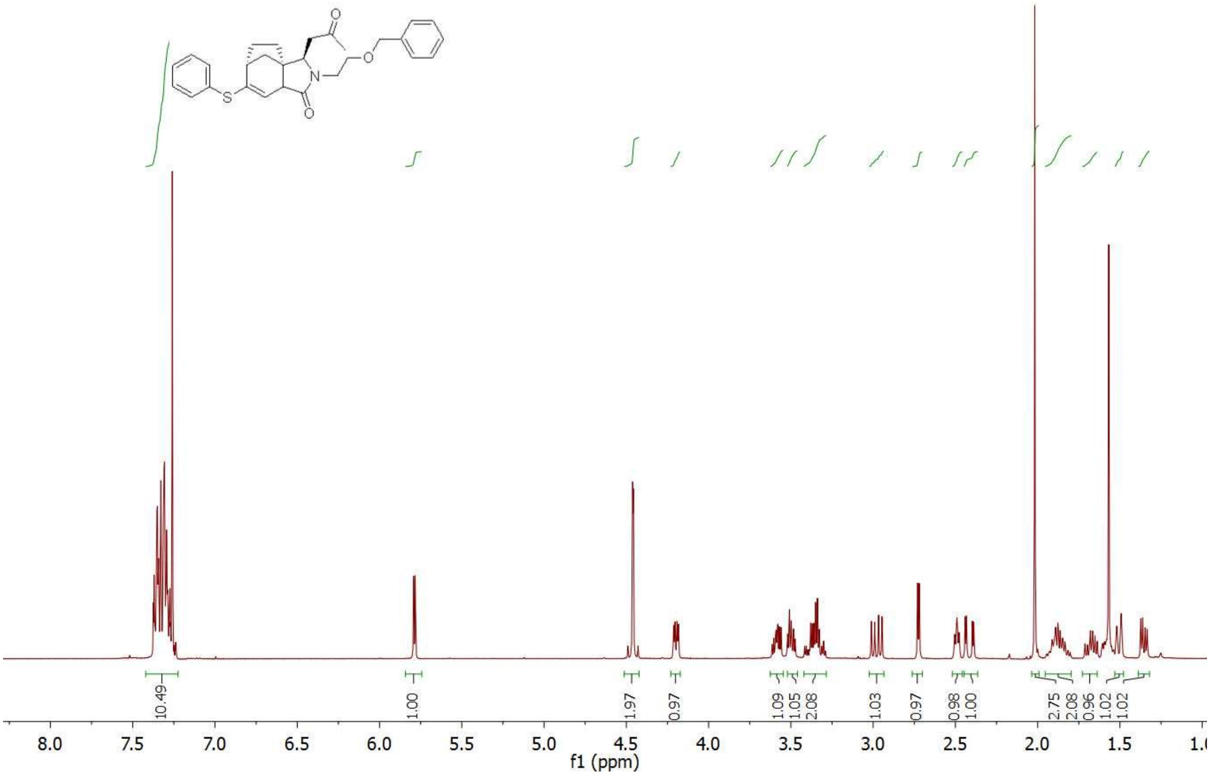

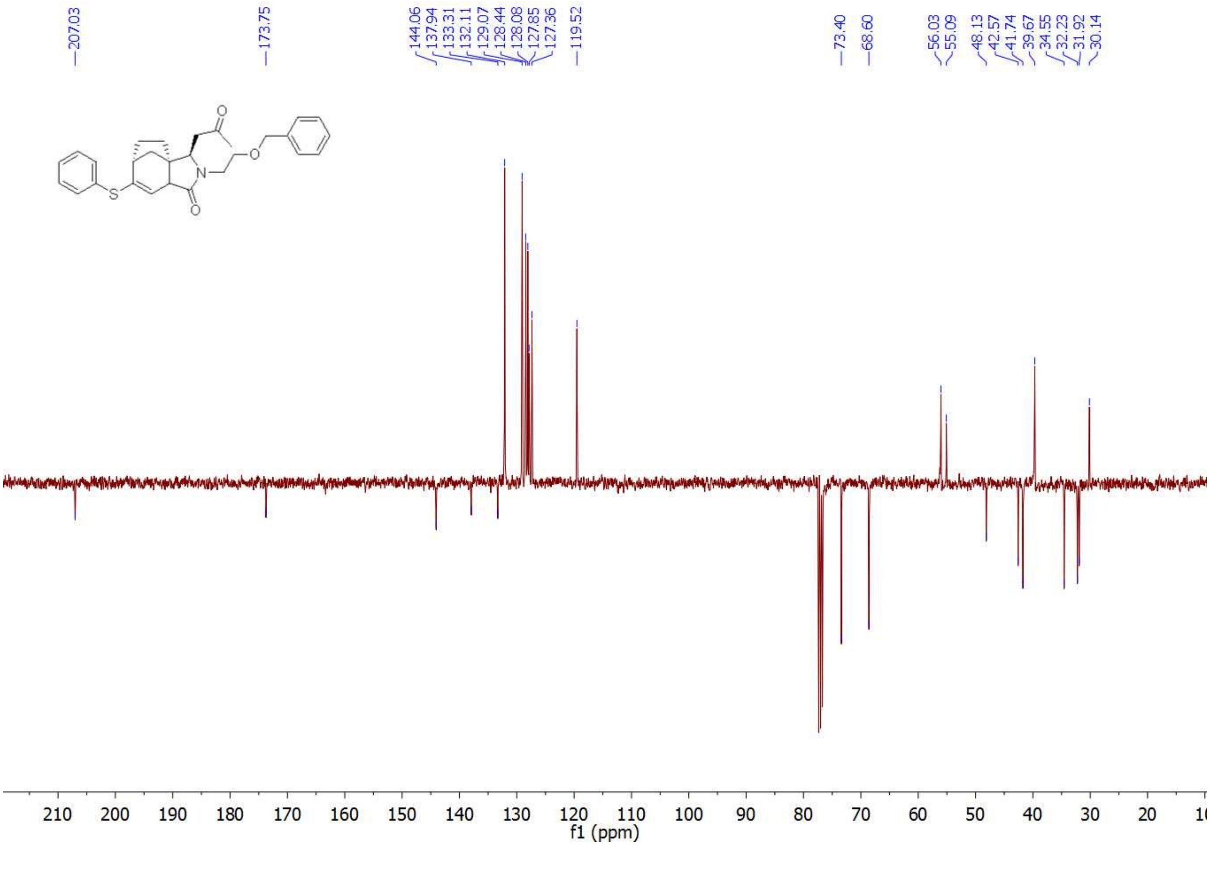

^{1}H NMR (400 MHz, $CDCl_3$) and DEPTq (101 MHz, $CDCl_3$) spectra for **18**

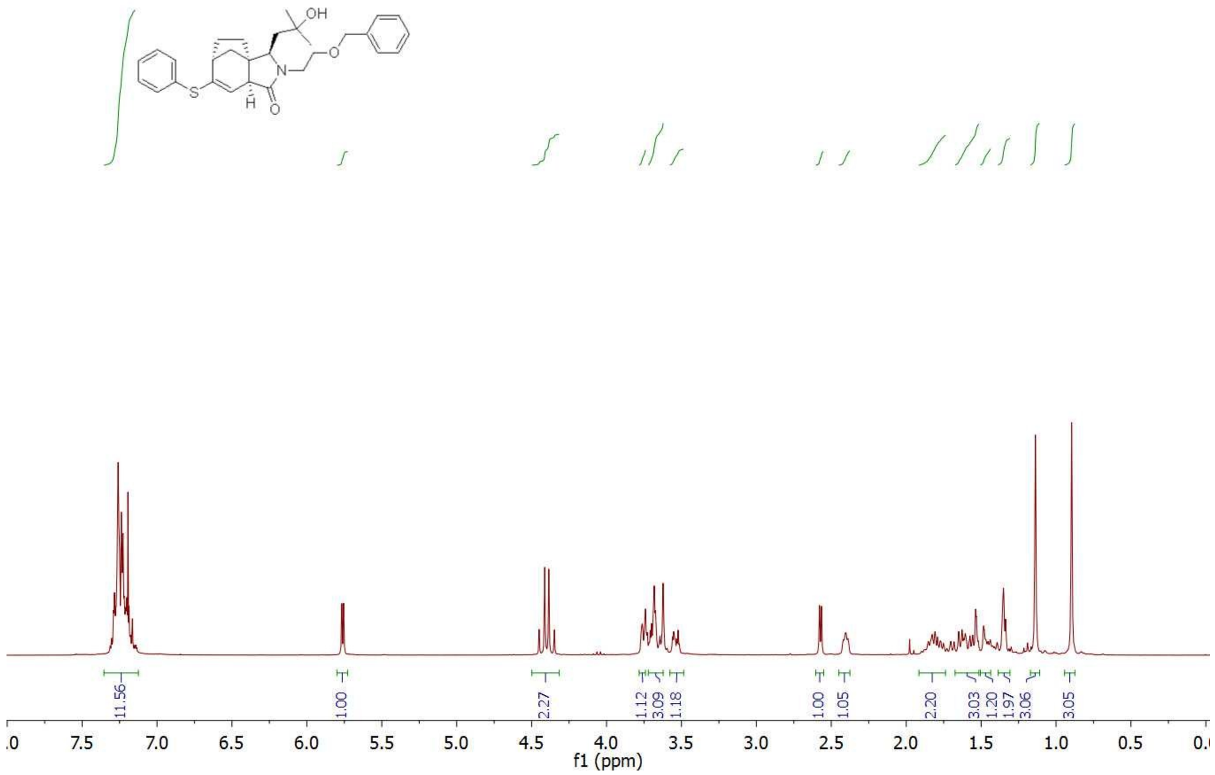

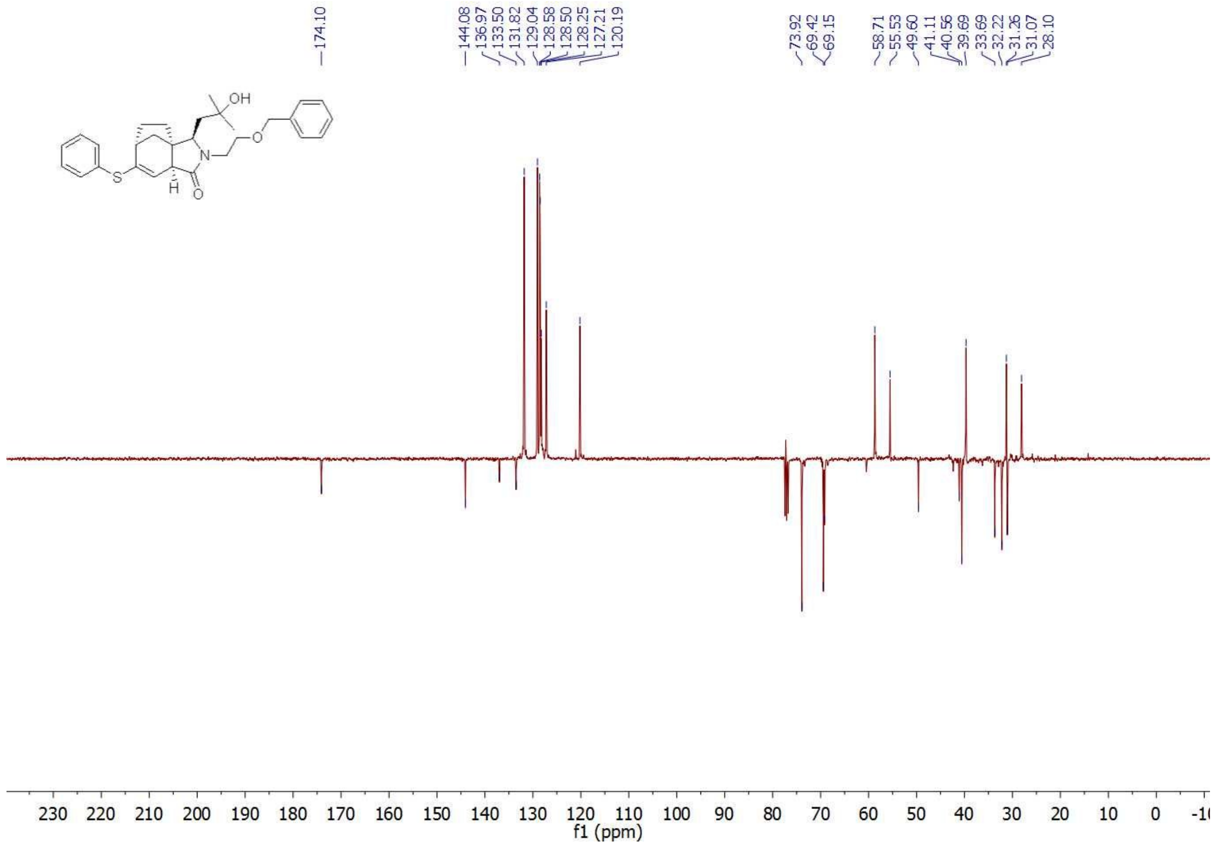

^1H NMR (400 MHz, $CDCl_3$) and DEPTq (101 MHz, $CDCl_3$) spectra for **19**

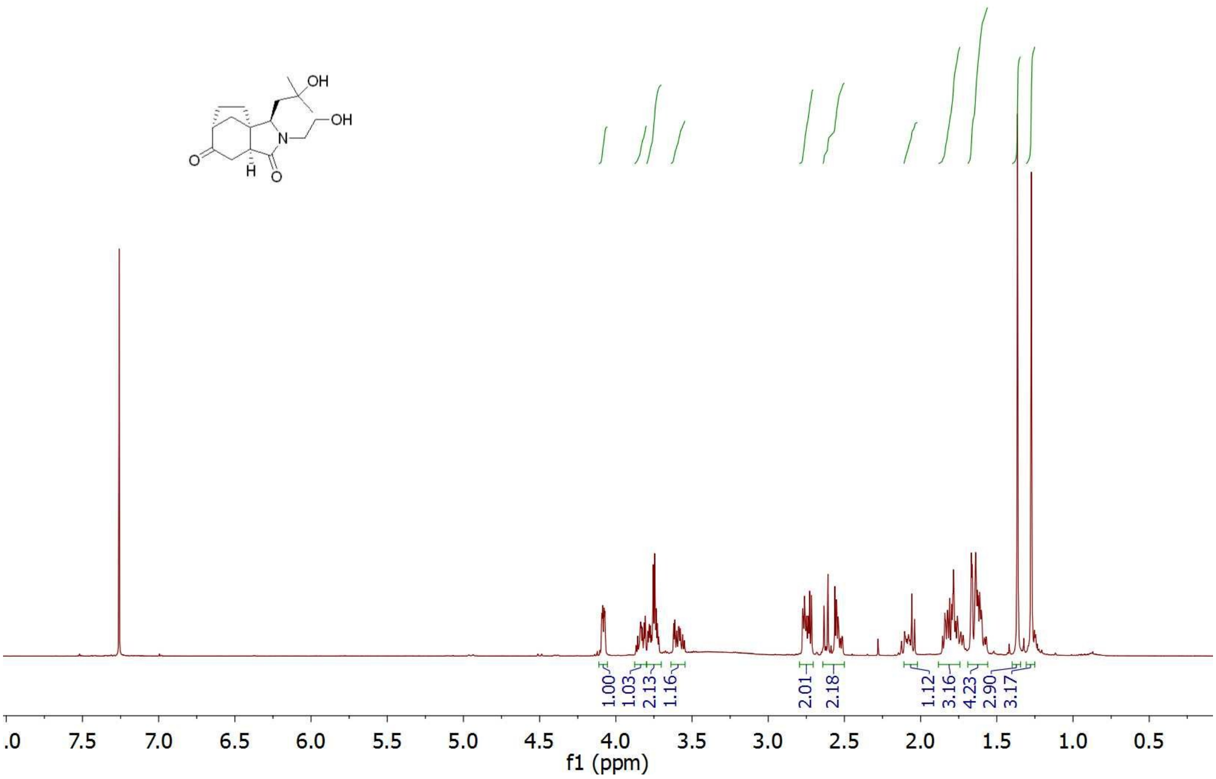

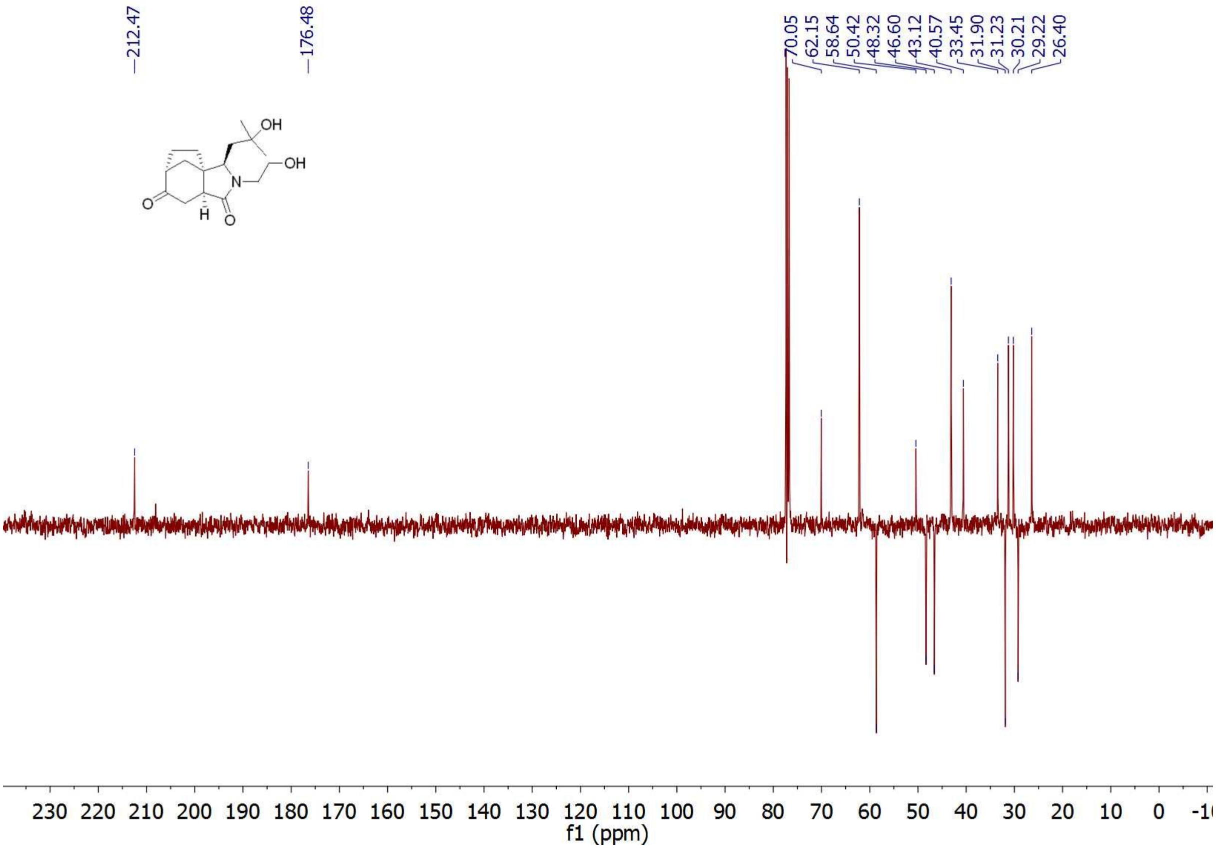

^{1}H NMR (400 MHz, $CDCl_3$) and DEPTq (101 MHz, $CDCl_3$) spectra for **20**

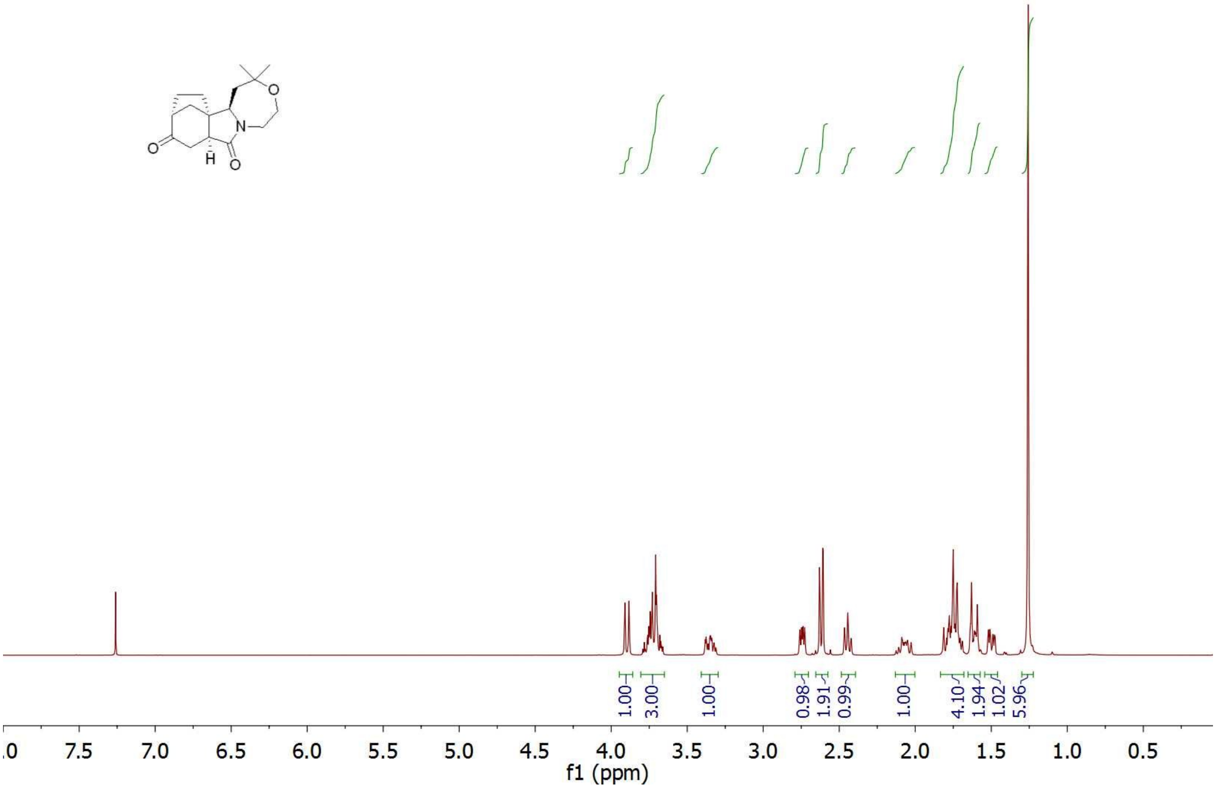

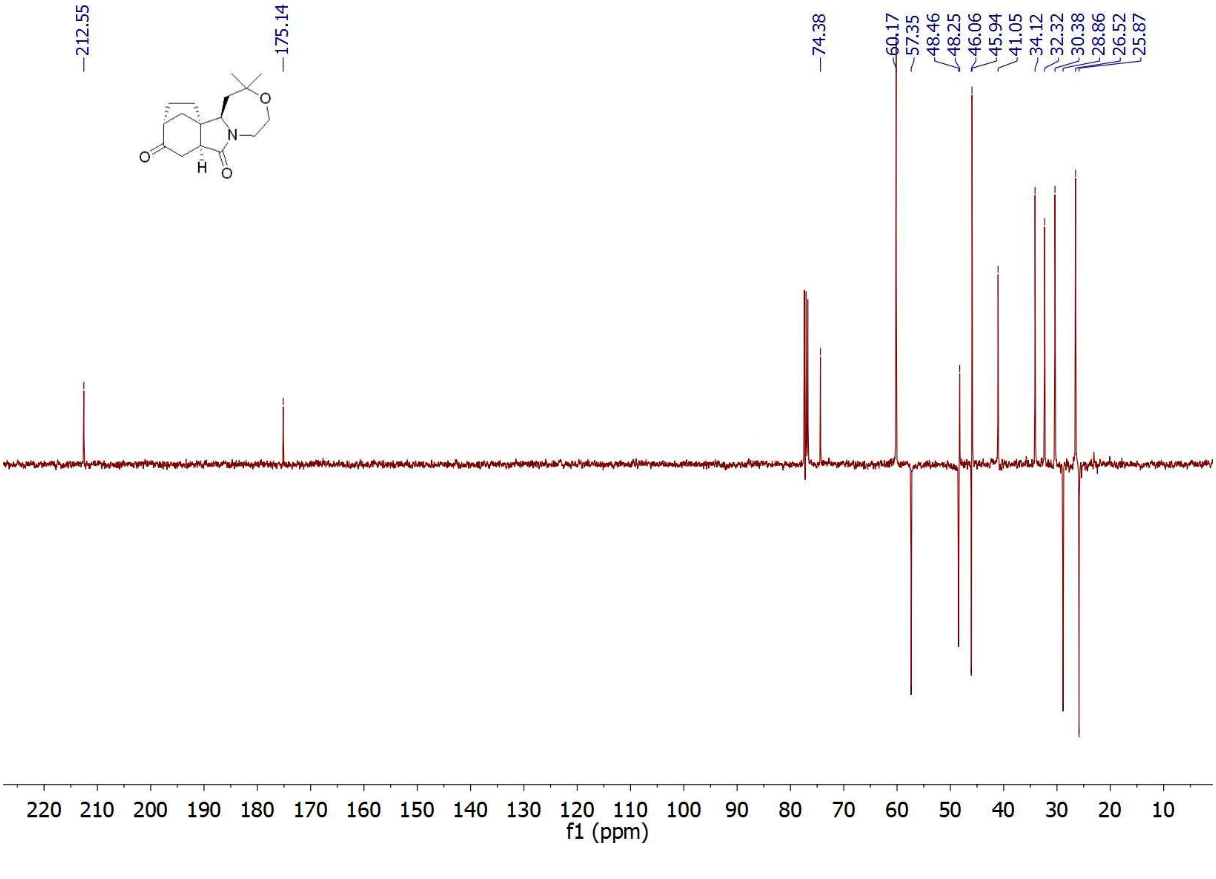

^{1}H NMR (400 MHz, $CDCl_3$) and DEPTq (101 MHz, $CDCl_3$) spectra for **21**

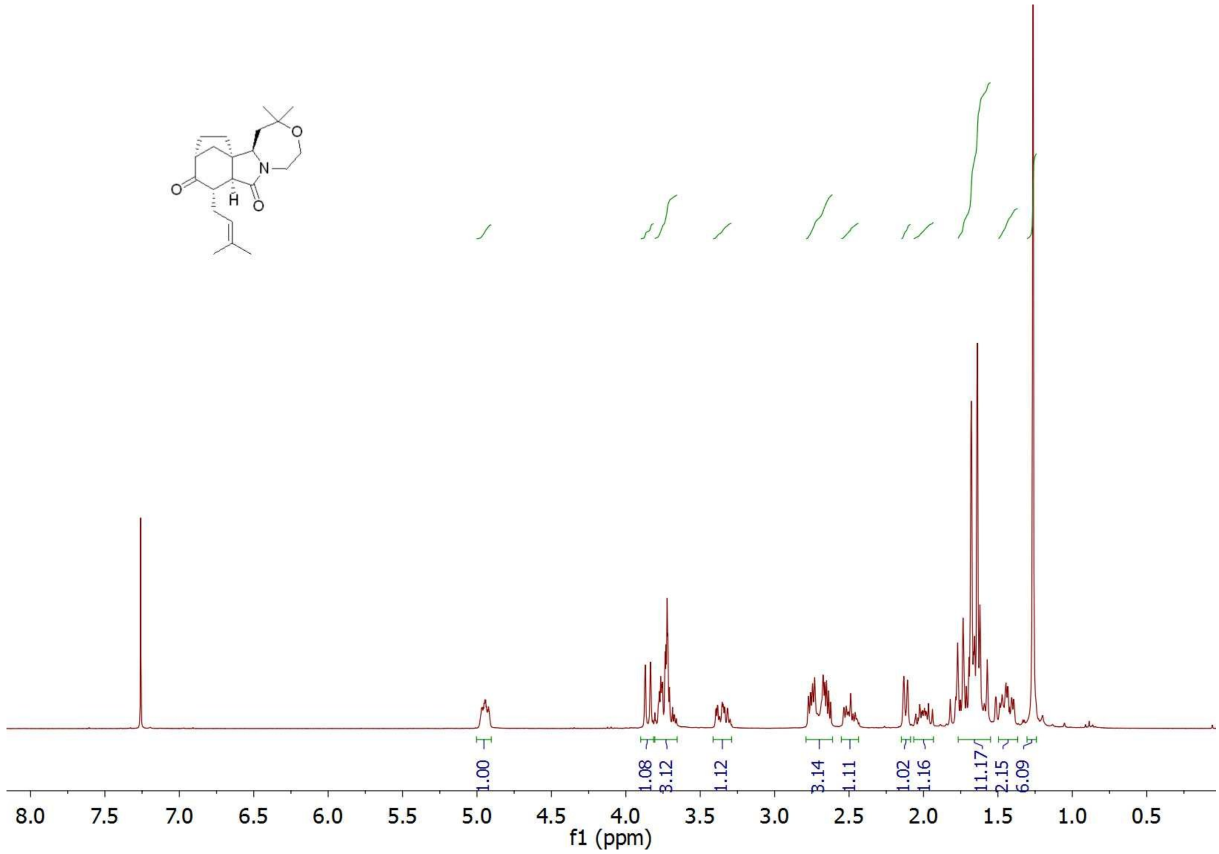

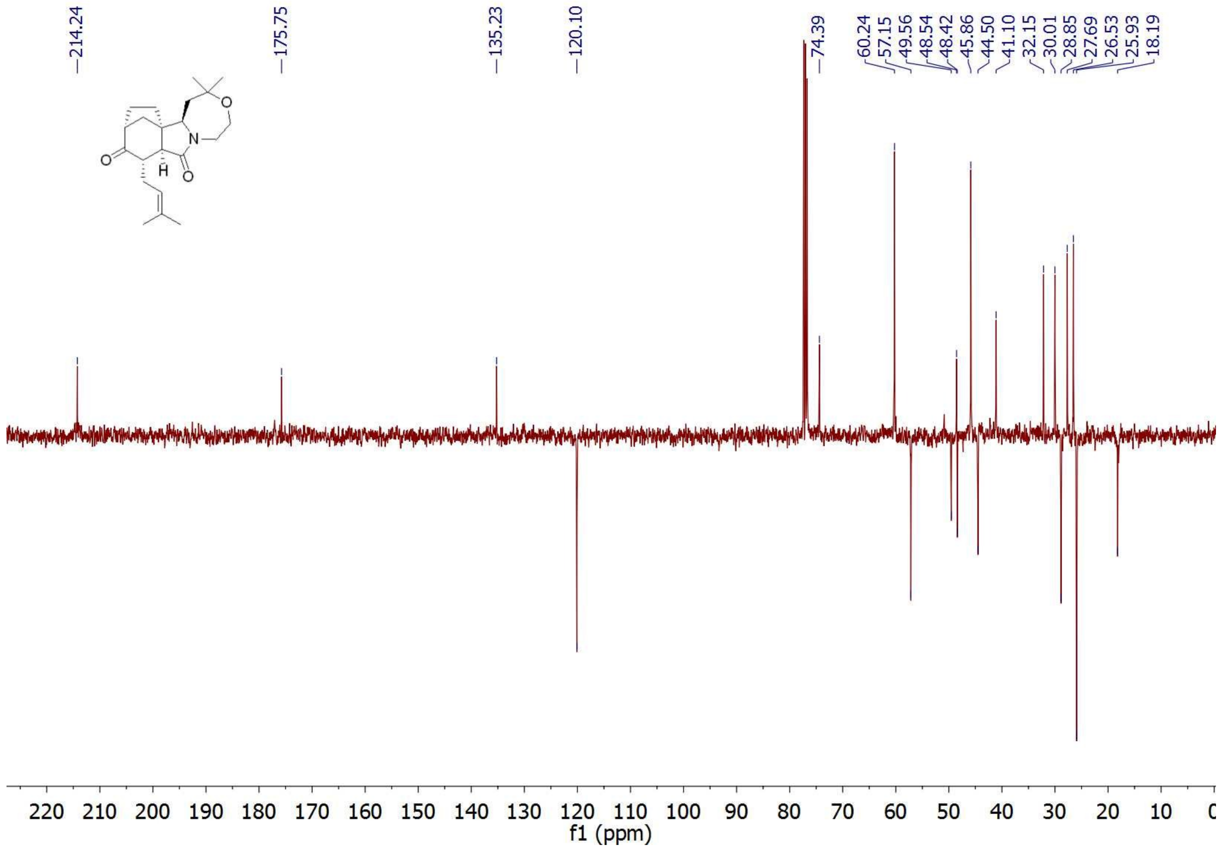

^{1}H NMR (400 MHz, $CDCl_3$) and DEPTq (101 MHz, $CDCl_3$) spectra for **22**

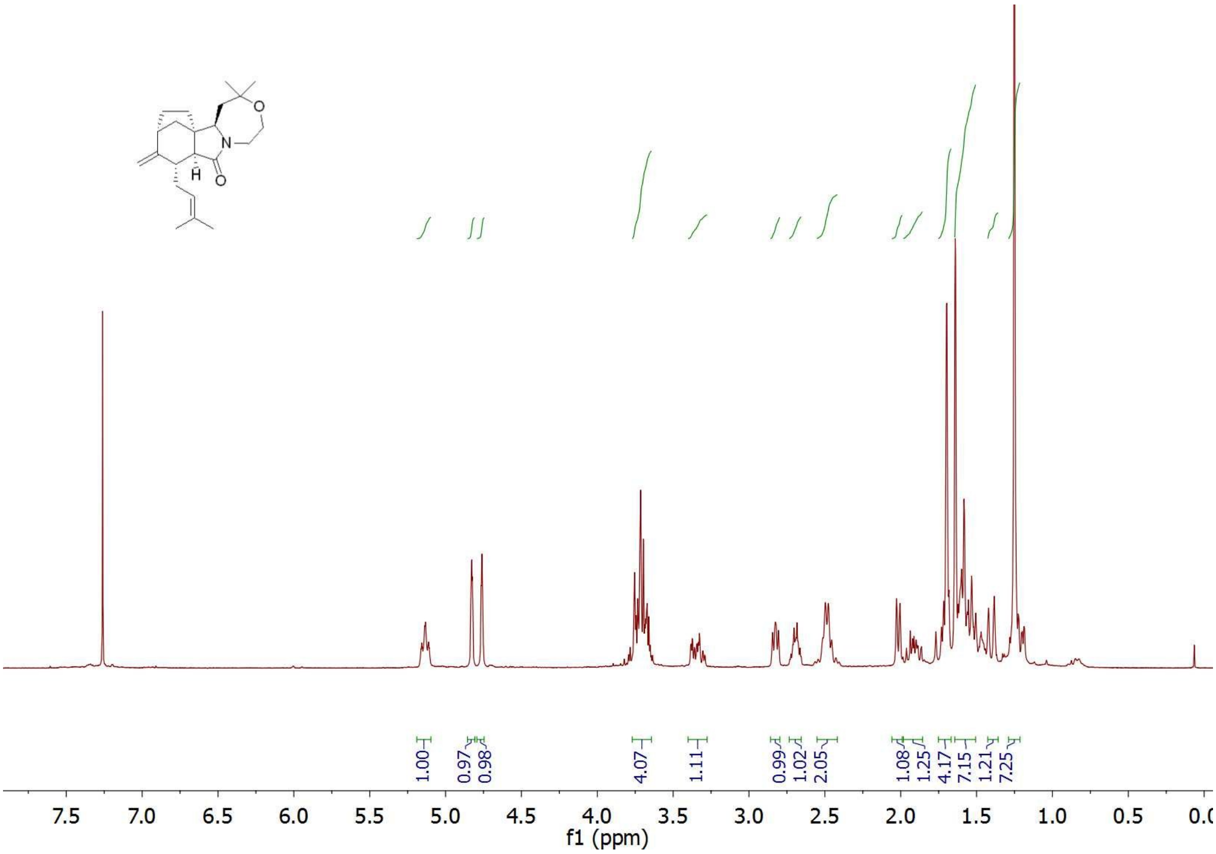

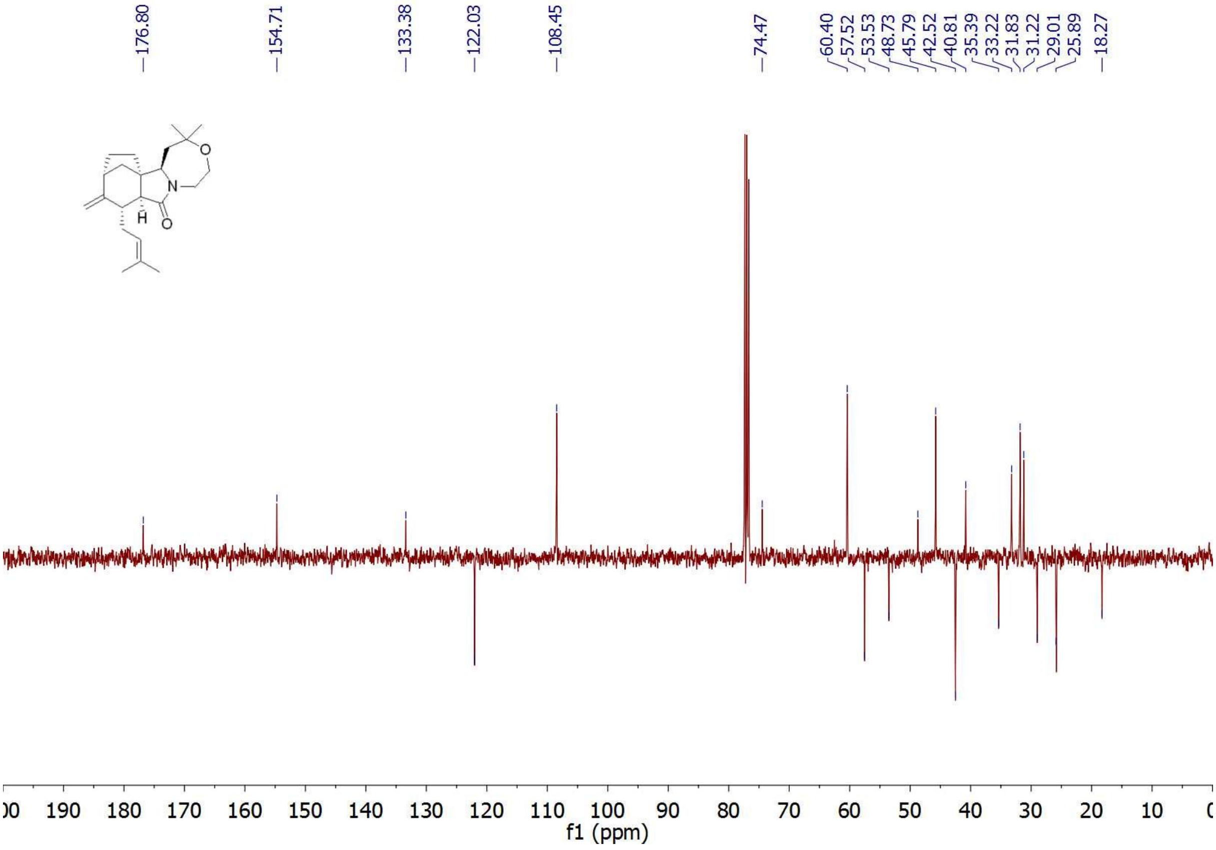

^{1}H NMR (400 MHz, $CDCl_3$) and ^{13}C NMR (101 MHz, $CDCl_3$) spectra for **1**

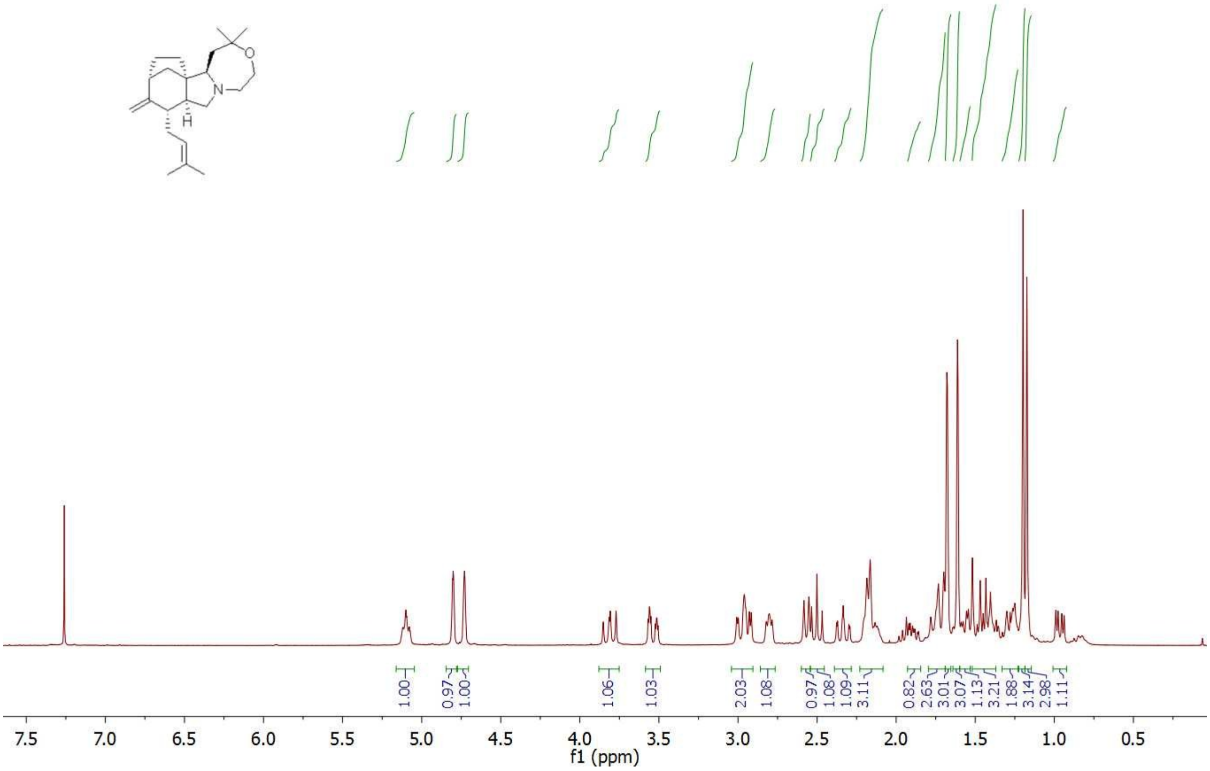

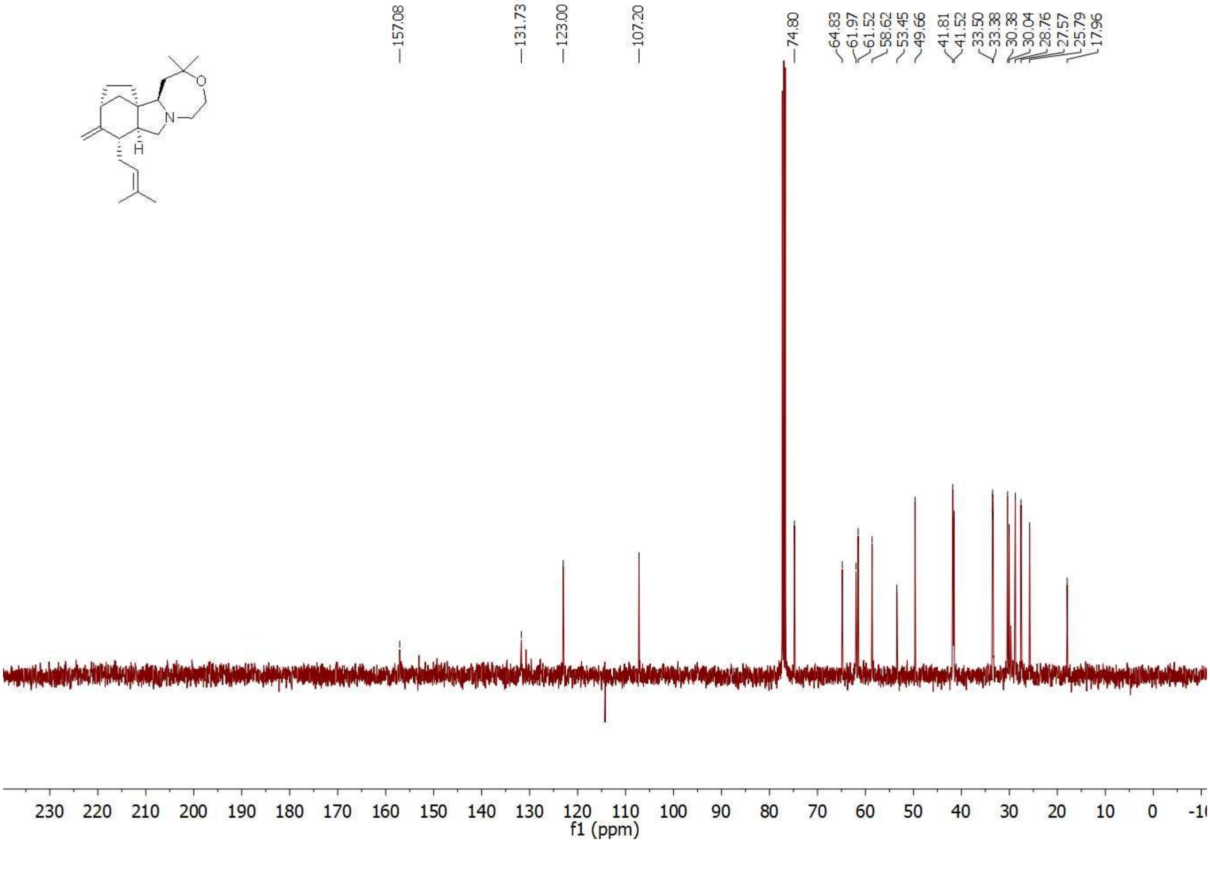

^1H NMR (400 MHz, acetone-d_6) and ^{13}C NMR (101 MHz, acetone-d_6) spectra for **1**

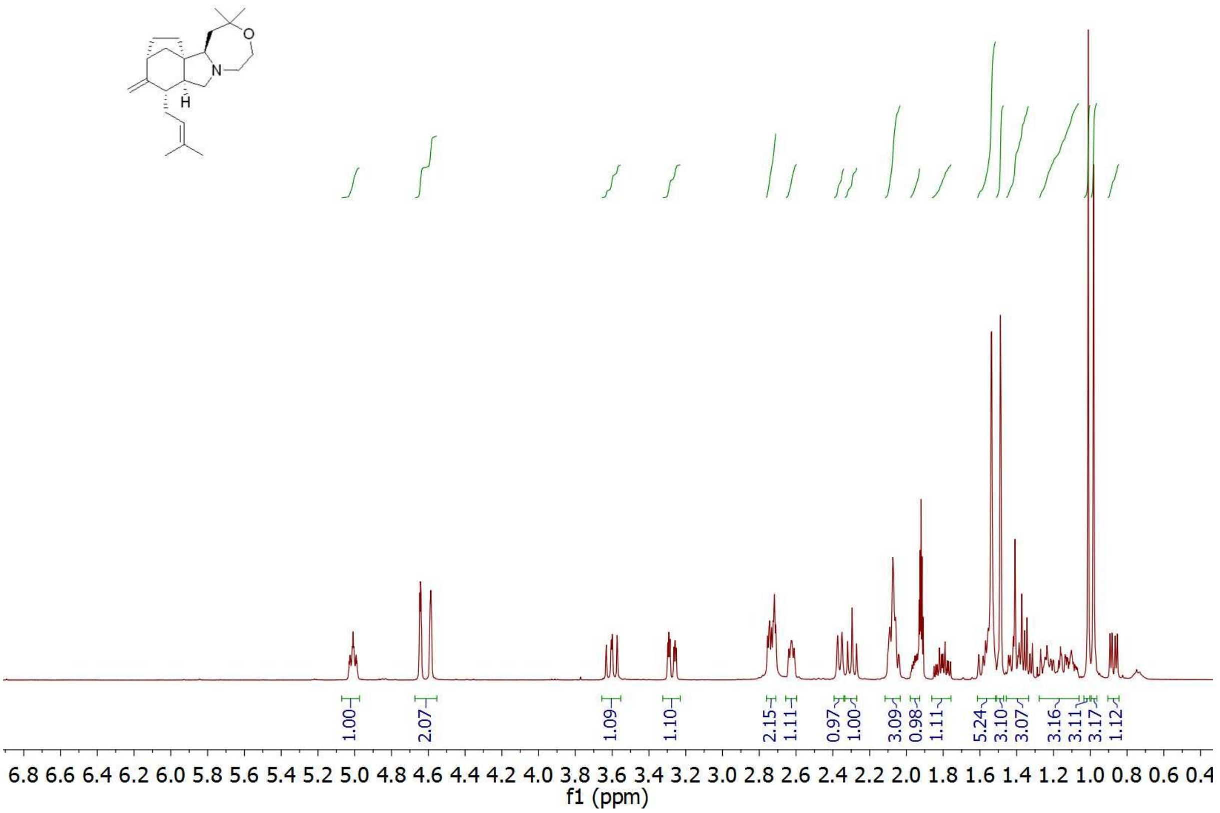

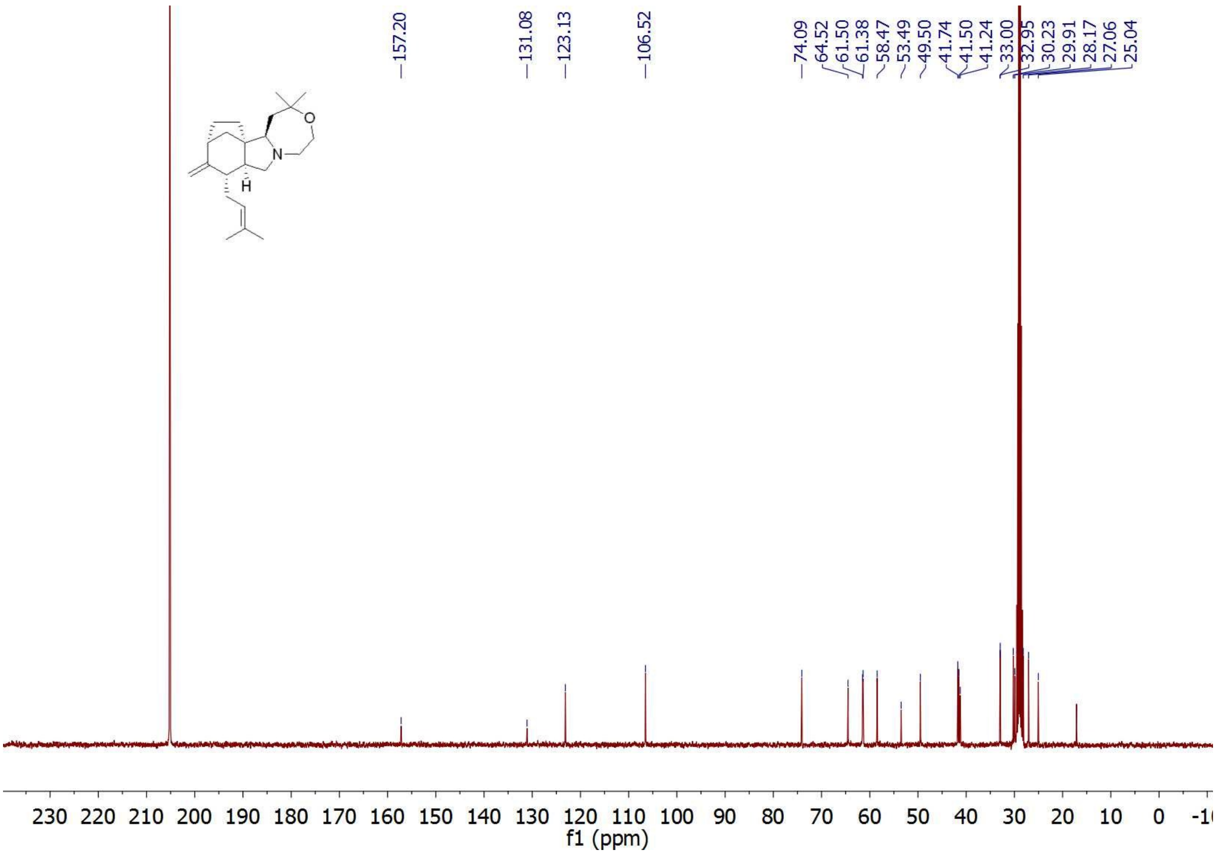

^{1}H NMR (400 MHz, acetone-d$_6$) and DEPTq (101 MHz, acetone-d$_6$) spectra for **1-HCl**

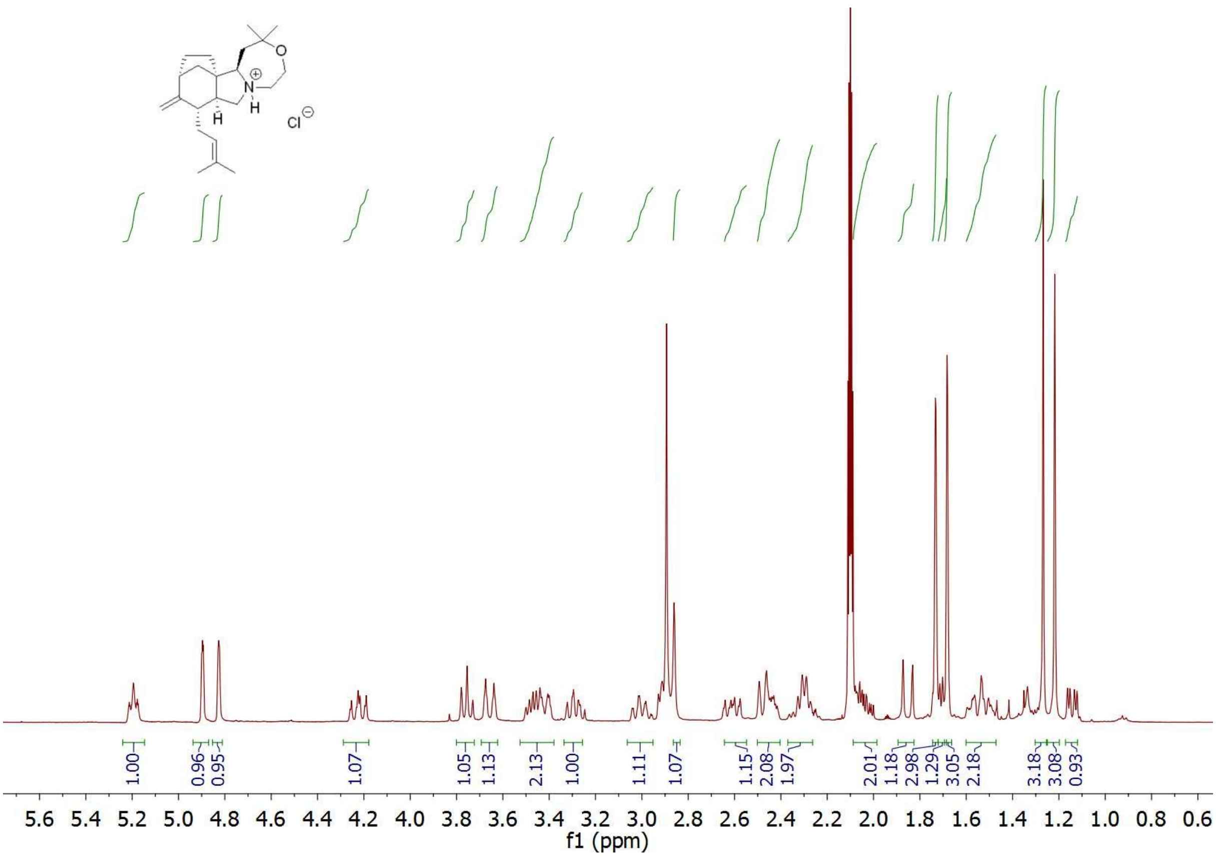

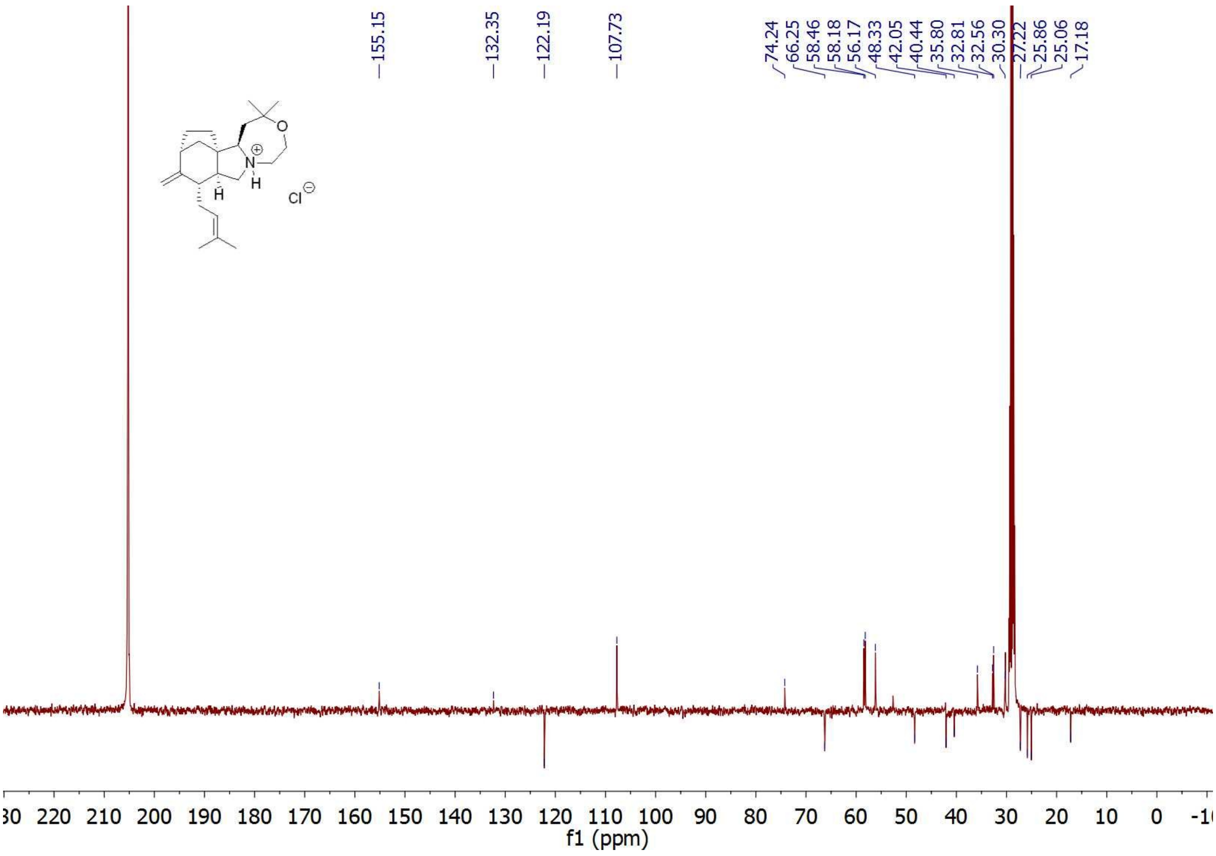

^{1}H NMR (400 MHz, $CDCl_3$) and DEPTq (101 MHz, $CDCl_3$) spectra for **1-AcOH**

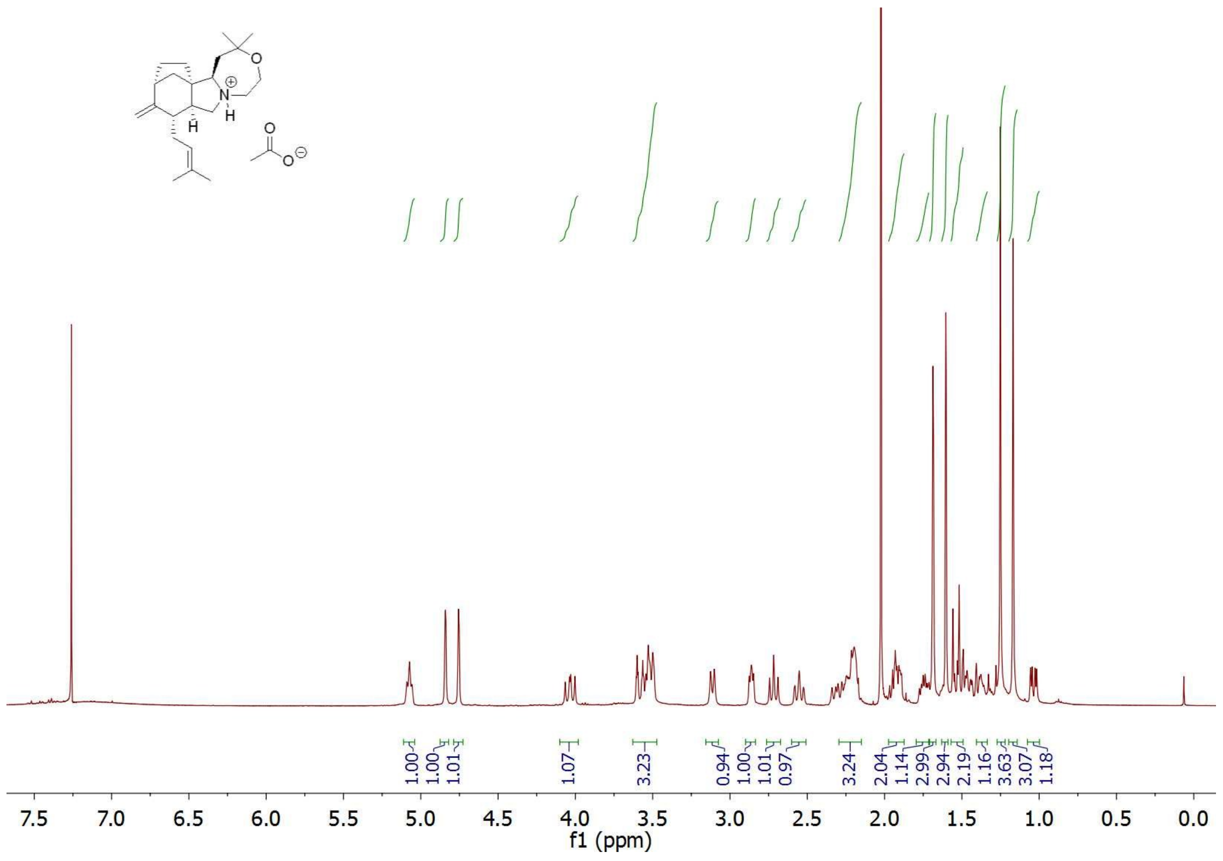

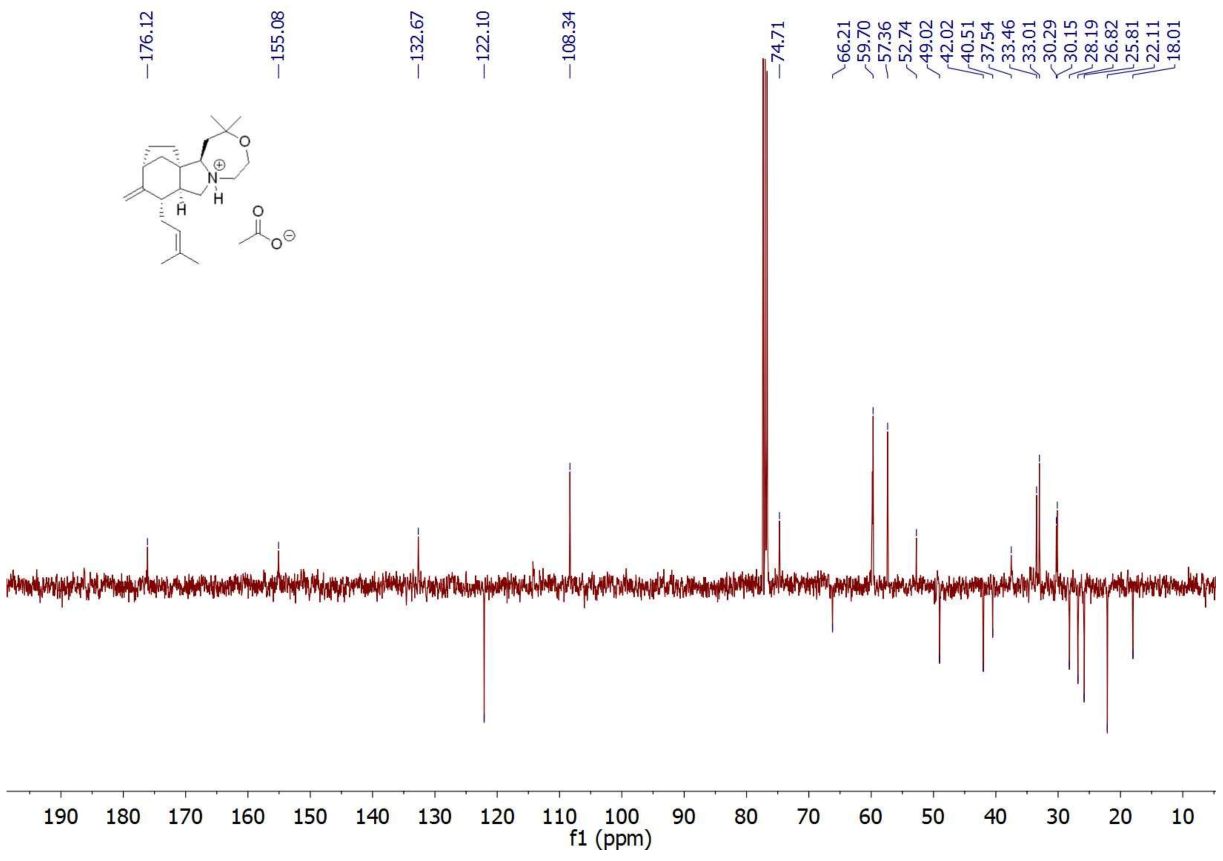

^{1}H NMR (400 MHz, $CDCl_3$) and DEPTq (101 MHz, $CDCl_3$) spectra for **25**

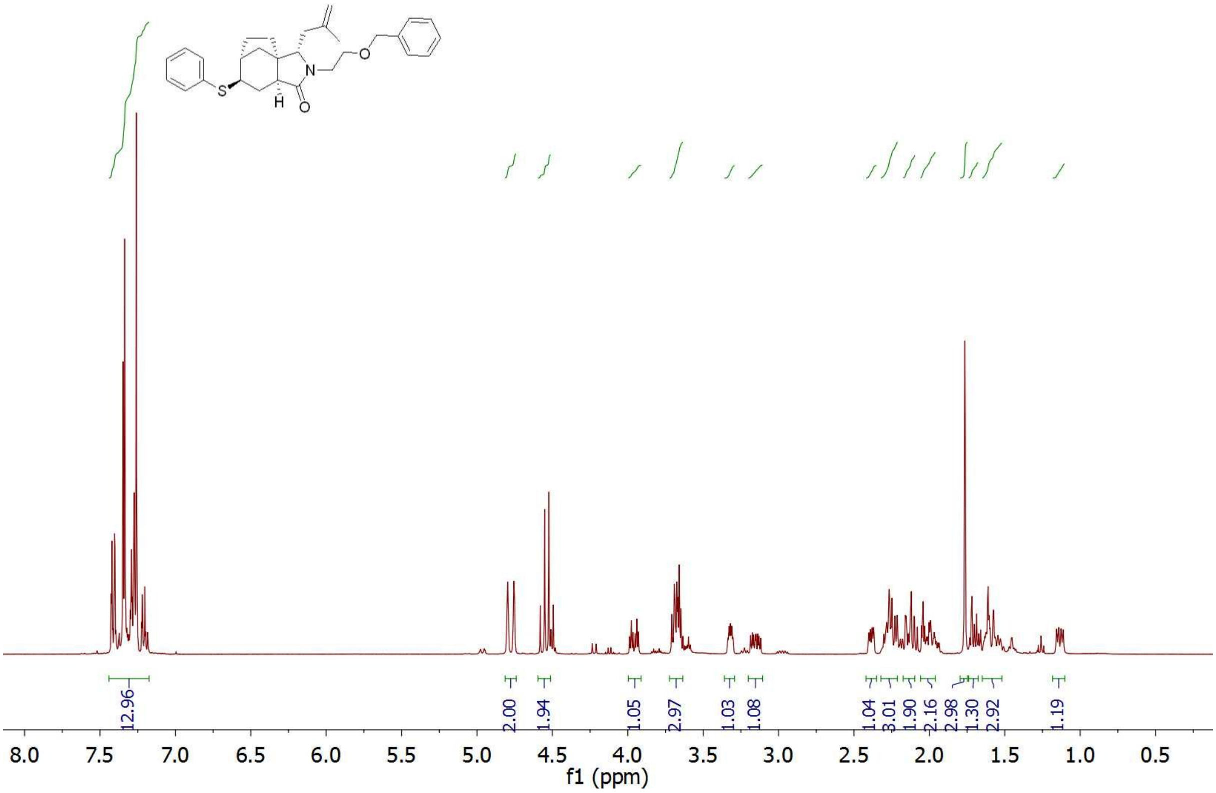

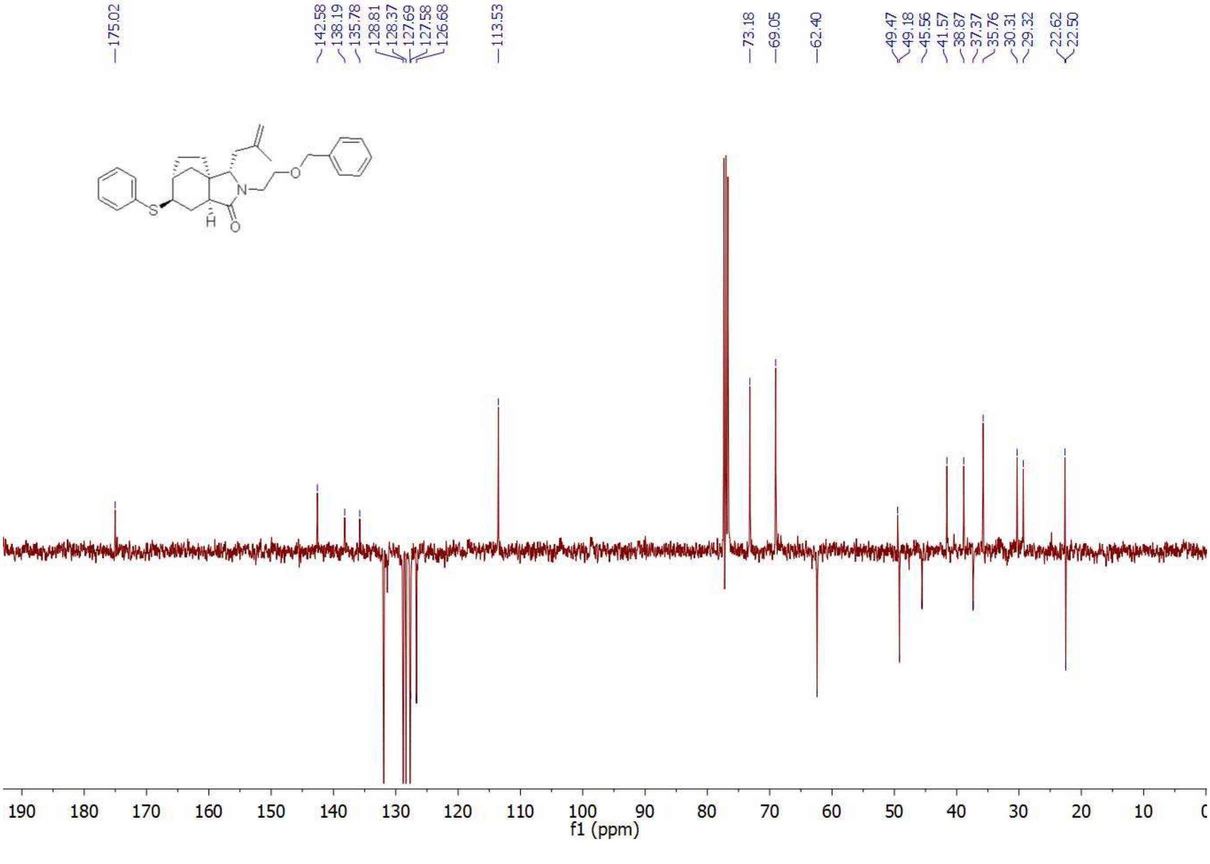

^{1}H NMR (400 MHz, $CDCl_3$) and DEPTq (101 MHz, $CDCl_3$) spectra for **27**

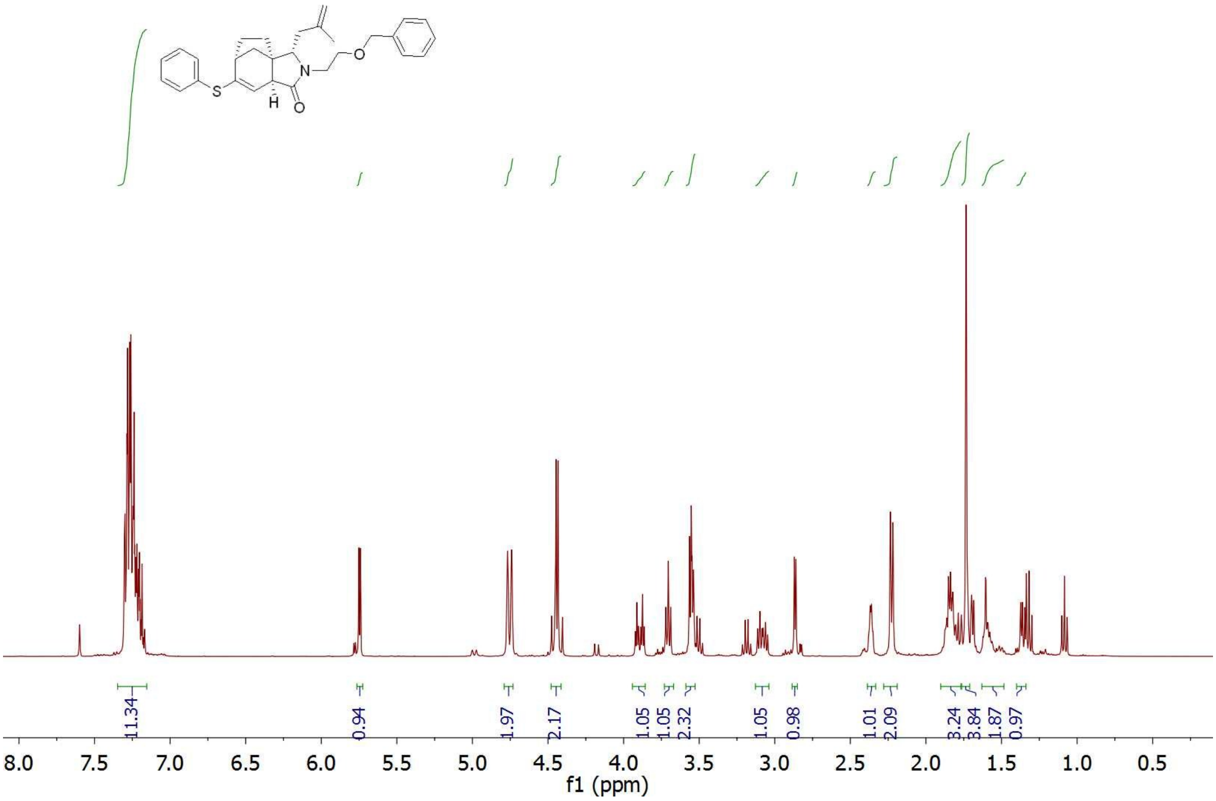

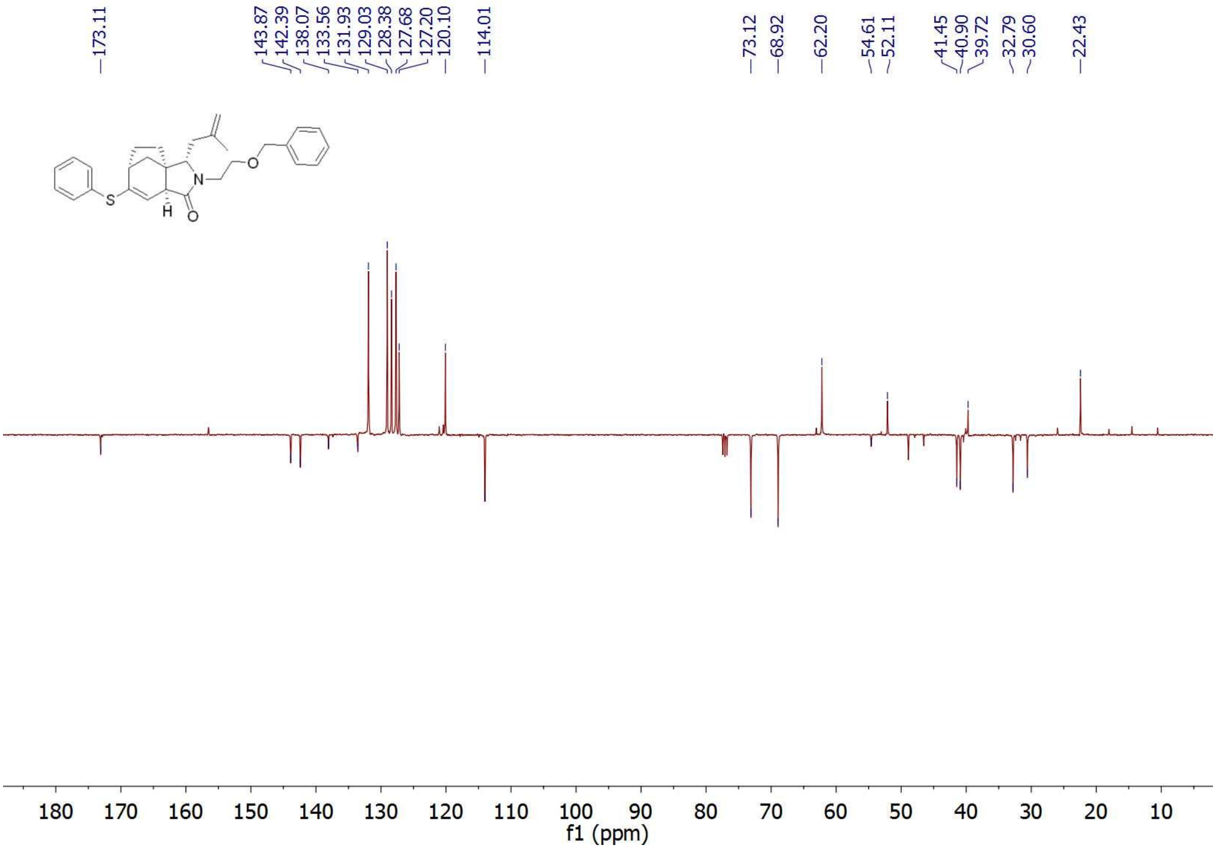

^{1}H NMR (400 MHz, $CDCl_3$) and DEPTq (101 MHz, $CDCl_3$) spectra for **28**

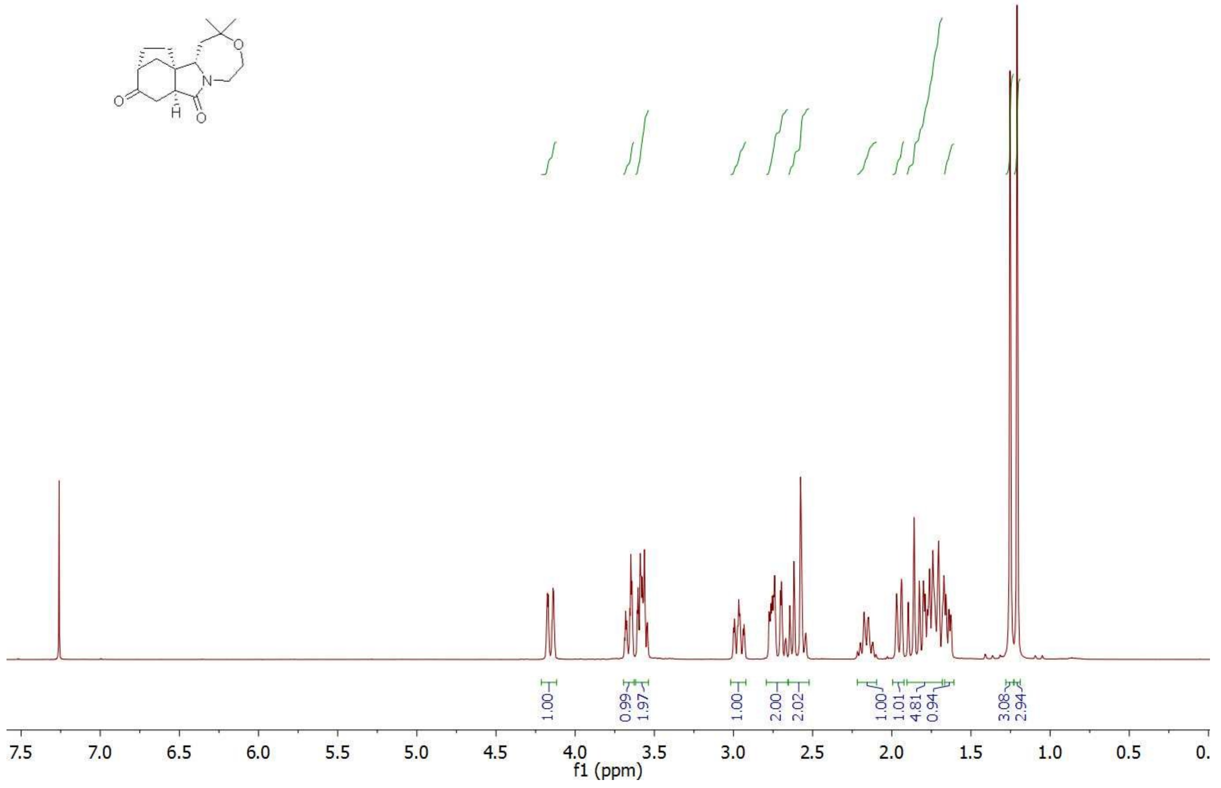

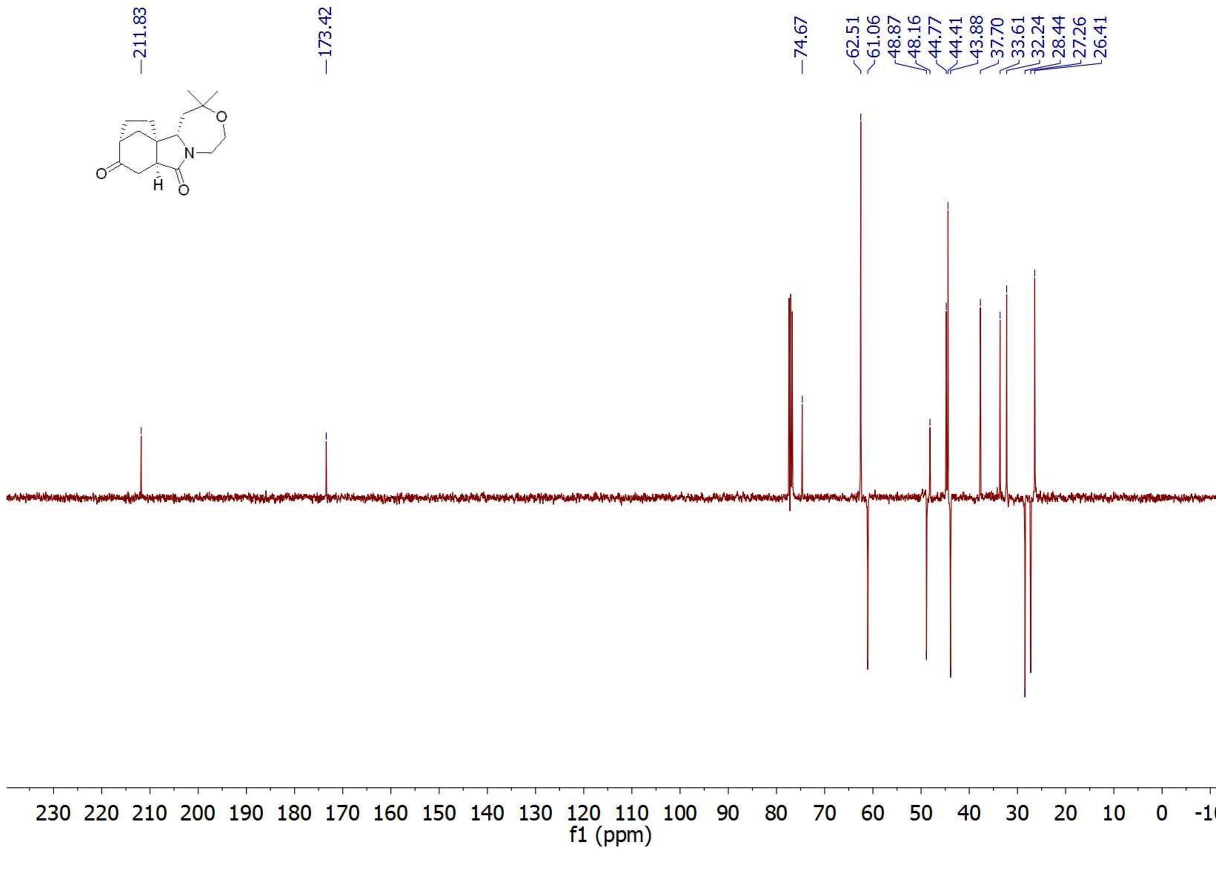

^{1}H NMR (400 MHz, $CDCl_3$) and DEPTq (101 MHz, $CDCl_3$) spectra for **29**

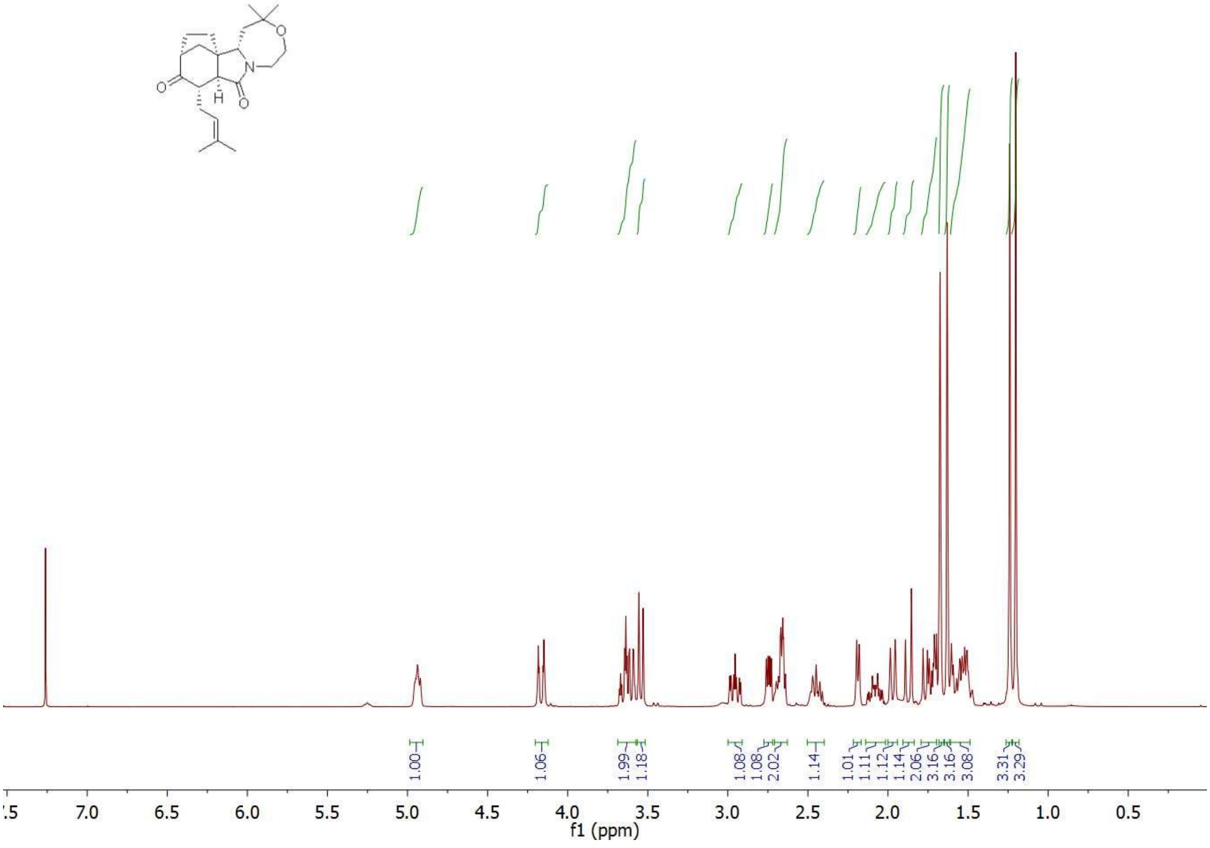

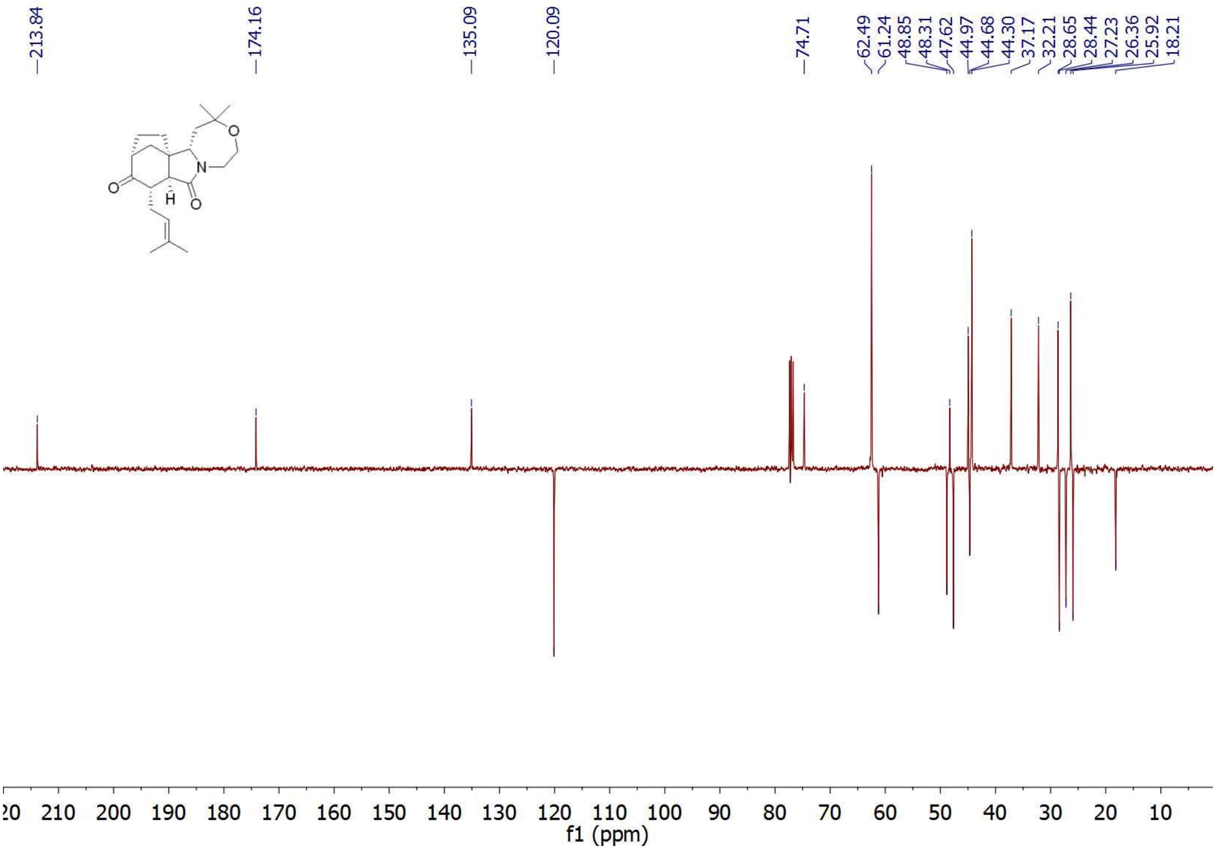

^{1}H NMR (400 MHz, $CDCl_3$) and DEPTq (101 MHz, $CDCl_3$) spectra for **30**

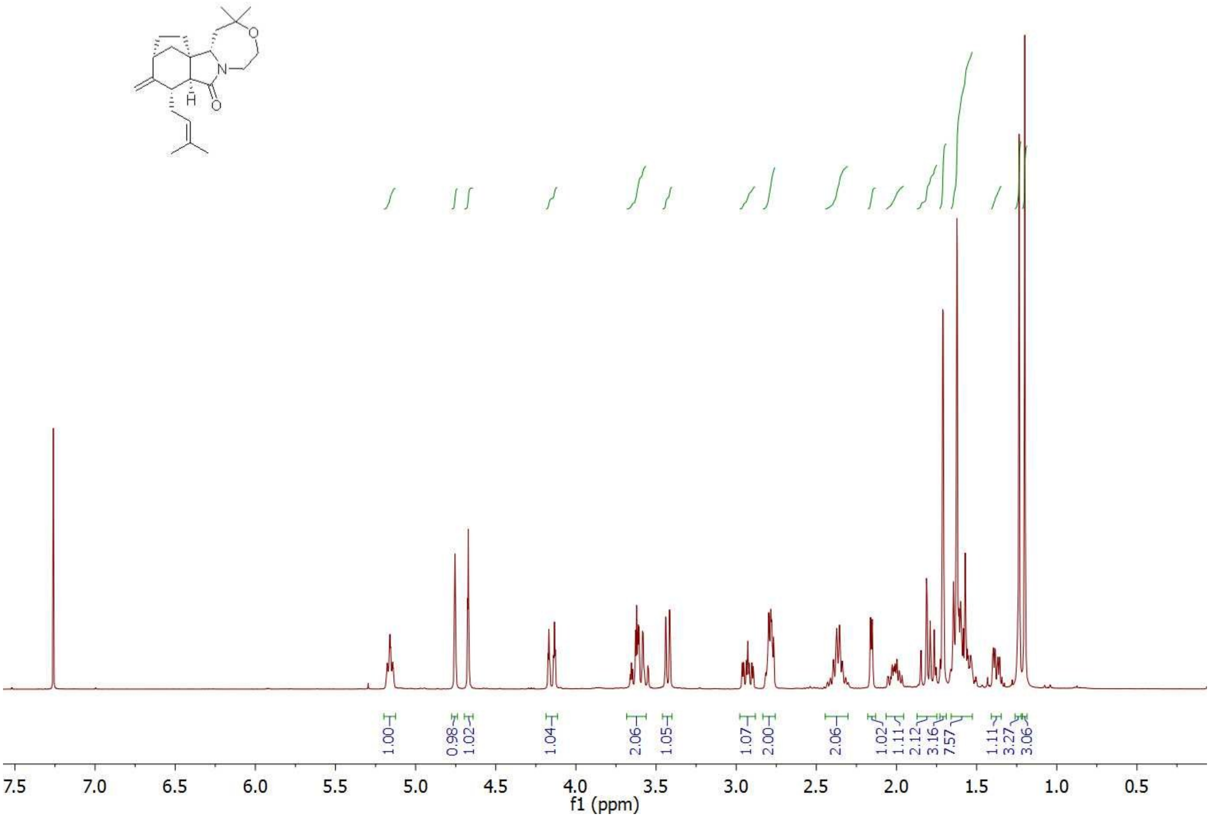

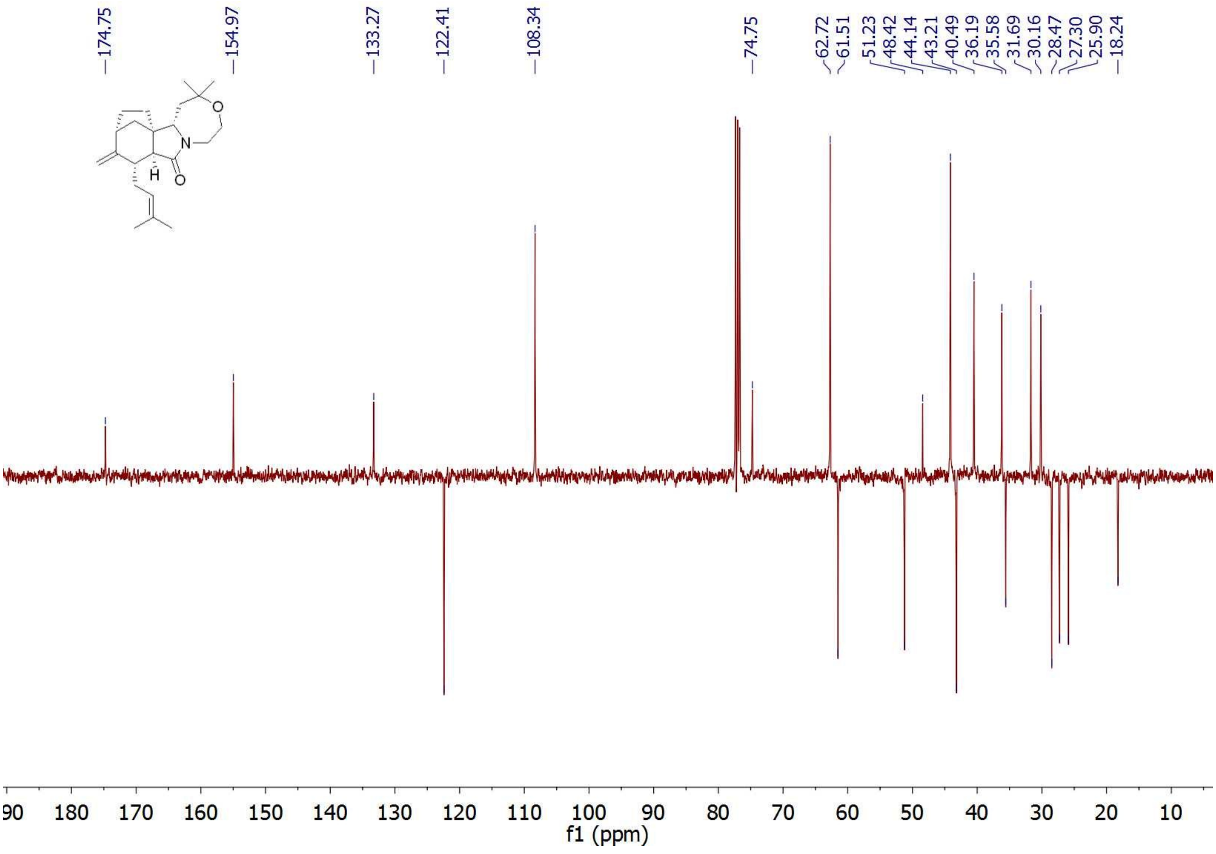

^{1}H NMR (400 MHz, $CDCl_3$) and DEPTq (101 MHz, $CDCl_3$) spectra for **epi-1**

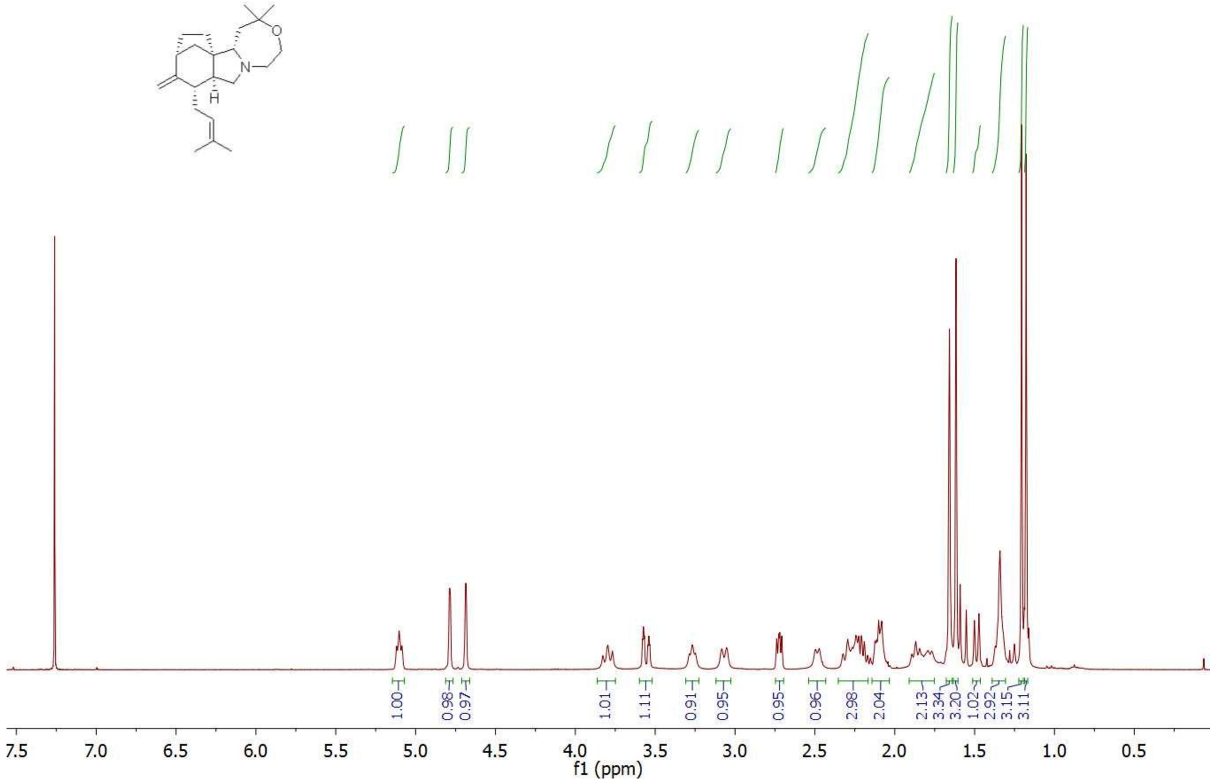

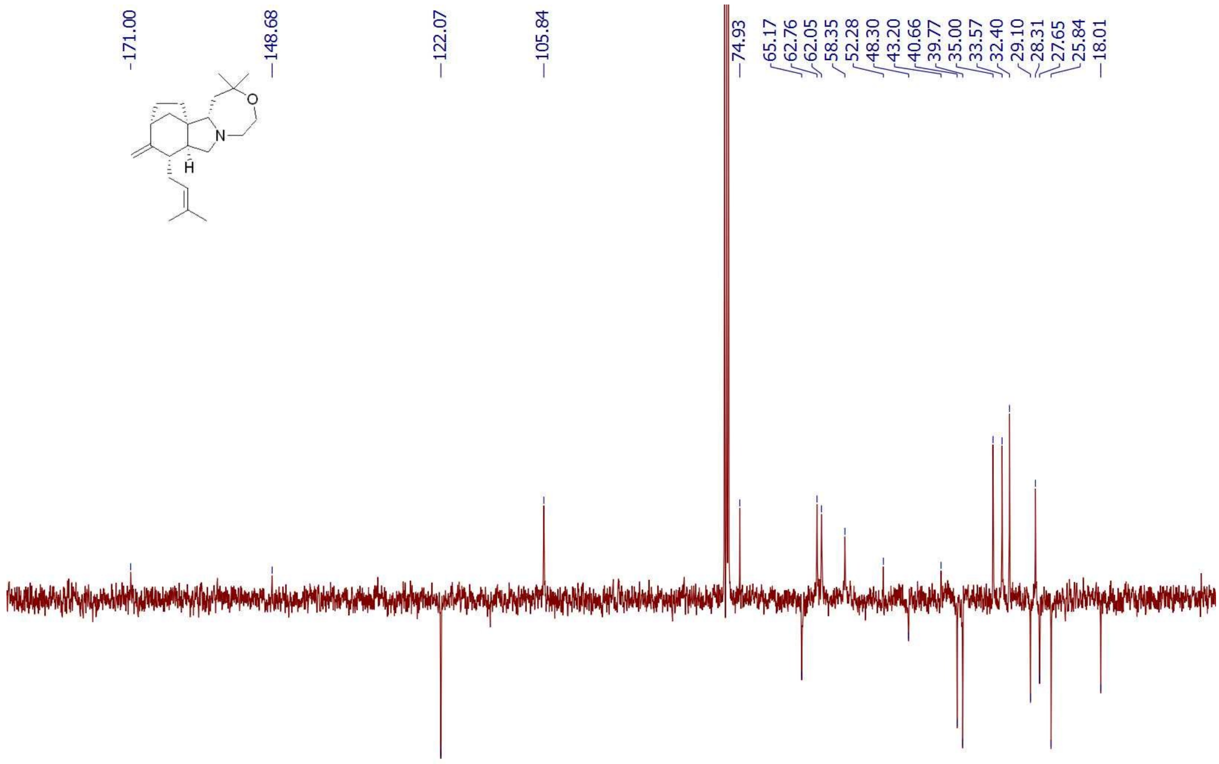

^{1}H NMR (400 MHz, $CDCl_3$) and DEPTq (101 MHz, $CDCl_3$) spectra for **31**

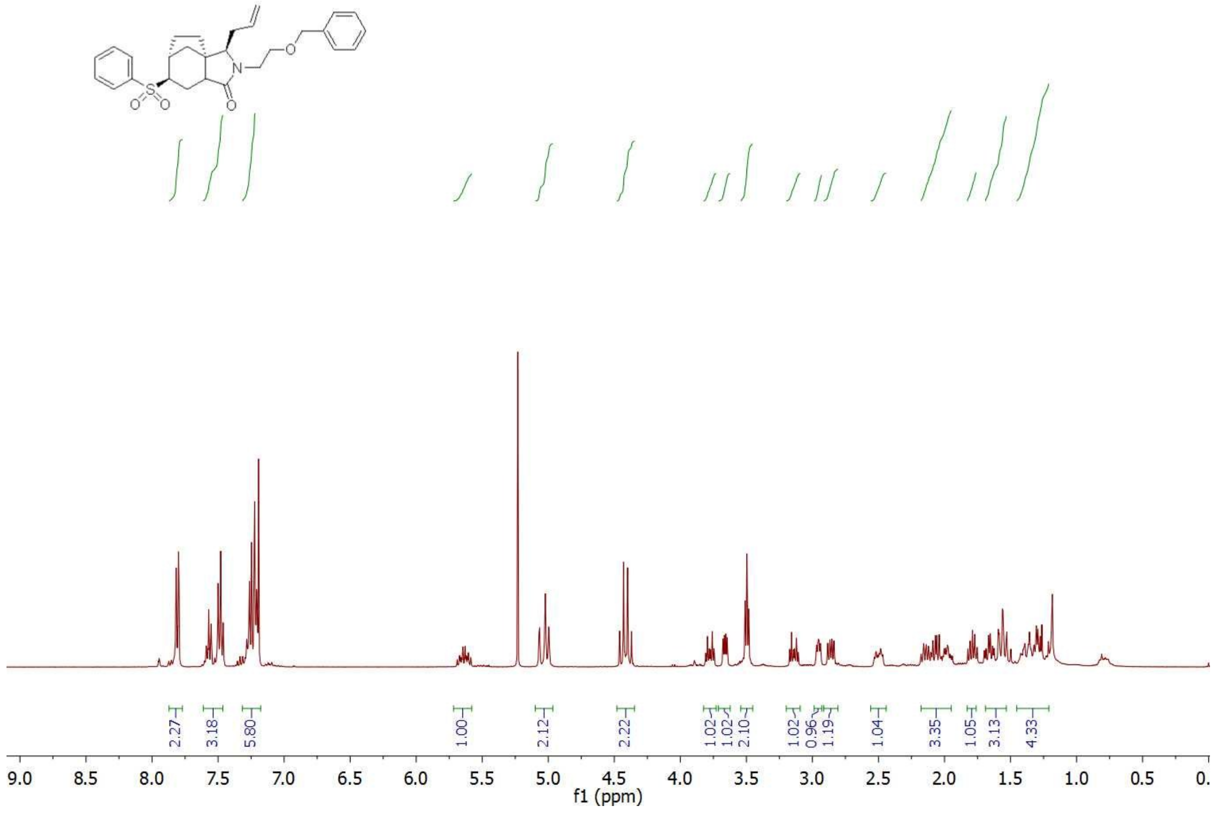

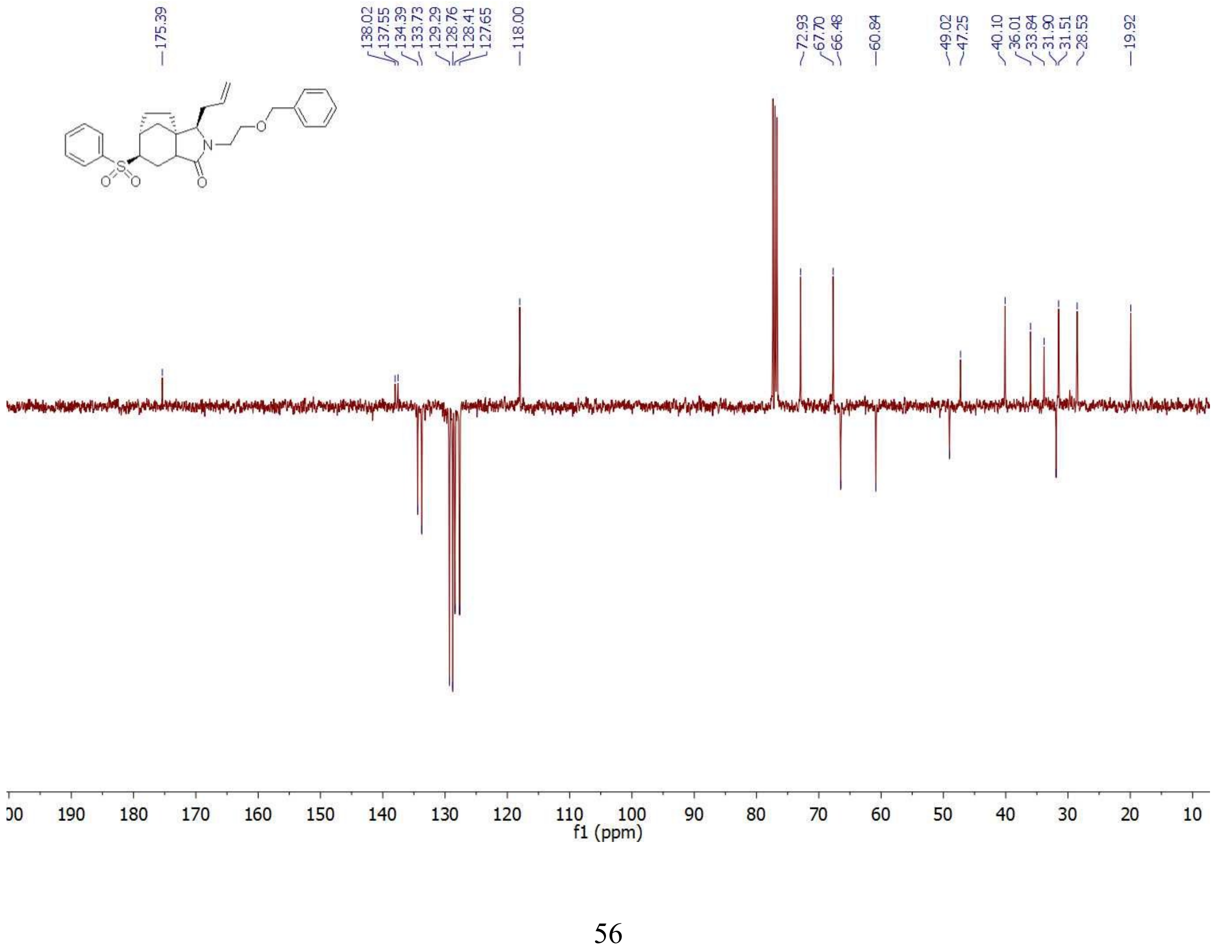

^{1}H NMR (400 MHz, $CDCl_3$) and DEPTQq(101 MHz, $CDCl_3$) spectra for **32**

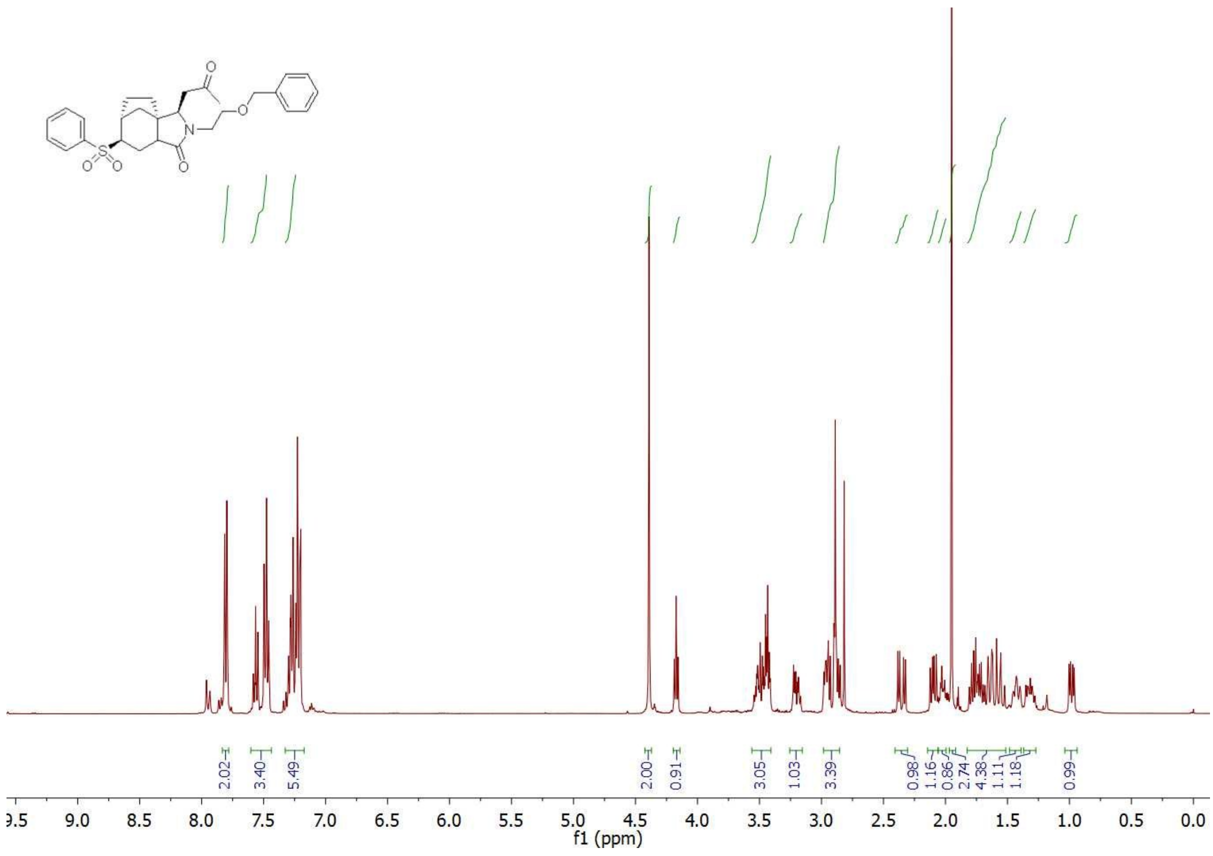

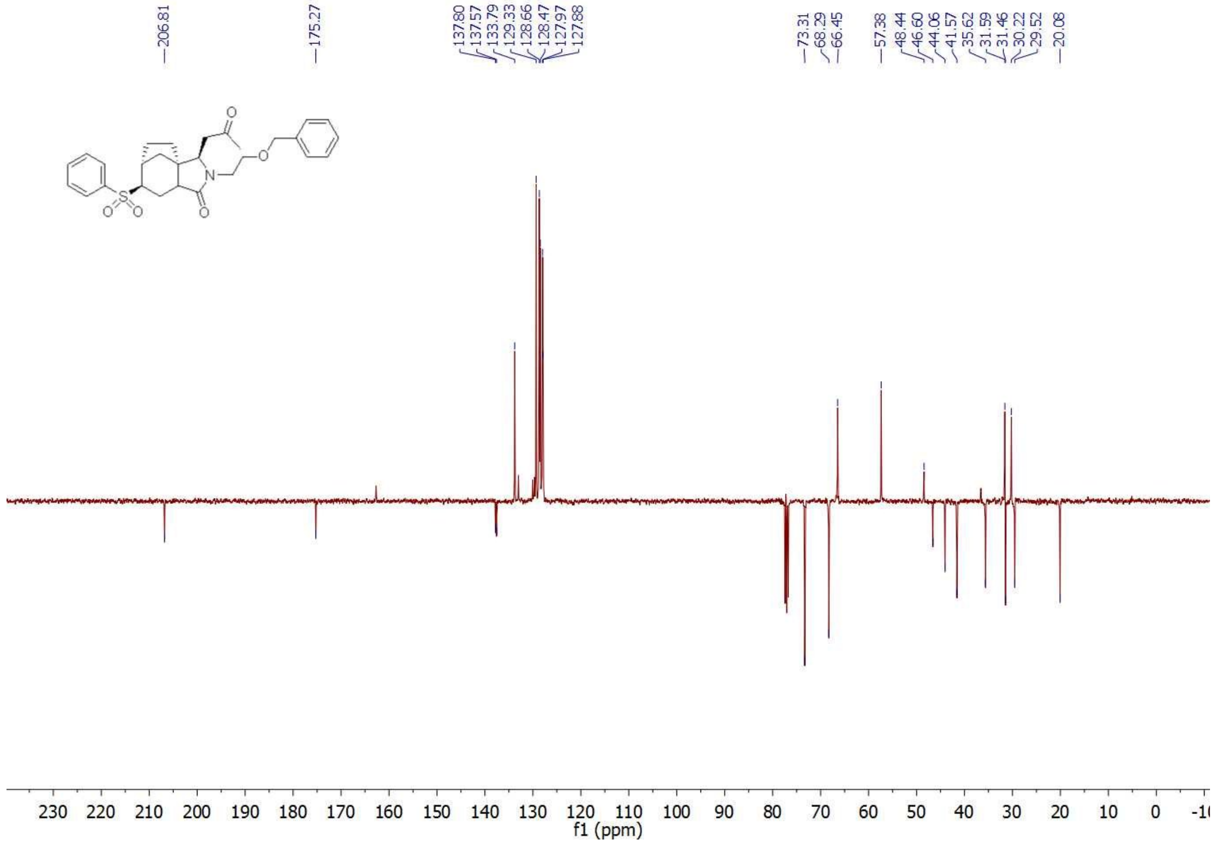

^{1}H NMR (400 MHz, $CDCl_3$) and DEPTq (101 MHz, $CDCl_3$) spectra for **33**

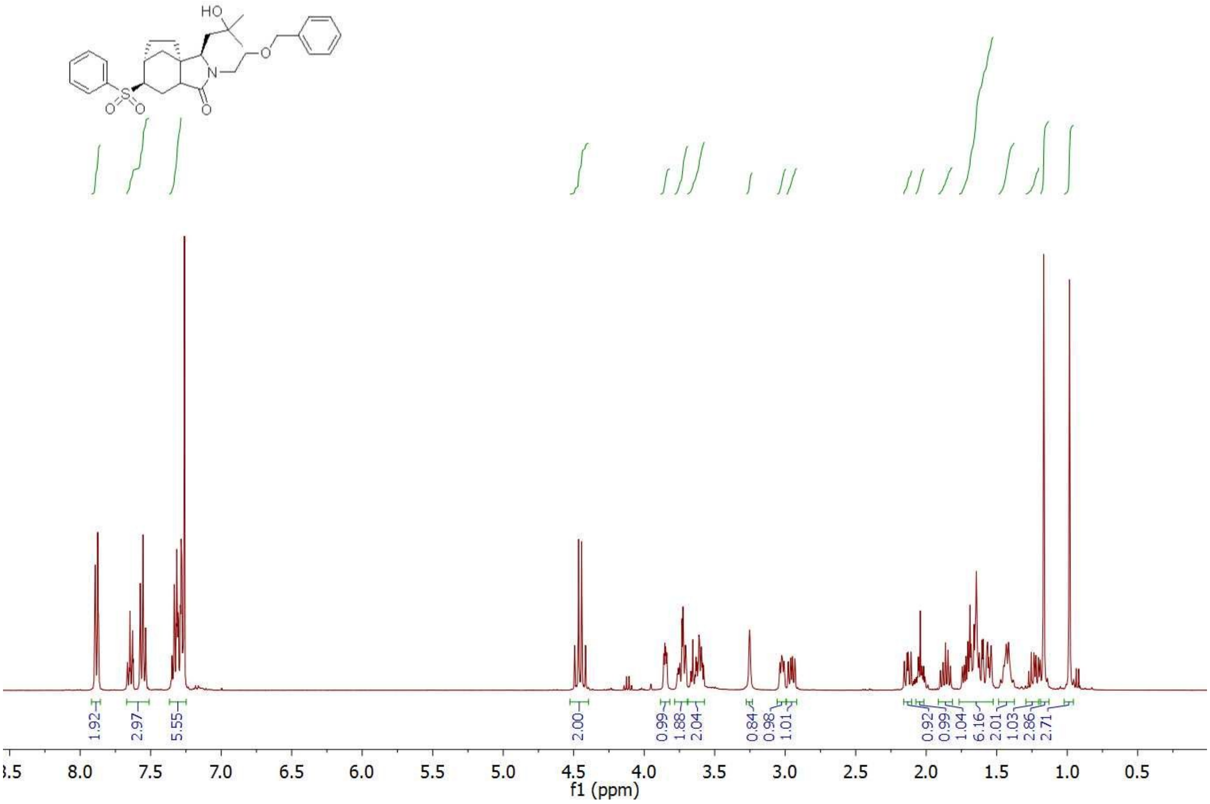

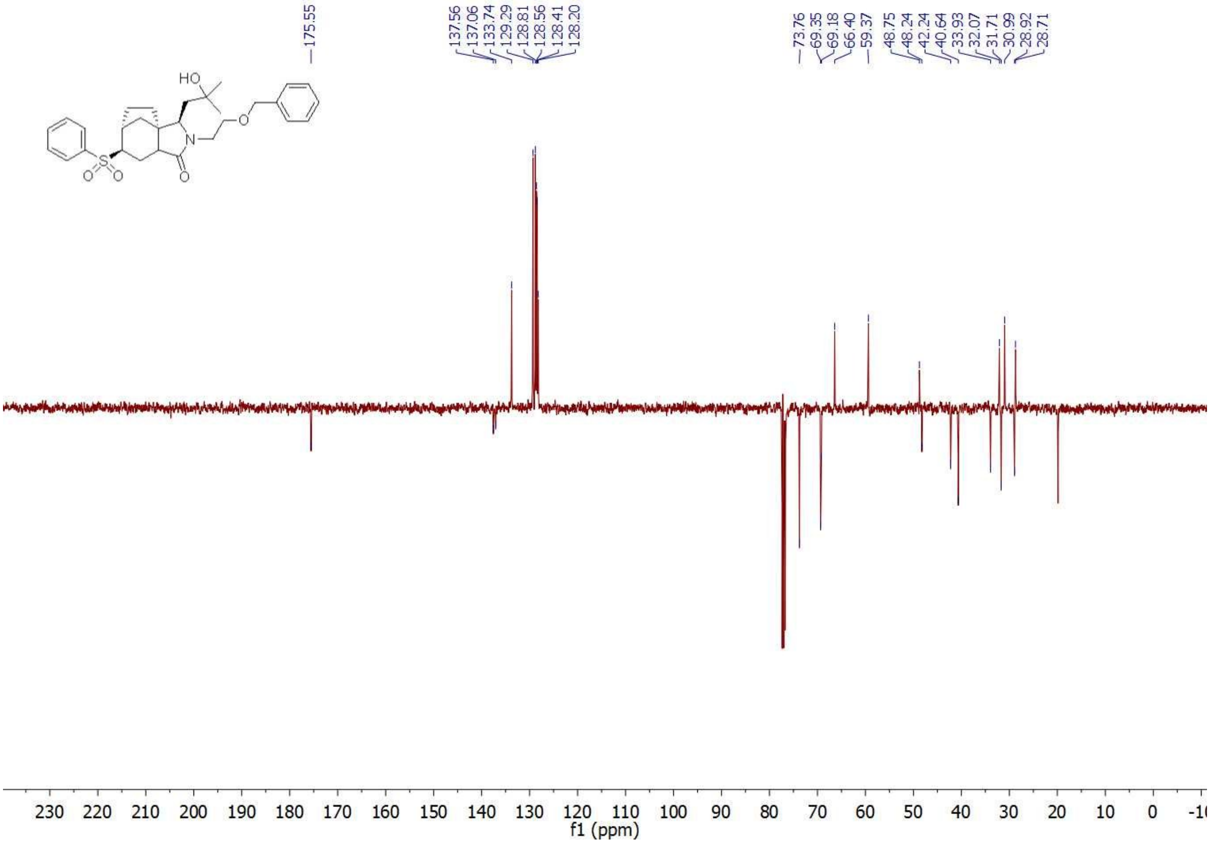

^1H NMR (400 MHz, $CDCl_3$) and DEPTq (101 MHz, $CDCl_3$) spectra for **34**

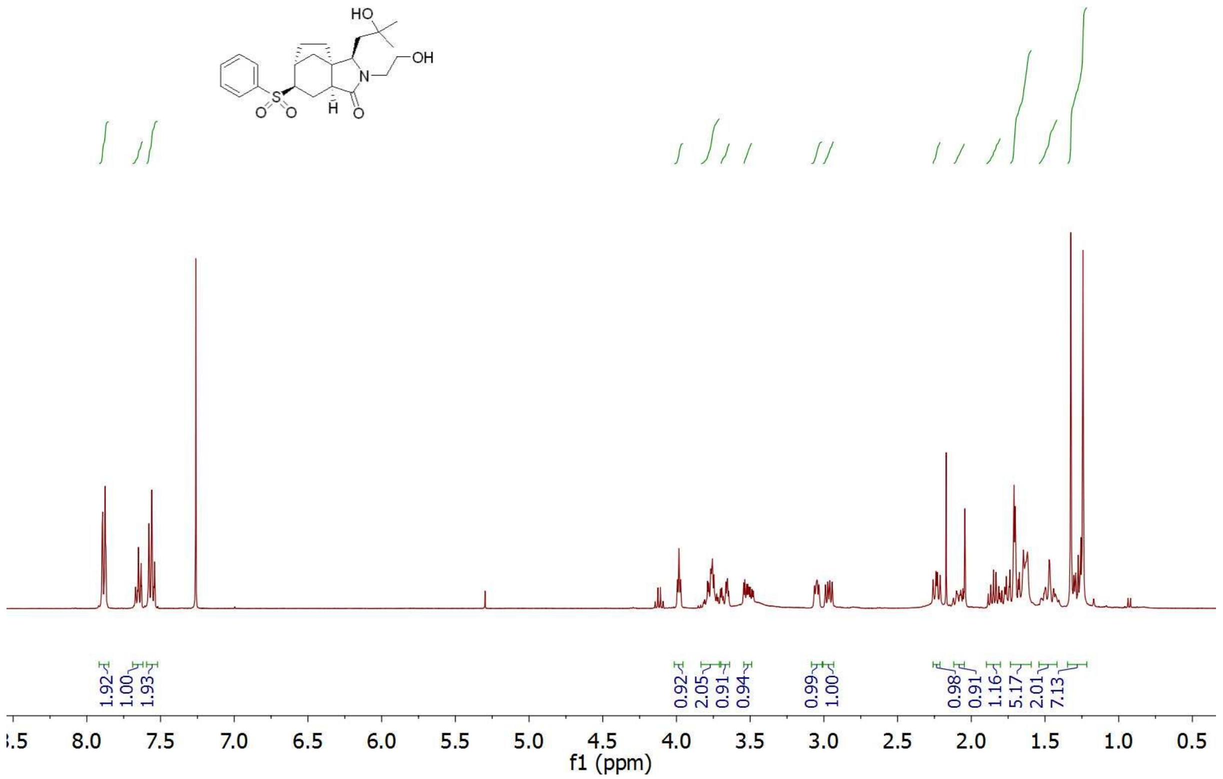

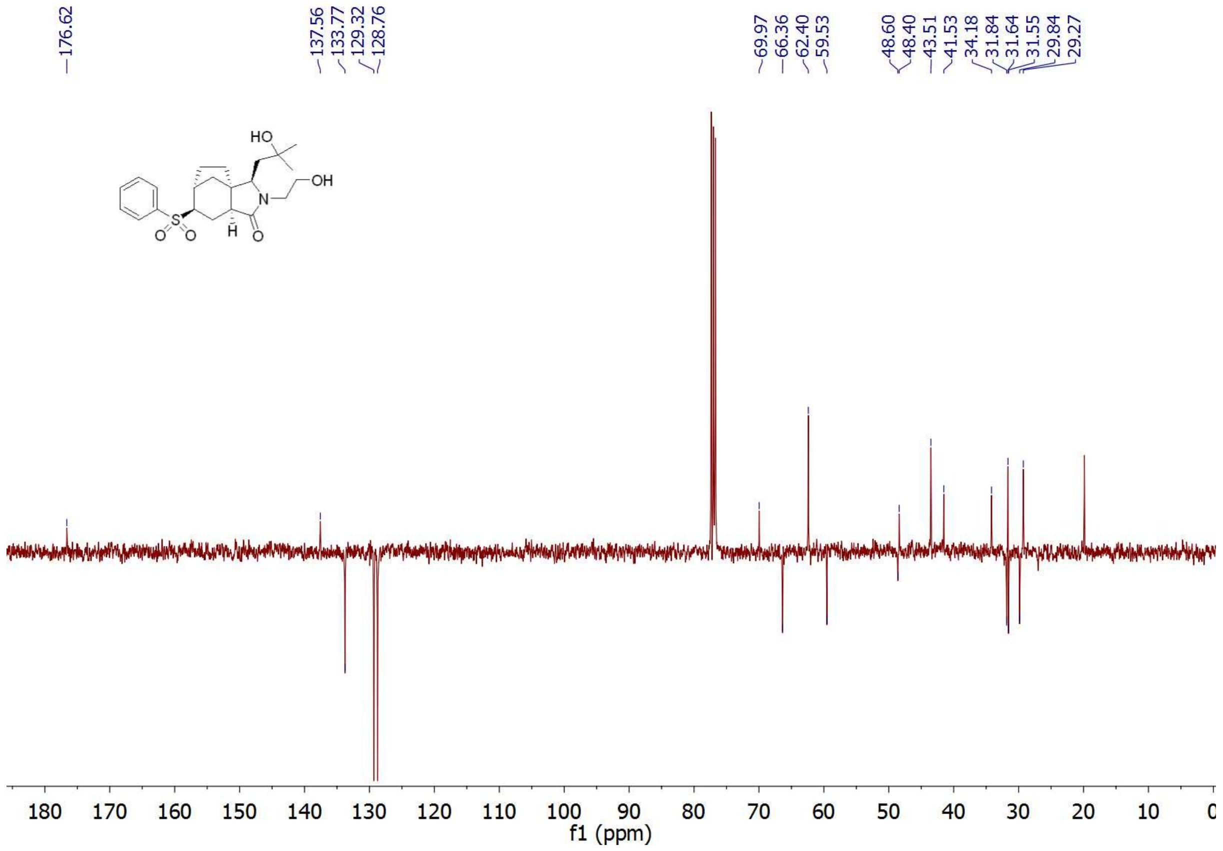

^{1}H NMR (400 MHz, $CDCl_3$) and DEPTq (101 MHz, $CDCl_3$) spectra for **35**

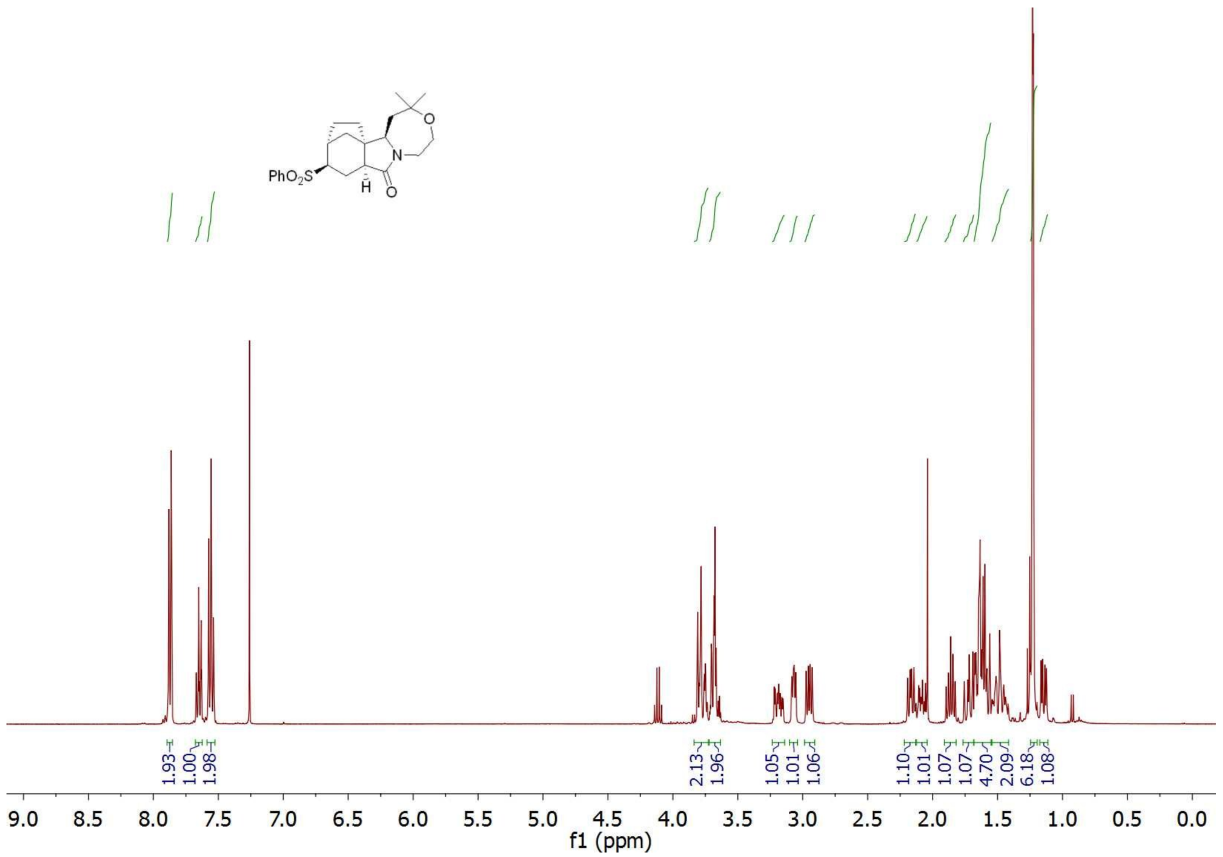

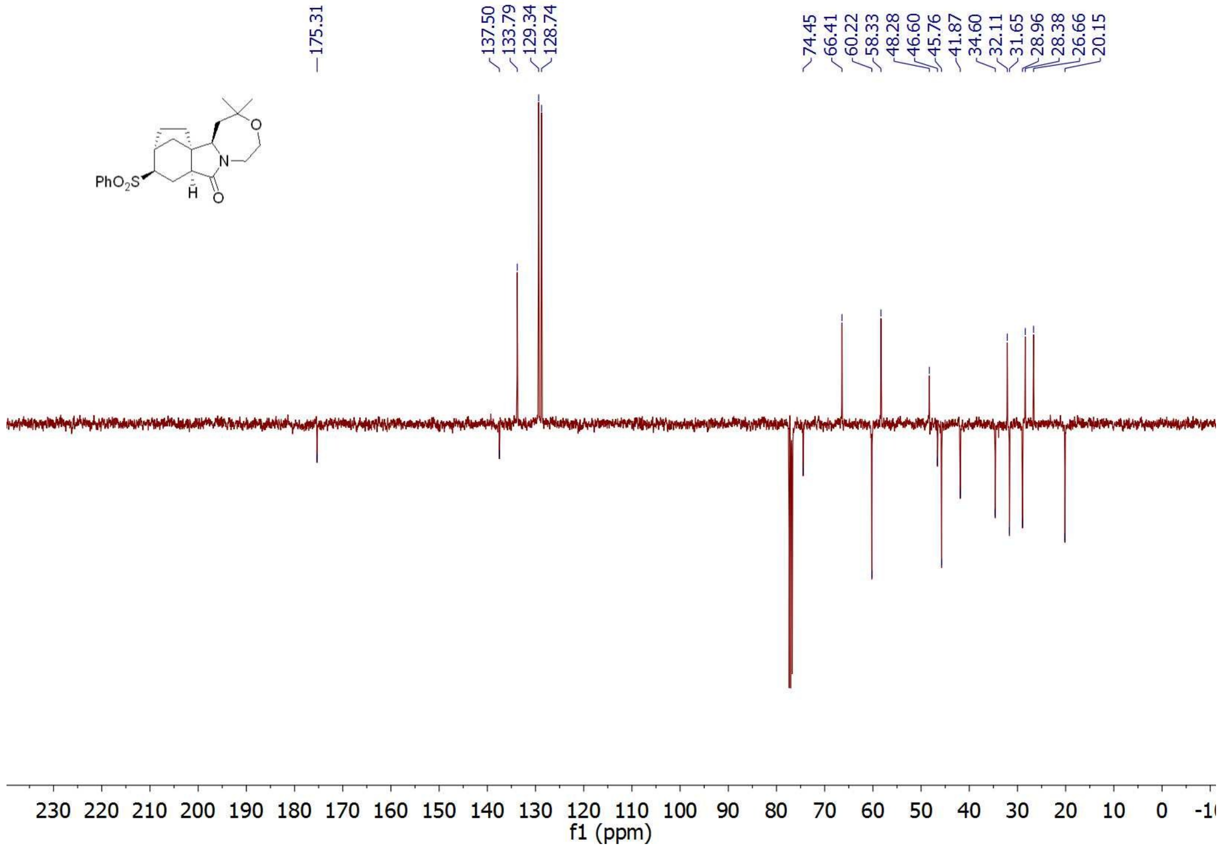

^{1}H NMR (400 MHz, $CDCl_3$) and DEPTq (101 MHz, $CDCl_3$) spectra for **23**

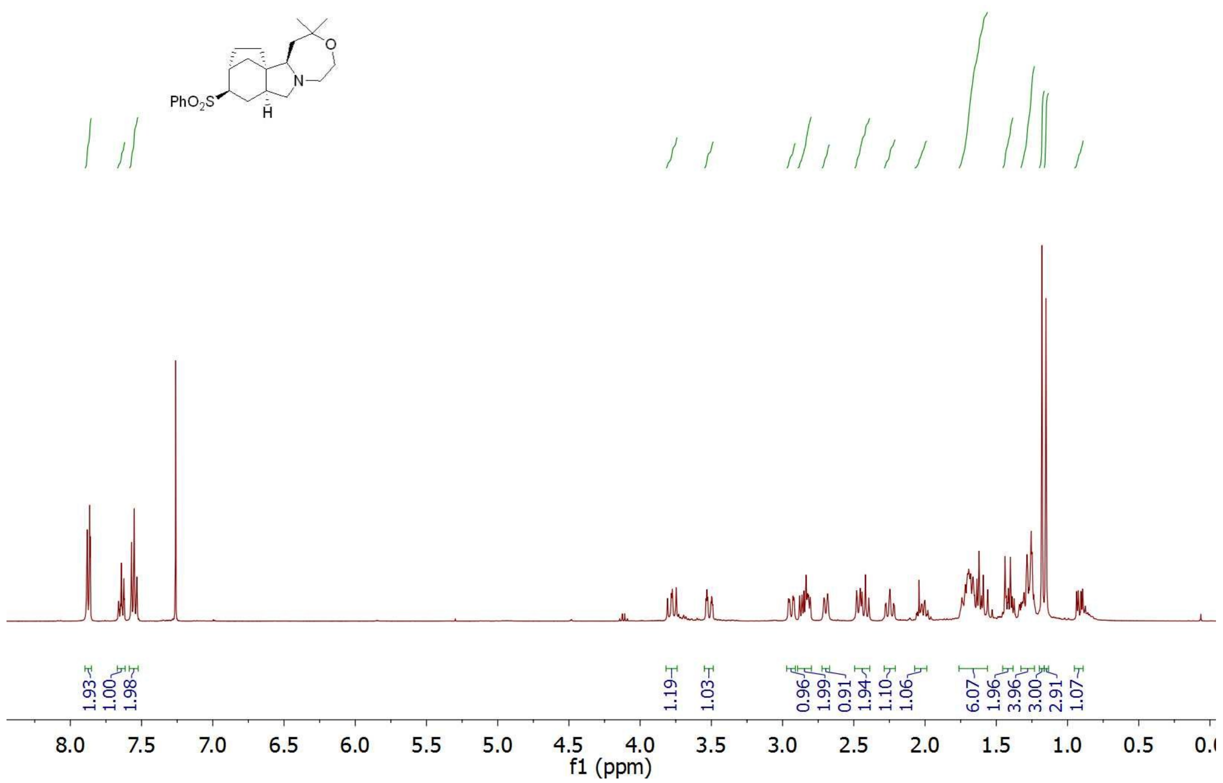

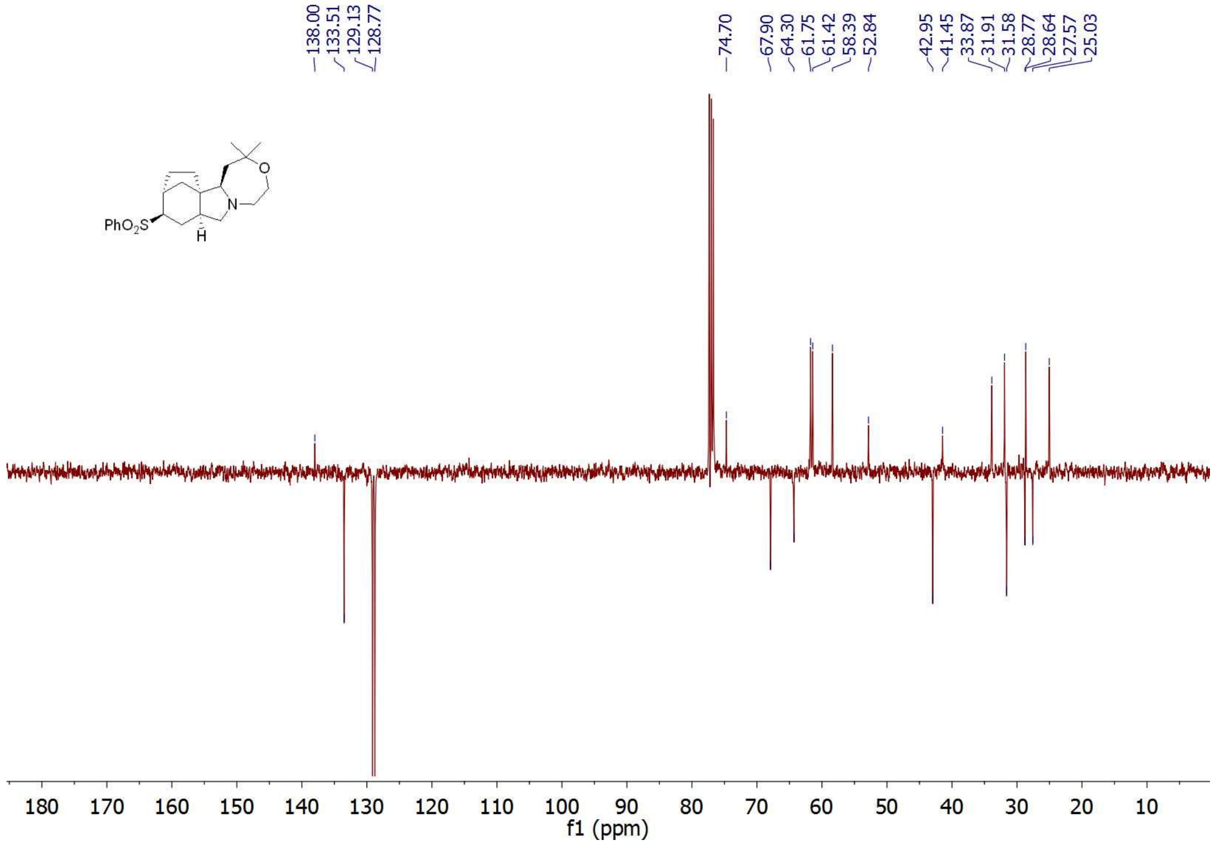

Supplement: Supplementary file 1 [file SC-008-C6SC05627J-s001.pdf]
